# Supplementary material for: Genome-wide identification, characterization and gene expression of BES1 transcription factor family in grapevine (Vitis vinifera L.)
Source: Sci Rep. 2023 Jan 5;13:240. doi: 10.1038/s41598-022-24407-y (PMC9816167; doi:10.1038/s41598-022-24407-y)
Supplement: Supplementary file 3 — Supplementary Information. [file 41598_2022_24407_MOESM3_ESM.zip › Vvi_Vvi/Vitis_vinifera.PN40024.v4.dna_sm.toplevel.fa.vs.Vitis_vinifera.PN40024.v4.dna_sm.toplevel.fa.html/7.html]

|  |  |  |  |  |  |  |  |  |  |  |  |  |  |
| --- | --- | --- | --- | --- | --- | --- | --- | --- | --- | --- | --- | --- | --- |
| Duplication depth | Reference chromosome | Collinear blocks | | | | | | | | | | | |
| 0 | Vitvi07g00002\_t001 |  |  |  |  |  |  |  |  |  |  |  |  |
| 0 | Vitvi07g04000\_t001 |  |  |  |  |  |  |  |  |  |  |  |  |
| 0 | Vitvi07g04001\_t001 |  |  |  |  |  |  |  |  |  |  |  |  |
| 0 | Vitvi07g04002\_t001 |  |  |  |  |  |  |  |  |  |  |  |  |
| 0 | Vitvi07g00011\_t001 |  |  |  |  |  |  |  |  |  |  |  |  |
| 0 | Vitvi07g00012\_t001 |  |  |  |  |  |  |  |  |  |  |  |  |
| 1 | Vitvi07g00013\_t001 |  | Vitvi14g00508\_t001 |  |  |  |  |  |  |  |  |  |  |
| 1 | Vitvi07g00015\_t001 |  | \| \| |  |  |  |  |  |  |  |  |  |  |
| 1 | Vitvi07g00016\_t001 |  | \| \| |  |  |  |  |  |  |  |  |  |  |
| 1 | Vitvi07g04003\_t001 |  | \| \| |  |  |  |  |  |  |  |  |  |  |
| 2 | Vitvi07g00017\_t001 |  | Vitvi14g00509\_t001 |  | Vitvi14g00509\_t001 |  |  |  |  |  |  |  |  |
| 2 | Vitvi07g00018\_t001 |  | Vitvi14g00521\_t001 |  | \| \| |  |  |  |  |  |  |  |  |
| 2 | Vitvi07g00020\_t001 |  | \| \| |  | Vitvi14g02680\_t001 |  |  |  |  |  |  |  |  |
| 3 | Vitvi07g00021\_t001 |  | \| \| |  | \| \| |  | Vitvi05g00648\_t001 |  |  |  |  |  |  |
| 3 | Vitvi07g04004\_t001 |  | \| \| |  | \| \| |  | \| \| |  |  |  |  |  |  |
| 3 | Vitvi07g00024\_t001 |  | Vitvi14g00537\_t001 |  | \| \| |  | Vitvi05g00642\_t001 |  |  |  |  |  |  |
| 3 | Vitvi07g00025\_t001 |  | \| \| |  | \| \| |  | Vitvi05g00641\_t001 |  |  |  |  |  |  |
| 3 | Vitvi07g02066\_t001 |  | Vitvi14g02049\_t001 |  | \| \| |  | \| \| |  |  |  |  |  |  |
| 3 | Vitvi07g00026\_t001 |  | Vitvi14g00540\_t001 |  | \| \| |  | \| \| |  |  |  |  |  |  |
| 2 | Vitvi07g00027\_t001 |  |  |  | \| \| |  | \| \| |  |  |  |  |  |  |
| 2 | Vitvi07g00029\_t001 |  |  |  | \| \| |  | \| \| |  |  |  |  |  |  |
| 2 | Vitvi07g00030\_t001 |  |  |  | Vitvi14g02667\_t001 |  | Vitvi05g01906\_t001 |  |  |  |  |  |  |
| 2 | Vitvi07g00031\_t001 |  |  |  | Vitvi14g00490\_t001 |  | Vitvi05g00634\_t001 |  |  |  |  |  |  |
| 2 | Vitvi07g00032\_t001 |  |  |  | \| \| |  | Vitvi05g00633\_t002 |  |  |  |  |  |  |
| 2 | Vitvi07g00034\_t001 |  |  |  | \| \| |  | \| \| |  |  |  |  |  |  |
| 2 | Vitvi07g00035\_t001 |  |  |  | Vitvi14g00489\_t001 |  | \| \| |  |  |  |  |  |  |
| 2 | Vitvi07g04005\_t001 |  |  |  | \| \| |  | \| \| |  |  |  |  |  |  |
| 2 | Vitvi07g04006\_t001 |  |  |  | \| \| |  | \| \| |  |  |  |  |  |  |
| 2 | Vitvi07g04007\_t001 |  |  |  | \| \| |  | \| \| |  |  |  |  |  |  |
| 2 | Vitvi07g04008\_t001 |  |  |  | \| \| |  | \| \| |  |  |  |  |  |  |
| 2 | Vitvi07g04009\_t001 |  |  |  | \| \| |  | \| \| |  |  |  |  |  |  |
| 2 | Vitvi07g04010\_t001 |  |  |  | \| \| |  | \| \| |  |  |  |  |  |  |
| 2 | Vitvi07g04011\_t001 |  |  |  | \| \| |  | \| \| |  |  |  |  |  |  |
| 2 | Vitvi07g02092\_t001 |  |  |  | \| \| |  | \| \| |  |  |  |  |  |  |
| 2 | Vitvi07g04012\_t001 |  |  |  | \| \| |  | \| \| |  |  |  |  |  |  |
| 2 | Vitvi07g02094\_t001 |  |  |  | \| \| |  | \| \| |  |  |  |  |  |  |
| 2 | Vitvi07g04013\_t001 |  |  |  | \| \| |  | \| \| |  |  |  |  |  |  |
| 2 | Vitvi07g00036\_t003 |  |  |  | \| \| |  | \| \| |  |  |  |  |  |  |
| 2 | Vitvi07g00037\_t002 |  |  |  | \| \| |  | \| \| |  |  |  |  |  |  |
| 2 | Vitvi07g04014\_t001 |  |  |  | \| \| |  | \| \| |  |  |  |  |  |  |
| 2 | Vitvi07g00038\_t001 |  |  |  | \| \| |  | \| \| |  |  |  |  |  |  |
| 2 | Vitvi07g00039\_t001 |  |  |  | \| \| |  | \| \| |  |  |  |  |  |  |
| 2 | Vitvi07g04015\_t001 |  |  |  | \| \| |  | \| \| |  |  |  |  |  |  |
| 2 | Vitvi07g00040\_t001 |  |  |  | \| \| |  | \| \| |  |  |  |  |  |  |
| 2 | Vitvi07g00041\_t001 |  |  |  | \| \| |  | \| \| |  |  |  |  |  |  |
| 2 | Vitvi07g00042\_t001 |  |  |  | Vitvi14g00483\_t001 |  | Vitvi05g00630\_t001 |  |  |  |  |  |  |
| 2 | Vitvi07g00044\_t001 |  |  |  | \| \| |  | \| \| |  |  |  |  |  |  |
| 2 | Vitvi07g00045\_t001 |  |  |  | \| \| |  | \| \| |  |  |  |  |  |  |
| 2 | Vitvi07g00046\_t001 |  |  |  | Vitvi14g00477\_t001 |  | Vitvi05g00624\_t001 |  |  |  |  |  |  |
| 2 | Vitvi07g00047\_t001 |  |  |  | \| \| |  | \| \| |  |  |  |  |  |  |
| 2 | Vitvi07g00048\_t001 |  |  |  | \| \| |  | \| \| |  |  |  |  |  |  |
| 2 | Vitvi07g00049\_t001 |  |  |  | Vitvi14g00473\_t001 |  | \| \| |  |  |  |  |  |  |
| 2 | Vitvi07g00050\_t001 |  |  |  | \| \| |  | \| \| |  |  |  |  |  |  |
| 2 | Vitvi07g00052\_t001 |  |  |  | \| \| |  | \| \| |  |  |  |  |  |  |
| 2 | Vitvi07g00053\_t001 |  |  |  | \| \| |  | \| \| |  |  |  |  |  |  |
| 2 | Vitvi07g00054\_t001 |  |  |  | \| \| |  | Vitvi05g00616\_t001 |  |  |  |  |  |  |
| 2 | Vitvi07g00055\_t001 |  |  |  | \| \| |  | \| \| |  |  |  |  |  |  |
| 2 | Vitvi07g04016\_t001 |  |  |  | \| \| |  | \| \| |  |  |  |  |  |  |
| 2 | Vitvi07g04017\_t001 |  |  |  | \| \| |  | \| \| |  |  |  |  |  |  |
| 2 | Vitvi07g04018\_t001 |  |  |  | \| \| |  | \| \| |  |  |  |  |  |  |
| 2 | Vitvi07g00057\_t001 |  |  |  | \| \| |  | \| \| |  |  |  |  |  |  |
| 2 | Vitvi07g02095\_t001 |  |  |  | \| \| |  | \| \| |  |  |  |  |  |  |
| 2 | Vitvi07g02096\_t001 |  |  |  | \| \| |  | \| \| |  |  |  |  |  |  |
| 2 | Vitvi07g00059\_t001 |  |  |  | \| \| |  | \| \| |  |  |  |  |  |  |
| 2 | Vitvi07g00060\_t001 |  |  |  | \| \| |  | \| \| |  |  |  |  |  |  |
| 2 | Vitvi07g00061\_t002 |  |  |  | \| \| |  | Vitvi05g04150\_t001 |  |  |  |  |  |  |
| 2 | Vitvi07g00062\_t001 |  |  |  | \| \| |  | Vitvi05g00612\_t001 |  |  |  |  |  |  |
| 2 | Vitvi07g02097\_t001 |  |  |  | \| \| |  | \| \| |  |  |  |  |  |  |
| 2 | Vitvi07g00063\_t001 |  |  |  | \| \| |  | \| \| |  |  |  |  |  |  |
| 2 | Vitvi07g00064\_t001 |  |  |  | \| \| |  | \| \| |  |  |  |  |  |  |
| 2 | Vitvi07g00065\_t001 |  |  |  | Vitvi14g00469\_t001 |  | \| \| |  |  |  |  |  |  |
| 2 | Vitvi07g00066\_t001 |  |  |  | \| \| |  | Vitvi05g00606\_t001 |  |  |  |  |  |  |
| 2 | Vitvi07g00067\_t001 |  |  |  | \| \| |  | \| \| |  |  |  |  |  |  |
| 2 | Vitvi07g00068\_t001 |  |  |  | \| \| |  | Vitvi05g00605\_t001 |  |  |  |  |  |  |
| 2 | Vitvi07g00069\_t001 |  |  |  | \| \| |  | \| \| |  |  |  |  |  |  |
| 2 | Vitvi07g00071\_t001 |  |  |  | Vitvi14g00458\_t001 |  | Vitvi05g00584\_t001 |  |  |  |  |  |  |
| 1 | Vitvi07g00072\_t001 |  |  |  |  |  | \| \| |  |  |  |  |  |  |
| 1 | Vitvi07g00073\_t002 |  |  |  |  |  | \| \| |  |  |  |  |  |  |
| 1 | Vitvi07g00074\_t001 |  |  |  |  |  | Vitvi05g00568\_t001 |  |  |  |  |  |  |
| 1 | Vitvi07g00075\_t001 |  |  |  |  |  | \| \| |  |  |  |  |  |  |
| 1 | Vitvi07g00077\_t001 |  |  |  |  |  | \| \| |  |  |  |  |  |  |
| 1 | Vitvi07g04019\_t001 |  |  |  |  |  | \| \| |  |  |  |  |  |  |
| 1 | Vitvi07g00078\_t001 |  |  |  |  |  | Vitvi05g00561\_t001 |  |  |  |  |  |  |
| 1 | Vitvi07g00079\_t001 |  |  |  |  |  | \| \| |  |  |  |  |  |  |
| 1 | Vitvi07g00080\_t001 |  |  |  |  |  | \| \| |  |  |  |  |  |  |
| 1 | Vitvi07g00081\_t001 |  |  |  |  |  | \| \| |  |  |  |  |  |  |
| 1 | Vitvi07g00082\_t001 |  |  |  |  |  | Vitvi05g00558\_t002 |  |  |  |  |  |  |
| 1 | Vitvi07g02102\_t001 |  |  |  |  |  | \| \| |  |  |  |  |  |  |
| 1 | Vitvi07g02103\_t001 |  |  |  |  |  | \| \| |  |  |  |  |  |  |
| 1 | Vitvi07g04020\_t001 |  |  |  |  |  | \| \| |  |  |  |  |  |  |
| 1 | Vitvi07g04021\_t001 |  |  |  |  |  | \| \| |  |  |  |  |  |  |
| 1 | Vitvi07g04022\_t001 |  |  |  |  |  | \| \| |  |  |  |  |  |  |
| 1 | Vitvi07g02106\_t001 |  |  |  |  |  | \| \| |  |  |  |  |  |  |
| 1 | Vitvi07g02107\_t001 |  |  |  |  |  | \| \| |  |  |  |  |  |  |
| 1 | Vitvi07g02108\_t001 |  |  |  |  |  | \| \| |  |  |  |  |  |  |
| 1 | Vitvi07g00085\_t001 |  |  |  |  |  | \| \| |  |  |  |  |  |  |
| 1 | Vitvi07g04023\_t001 |  |  |  |  |  | \| \| |  |  |  |  |  |  |
| 1 | Vitvi07g04024\_t001 |  |  |  |  |  | \| \| |  |  |  |  |  |  |
| 1 | Vitvi07g02110\_t001 |  |  |  |  |  | \| \| |  |  |  |  |  |  |
| 1 | Vitvi07g04025\_t001 |  |  |  |  |  | \| \| |  |  |  |  |  |  |
| 1 | Vitvi07g02111\_t001 |  |  |  |  |  | \| \| |  |  |  |  |  |  |
| 1 | Vitvi07g04026\_t001 |  |  |  |  |  | \| \| |  |  |  |  |  |  |
| 1 | Vitvi07g02114\_t001 |  |  |  |  |  | Vitvi05g00556\_t001 |  |  |  |  |  |  |
| 1 | Vitvi07g02115\_t001 |  |  |  |  |  | \| \| |  |  |  |  |  |  |
| 1 | Vitvi07g04027\_t001 |  |  |  |  |  | \| \| |  |  |  |  |  |  |
| 1 | Vitvi07g04028\_t001 |  |  |  |  |  | \| \| |  |  |  |  |  |  |
| 1 | Vitvi07g02119\_t001 |  |  |  |  |  | \| \| |  |  |  |  |  |  |
| 1 | Vitvi07g02120\_t001 |  |  |  |  |  | \| \| |  |  |  |  |  |  |
| 1 | Vitvi07g02121\_t001 |  |  |  |  |  | \| \| |  |  |  |  |  |  |
| 1 | Vitvi07g02122\_t001 |  |  |  |  |  | \| \| |  |  |  |  |  |  |
| 1 | Vitvi07g02123\_t001 |  |  |  |  |  | \| \| |  |  |  |  |  |  |
| 1 | Vitvi07g00087\_t001 |  |  |  |  |  | Vitvi05g00555\_t001 |  |  |  |  |  |  |
| 1 | Vitvi07g00088\_t001 |  |  |  |  |  | Vitvi05g00554\_t001 |  |  |  |  |  |  |
| 1 | Vitvi07g04029\_t001 |  |  |  |  |  | \| \| |  |  |  |  |  |  |
| 1 | Vitvi07g00090\_t001 |  |  |  |  |  | \| \| |  |  |  |  |  |  |
| 1 | Vitvi07g04030\_t001 |  |  |  |  |  | \| \| |  |  |  |  |  |  |
| 1 | Vitvi07g04031\_t001 |  |  |  |  |  | \| \| |  |  |  |  |  |  |
| 1 | Vitvi07g00091\_t001 |  |  |  |  |  | Vitvi05g00546\_t001 |  |  |  |  |  |  |
| 1 | Vitvi07g00094\_t001 |  |  |  |  |  | Vitvi05g00542\_t001 |  |  |  |  |  |  |
| 1 | Vitvi07g00095\_t001 |  |  |  |  |  | \| \| |  |  |  |  |  |  |
| 1 | Vitvi07g00096\_t001 |  |  |  |  |  | \| \| |  |  |  |  |  |  |
| 1 | Vitvi07g00097\_t002 |  |  |  |  |  | \| \| |  |  |  |  |  |  |
| 1 | Vitvi07g00099\_t001 |  |  |  |  |  | \| \| |  |  |  |  |  |  |
| 1 | Vitvi07g04032\_t001 |  |  |  |  |  | Vitvi05g00539\_t003 |  |  |  |  |  |  |
| 1 | Vitvi07g00102\_t001 |  |  |  |  |  | \| \| |  |  |  |  |  |  |
| 1 | Vitvi07g00103\_t001 |  |  |  |  |  | \| \| |  |  |  |  |  |  |
| 1 | Vitvi07g00104\_t001 |  |  |  |  |  | \| \| |  |  |  |  |  |  |
| 1 | Vitvi07g00105\_t001 |  |  |  |  |  | Vitvi05g00533\_t001 |  |  |  |  |  |  |
| 1 | Vitvi07g04033\_t001 |  |  |  |  |  | \| \| |  |  |  |  |  |  |
| 1 | Vitvi07g04034\_t001 |  |  |  |  |  | \| \| |  |  |  |  |  |  |
| 1 | Vitvi07g02126\_t001 |  |  |  |  |  | Vitvi05g00532\_t001 |  |  |  |  |  |  |
| 1 | Vitvi07g04035\_t001 |  |  |  |  |  | \| \| |  |  |  |  |  |  |
| 1 | Vitvi07g04036\_t001 |  |  |  |  |  | \| \| |  |  |  |  |  |  |
| 1 | Vitvi07g00106\_t001 |  |  |  |  |  | \| \| |  |  |  |  |  |  |
| 1 | Vitvi07g00107\_t001 |  |  |  |  |  | \| \| |  |  |  |  |  |  |
| 1 | Vitvi07g02127\_t002 |  |  |  |  |  | \| \| |  |  |  |  |  |  |
| 1 | Vitvi07g04037\_t001 |  |  |  |  |  | Vitvi05g04126\_t001 |  |  |  |  |  |  |
| 1 | Vitvi07g00109\_t001 |  |  |  |  |  | \| \| |  |  |  |  |  |  |
| 1 | Vitvi07g00110\_t001 |  |  |  |  |  | Vitvi05g00527\_t002 |  |  |  |  |  |  |
| 1 | Vitvi07g02129\_t001 |  |  |  |  |  | \| \| |  |  |  |  |  |  |
| 1 | Vitvi07g02130\_t001 |  |  |  |  |  | \| \| |  |  |  |  |  |  |
| 1 | Vitvi07g02131\_t001 |  |  |  |  |  | \| \| |  |  |  |  |  |  |
| 1 | Vitvi07g02132\_t001 |  |  |  |  |  | \| \| |  |  |  |  |  |  |
| 1 | Vitvi07g04038\_t001 |  |  |  |  |  | \| \| |  |  |  |  |  |  |
| 1 | Vitvi07g00112\_t001 |  |  |  |  |  | \| \| |  |  |  |  |  |  |
| 1 | Vitvi07g00113\_t001 |  |  |  |  |  | \| \| |  |  |  |  |  |  |
| 1 | Vitvi07g00114\_t001 |  |  |  |  |  | Vitvi05g04122\_t001 |  |  |  |  |  |  |
| 1 | Vitvi07g00115\_t001 |  |  |  |  |  | \| \| |  |  |  |  |  |  |
| 1 | Vitvi07g04039\_t001 |  |  |  |  |  | \| \| |  |  |  |  |  |  |
| 1 | Vitvi07g00116\_t001 |  |  |  |  |  | Vitvi05g00517\_t001 |  |  |  |  |  |  |
| 1 | Vitvi07g00117\_t001 |  |  |  |  |  | Vitvi05g00510\_t001 |  |  |  |  |  |  |
| 1 | Vitvi07g04040\_t001 |  |  |  |  |  | Vitvi05g00509\_t001 |  |  |  |  |  |  |
| 1 | Vitvi07g04041\_t001 |  |  |  |  |  | Vitvi05g04117\_t001 |  |  |  |  |  |  |
| 1 | Vitvi07g00120\_t001 |  |  |  |  |  | Vitvi05g00496\_t001 |  |  |  |  |  |  |
| 1 | Vitvi07g00121\_t001 |  |  |  |  |  | \| \| |  |  |  |  |  |  |
| 1 | Vitvi07g00122\_t001 |  |  |  |  |  | \| \| |  |  |  |  |  |  |
| 1 | Vitvi07g00123\_t001 |  |  |  |  |  | Vitvi05g01885\_t001 |  |  |  |  |  |  |
| 1 | Vitvi07g00124\_t001 |  |  |  |  |  | \| \| |  |  |  |  |  |  |
| 1 | Vitvi07g02133\_t001 |  |  |  |  |  | \| \| |  |  |  |  |  |  |
| 1 | Vitvi07g02134\_t001 |  |  |  |  |  | \| \| |  |  |  |  |  |  |
| 1 | Vitvi07g00125\_t001 |  |  |  |  |  | Vitvi05g00474\_t001 |  |  |  |  |  |  |
| 1 | Vitvi07g02135\_t001 |  |  |  |  |  | \| \| |  |  |  |  |  |  |
| 1 | Vitvi07g00126\_t001 |  |  |  |  |  | \| \| |  |  |  |  |  |  |
| 1 | Vitvi07g00127\_t001 |  |  |  |  |  | \| \| |  |  |  |  |  |  |
| 1 | Vitvi07g00128\_t001 |  |  |  |  |  | \| \| |  |  |  |  |  |  |
| 1 | Vitvi07g00129\_t001 |  |  |  |  |  | \| \| |  |  |  |  |  |  |
| 1 | Vitvi07g00130\_t001 |  |  |  |  |  | \| \| |  |  |  |  |  |  |
| 1 | Vitvi07g04042\_t001 |  |  |  |  |  | \| \| |  |  |  |  |  |  |
| 1 | Vitvi07g00131\_t001 |  |  |  |  |  | Vitvi05g00469\_t001 |  |  |  |  |  |  |
| 1 | Vitvi07g04043\_t001 |  |  |  |  |  | \| \| |  |  |  |  |  |  |
| 1 | Vitvi07g00132\_t001 |  |  |  |  |  | \| \| |  |  |  |  |  |  |
| 1 | Vitvi07g02136\_t001 |  |  |  |  |  | Vitvi05g00467\_t001 |  |  |  |  |  |  |
| 1 | Vitvi07g02137\_t001 |  |  |  |  |  | \| \| |  |  |  |  |  |  |
| 1 | Vitvi07g00134\_t001 |  |  |  |  |  | Vitvi05g00458\_t002 |  |  |  |  |  |  |
| 1 | Vitvi07g00135\_t001 |  |  |  |  |  | \| \| |  |  |  |  |  |  |
| 1 | Vitvi07g00136\_t001 |  |  |  |  |  | \| \| |  |  |  |  |  |  |
| 1 | Vitvi07g00137\_t001 |  |  |  |  |  | \| \| |  |  |  |  |  |  |
| 1 | Vitvi07g00138\_t001.1.6037826f |  |  |  |  |  | \| \| |  |  |  |  |  |  |
| 1 | Vitvi07g00139\_t001 |  |  |  |  |  | \| \| |  |  |  |  |  |  |
| 1 | Vitvi07g00140\_t001 |  |  |  |  |  | \| \| |  |  |  |  |  |  |
| 1 | Vitvi07g00141\_t001 |  |  |  |  |  | \| \| |  |  |  |  |  |  |
| 1 | Vitvi07g00142\_t001 |  |  |  |  |  | Vitvi05g00437\_t001 |  |  |  |  |  |  |
| 1 | Vitvi07g04044\_t001 |  |  |  |  |  | \| \| |  |  |  |  |  |  |
| 1 | Vitvi07g00144\_t001 |  |  |  |  |  | \| \| |  |  |  |  |  |  |
| 1 | Vitvi07g02138\_t001 |  |  |  |  |  | \| \| |  |  |  |  |  |  |
| 1 | Vitvi07g00145\_t002 |  |  |  |  |  | \| \| |  |  |  |  |  |  |
| 1 | Vitvi07g00146\_t001 |  |  |  |  |  | Vitvi05g00430\_t001 |  |  |  |  |  |  |
| 1 | Vitvi07g00147\_t001 |  |  |  |  |  | \| \| |  |  |  |  |  |  |
| 1 | Vitvi07g02140\_t002 |  |  |  |  |  | \| \| |  |  |  |  |  |  |
| 1 | Vitvi07g02141\_t001 |  |  |  |  |  | \| \| |  |  |  |  |  |  |
| 1 | Vitvi07g00149\_t001 |  |  |  |  |  | \| \| |  |  |  |  |  |  |
| 1 | Vitvi07g00150\_t002 |  |  |  |  |  | \| \| |  |  |  |  |  |  |
| 1 | Vitvi07g00151\_t001 |  |  |  |  |  | \| \| |  |  |  |  |  |  |
| 1 | Vitvi07g00152\_t001 |  |  |  |  |  | Vitvi05g01875\_t001 |  |  |  |  |  |  |
| 1 | Vitvi07g00154\_t001 |  |  |  |  |  | Vitvi05g00421\_t001 |  |  |  |  |  |  |
| 1 | Vitvi07g00155\_t001 |  |  |  |  |  | \| \| |  |  |  |  |  |  |
| 1 | Vitvi07g00157\_t001 |  |  |  |  |  | \| \| |  |  |  |  |  |  |
| 1 | Vitvi07g00158\_t001 |  |  |  |  |  | Vitvi05g00420\_t001 |  |  |  |  |  |  |
| 1 | Vitvi07g00159\_t005 |  |  |  |  |  | \| \| |  |  |  |  |  |  |
| 1 | Vitvi07g00160\_t001 |  |  |  |  |  | \| \| |  |  |  |  |  |  |
| 1 | Vitvi07g00162\_t001 |  |  |  |  |  | \| \| |  |  |  |  |  |  |
| 1 | Vitvi07g04045\_t001 |  |  |  |  |  | \| \| |  |  |  |  |  |  |
| 1 | Vitvi07g00164\_t001 |  |  |  |  |  | \| \| |  |  |  |  |  |  |
| 1 | Vitvi07g00166\_t001 |  |  |  |  |  | \| \| |  |  |  |  |  |  |
| 1 | Vitvi07g00167\_t001 |  |  |  |  |  | \| \| |  |  |  |  |  |  |
| 1 | Vitvi07g00168\_t001 |  |  |  |  |  | \| \| |  |  |  |  |  |  |
| 1 | Vitvi07g00169\_t001 |  |  |  |  |  | Vitvi05g00413\_t001 |  |  |  |  |  |  |
| 1 | Vitvi07g00171\_t003 |  |  |  |  |  | \| \| |  |  |  |  |  |  |
| 1 | Vitvi07g02143\_t001 |  |  |  |  |  | Vitvi05g00410\_t001 |  |  |  |  |  |  |
| 1 | Vitvi07g02144\_t002 |  |  |  |  |  | \| \| |  |  |  |  |  |  |
| 1 | Vitvi07g00172\_t001 |  |  |  |  |  | \| \| |  |  |  |  |  |  |
| 1 | Vitvi07g02146\_t001 |  |  |  |  |  | \| \| |  |  |  |  |  |  |
| 1 | Vitvi07g00173\_t003 |  |  |  |  |  | \| \| |  |  |  |  |  |  |
| 1 | Vitvi07g00174\_t001 |  |  |  |  |  | \| \| |  |  |  |  |  |  |
| 1 | Vitvi07g00175\_t001 |  |  |  |  |  | \| \| |  |  |  |  |  |  |
| 1 | Vitvi07g00176\_t001 |  |  |  |  |  | \| \| |  |  |  |  |  |  |
| 1 | Vitvi07g00177\_t001 |  |  |  |  |  | \| \| |  |  |  |  |  |  |
| 1 | Vitvi07g00178\_t001 |  |  |  |  |  | \| \| |  |  |  |  |  |  |
| 1 | Vitvi07g00179\_t001.1.6037826e |  |  |  |  |  | \| \| |  |  |  |  |  |  |
| 1 | Vitvi07g00181\_t001 |  |  |  |  |  | \| \| |  |  |  |  |  |  |
| 1 | Vitvi07g00182\_t001 |  |  |  |  |  | \| \| |  |  |  |  |  |  |
| 1 | Vitvi07g04046\_t001 |  |  |  |  |  | \| \| |  |  |  |  |  |  |
| 1 | Vitvi07g00184\_t001 |  |  |  |  |  | Vitvi05g00408\_t001 |  |  |  |  |  |  |
| 1 | Vitvi07g02147\_t001 |  |  |  |  |  | \| \| |  |  |  |  |  |  |
| 1 | Vitvi07g00185\_t001 |  |  |  |  |  | \| \| |  |  |  |  |  |  |
| 1 | Vitvi07g00186\_t001 |  |  |  |  |  | \| \| |  |  |  |  |  |  |
| 1 | Vitvi07g00187\_t001 |  |  |  |  |  | Vitvi05g00403\_t001 |  |  |  |  |  |  |
| 1 | Vitvi07g00188\_t001 |  |  |  |  |  | \| \| |  |  |  |  |  |  |
| 1 | Vitvi07g00189\_t001 |  |  |  |  |  | \| \| |  |  |  |  |  |  |
| 1 | Vitvi07g02148\_t001 |  |  |  |  |  | \| \| |  |  |  |  |  |  |
| 1 | Vitvi07g02149\_t001 |  |  |  |  |  | \| \| |  |  |  |  |  |  |
| 1 | Vitvi07g00190\_t001 |  |  |  |  |  | \| \| |  |  |  |  |  |  |
| 1 | Vitvi07g00191\_t001 |  |  |  |  |  | Vitvi05g00398\_t001 |  |  |  |  |  |  |
| 1 | Vitvi07g04047\_t001 |  |  |  |  |  | \| \| |  |  |  |  |  |  |
| 1 | Vitvi07g00193\_t001 |  |  |  |  |  | Vitvi05g00394\_t001 |  |  |  |  |  |  |
| 1 | Vitvi07g00194\_t001 |  |  |  |  |  | \| \| |  |  |  |  |  |  |
| 1 | Vitvi07g00195\_t001 |  |  |  |  |  | \| \| |  |  |  |  |  |  |
| 1 | Vitvi07g00196\_t001 |  |  |  |  |  | \| \| |  |  |  |  |  |  |
| 1 | Vitvi07g00197\_t001 |  |  |  |  |  | \| \| |  |  |  |  |  |  |
| 1 | Vitvi07g00198\_t001 |  |  |  |  |  | \| \| |  |  |  |  |  |  |
| 2 | Vitvi07g00199\_t001 |  | Vitvi14g00301\_t001 |  |  |  | \| \| |  |  |  |  |  |  |
| 2 | Vitvi07g04048\_t001 |  | \| \| |  |  |  | \| \| |  |  |  |  |  |  |
| 2 | Vitvi07g00200\_t001 |  | \| \| |  |  |  | \| \| |  |  |  |  |  |  |
| 2 | Vitvi07g04049\_t001 |  | \| \| |  |  |  | \| \| |  |  |  |  |  |  |
| 2 | Vitvi07g00201\_t001 |  | \| \| |  |  |  | \| \| |  |  |  |  |  |  |
| 2 | Vitvi07g00202\_t001 |  | \| \| |  |  |  | Vitvi05g00386\_t001 |  |  |  |  |  |  |
| 2 | Vitvi07g00203\_t001 |  | \| \| |  |  |  | \| \| |  |  |  |  |  |  |
| 2 | Vitvi07g00204\_t002 |  | \| \| |  |  |  | \| \| |  |  |  |  |  |  |
| 2 | Vitvi07g00205\_t001 |  | \| \| |  |  |  | \| \| |  |  |  |  |  |  |
| 2 | Vitvi07g00207\_t001 |  | Vitvi14g04108\_t001 |  |  |  | Vitvi05g01869\_t001 |  |  |  |  |  |  |
| 2 | Vitvi07g00208\_t001 |  | Vitvi14g00315\_t001 |  |  |  | \| \| |  |  |  |  |  |  |
| 2 | Vitvi07g00209\_t001 |  | Vitvi14g00316\_t001 |  |  |  | Vitvi05g00384\_t001 |  |  |  |  |  |  |
| 2 | Vitvi07g00210\_t001 |  | Vitvi14g00317\_t001 |  |  |  | \| \| |  |  |  |  |  |  |
| 2 | Vitvi07g00211\_t001 |  | Vitvi14g00318\_t001 |  |  |  | Vitvi05g00382\_t001 |  |  |  |  |  |  |
| 2 | Vitvi07g00213\_t001 |  | \| \| |  |  |  | \| \| |  |  |  |  |  |  |
| 2 | Vitvi07g00214\_t002 |  | \| \| |  |  |  | \| \| |  |  |  |  |  |  |
| 2 | Vitvi07g00216\_t001 |  | \| \| |  |  |  | \| \| |  |  |  |  |  |  |
| 2 | Vitvi07g00217\_t001 |  | Vitvi14g00322\_t001 |  |  |  | \| \| |  |  |  |  |  |  |
| 2 | Vitvi07g00218\_t001 |  | \| \| |  |  |  | \| \| |  |  |  |  |  |  |
| 2 | Vitvi07g00219\_t001 |  | Vitvi14g00323\_t001 |  |  |  | \| \| |  |  |  |  |  |  |
| 1 | Vitvi07g00220\_t001 |  |  |  |  |  | \| \| |  |  |  |  |  |  |
| 1 | Vitvi07g02153\_t001 |  |  |  |  |  | \| \| |  |  |  |  |  |  |
| 1 | Vitvi07g00221\_t001 |  |  |  |  |  | \| \| |  |  |  |  |  |  |
| 1 | Vitvi07g04050\_t001 |  |  |  |  |  | \| \| |  |  |  |  |  |  |
| 1 | Vitvi07g00223\_t001 |  |  |  |  |  | \| \| |  |  |  |  |  |  |
| 1 | Vitvi07g00224\_t001 |  |  |  |  |  | \| \| |  |  |  |  |  |  |
| 1 | Vitvi07g04051\_t001 |  |  |  |  |  | \| \| |  |  |  |  |  |  |
| 1 | Vitvi07g00225\_t001 |  |  |  |  |  | \| \| |  |  |  |  |  |  |
| 1 | Vitvi07g04052\_t001 |  |  |  |  |  | \| \| |  |  |  |  |  |  |
| 1 | Vitvi07g00226\_t001 |  |  |  |  |  | \| \| |  |  |  |  |  |  |
| 1 | Vitvi07g00227\_t001 |  |  |  |  |  | \| \| |  |  |  |  |  |  |
| 1 | Vitvi07g00228\_t001 |  |  |  |  |  | \| \| |  |  |  |  |  |  |
| 1 | Vitvi07g00230\_t001 |  |  |  |  |  | Vitvi05g00370\_t001 |  |  |  |  |  |  |
| 1 | Vitvi07g00231\_t001 |  |  |  |  |  | \| \| |  |  |  |  |  |  |
| 1 | Vitvi07g02156\_t001 |  |  |  |  |  | \| \| |  |  |  |  |  |  |
| 1 | Vitvi07g04053\_t001 |  |  |  |  |  | \| \| |  |  |  |  |  |  |
| 1 | Vitvi07g00232\_t001 |  |  |  |  |  | \| \| |  |  |  |  |  |  |
| 1 | Vitvi07g00233\_t001 |  |  |  |  |  | \| \| |  |  |  |  |  |  |
| 1 | Vitvi07g00234\_t001 |  |  |  |  |  | \| \| |  |  |  |  |  |  |
| 1 | Vitvi07g04054\_t001 |  |  |  |  |  | \| \| |  |  |  |  |  |  |
| 1 | Vitvi07g00236\_t001 |  |  |  |  |  | \| \| |  |  |  |  |  |  |
| 1 | Vitvi07g00237\_t001 |  |  |  |  |  | \| \| |  |  |  |  |  |  |
| 1 | Vitvi07g00238\_t001 |  |  |  |  |  | \| \| |  |  |  |  |  |  |
| 1 | Vitvi07g04055\_t001 |  |  |  |  |  | \| \| |  |  |  |  |  |  |
| 1 | Vitvi07g00240\_t001 |  |  |  |  |  | \| \| |  |  |  |  |  |  |
| 1 | Vitvi07g00241\_t001 |  |  |  |  |  | \| \| |  |  |  |  |  |  |
| 1 | Vitvi07g00242\_t001 |  |  |  |  |  | \| \| |  |  |  |  |  |  |
| 1 | Vitvi07g02158\_t001 |  |  |  |  |  | \| \| |  |  |  |  |  |  |
| 1 | Vitvi07g00243\_t001 |  |  |  |  |  | \| \| |  |  |  |  |  |  |
| 1 | Vitvi07g00244\_t001 |  |  |  |  |  | Vitvi05g00359\_t001 |  |  |  |  |  |  |
| 1 | Vitvi07g04056\_t001 |  |  |  |  |  | \| \| |  |  |  |  |  |  |
| 1 | Vitvi07g02159\_t002 |  |  |  |  |  | Vitvi05g01862\_t002 |  |  |  |  |  |  |
| 1 | Vitvi07g02160\_t001 |  |  |  |  |  | \| \| |  |  |  |  |  |  |
| 1 | Vitvi07g00248\_t002 |  |  |  |  |  | \| \| |  |  |  |  |  |  |
| 1 | Vitvi07g02161\_t001 |  |  |  |  |  | \| \| |  |  |  |  |  |  |
| 1 | Vitvi07g00250\_t001 |  |  |  |  |  | \| \| |  |  |  |  |  |  |
| 1 | Vitvi07g00251\_t001 |  |  |  |  |  | Vitvi05g00349\_t001 |  |  |  |  |  |  |
| 1 | Vitvi07g00252\_t001 |  |  |  |  |  | \| \| |  |  |  |  |  |  |
| 1 | Vitvi07g00253\_t001 |  |  |  |  |  | Vitvi05g00347\_t004 |  |  |  |  |  |  |
| 1 | Vitvi07g02162\_t001 |  |  |  |  |  | \| \| |  |  |  |  |  |  |
| 1 | Vitvi07g02163\_t001 |  |  |  |  |  | \| \| |  |  |  |  |  |  |
| 1 | Vitvi07g00254\_t001 |  |  |  |  |  | \| \| |  |  |  |  |  |  |
| 1 | Vitvi07g04057\_t001 |  |  |  |  |  | \| \| |  |  |  |  |  |  |
| 1 | Vitvi07g00255\_t001 |  |  |  |  |  | Vitvi05g00342\_t001 |  |  |  |  |  |  |
| 1 | Vitvi07g00256\_t001 |  |  |  |  |  | \| \| |  |  |  |  |  |  |
| 1 | Vitvi07g00257\_t002 |  |  |  |  |  | \| \| |  |  |  |  |  |  |
| 1 | Vitvi07g00259\_t001 |  |  |  |  |  | \| \| |  |  |  |  |  |  |
| 1 | Vitvi07g02164\_t001 |  |  |  |  |  | \| \| |  |  |  |  |  |  |
| 1 | Vitvi07g00260\_t001 |  |  |  |  |  | \| \| |  |  |  |  |  |  |
| 1 | Vitvi07g04058\_t001 |  |  |  |  |  | \| \| |  |  |  |  |  |  |
| 1 | Vitvi07g00262\_t002 |  |  |  |  |  | Vitvi05g00333\_t001 |  |  |  |  |  |  |
| 1 | Vitvi07g04059\_t001 |  |  |  |  |  | \| \| |  |  |  |  |  |  |
| 1 | Vitvi07g00264\_t001 |  |  |  |  |  | \| \| |  |  |  |  |  |  |
| 1 | Vitvi07g00265\_t001 |  |  |  |  |  | \| \| |  |  |  |  |  |  |
| 1 | Vitvi07g02165\_t001 |  |  |  |  |  | \| \| |  |  |  |  |  |  |
| 1 | Vitvi07g02166\_t001 |  |  |  |  |  | \| \| |  |  |  |  |  |  |
| 1 | Vitvi07g02167\_t001 |  |  |  |  |  | \| \| |  |  |  |  |  |  |
| 1 | Vitvi07g04060\_t001 |  |  |  |  |  | \| \| |  |  |  |  |  |  |
| 1 | Vitvi07g04061\_t001 |  |  |  |  |  | \| \| |  |  |  |  |  |  |
| 1 | Vitvi07g04062\_t001 |  |  |  |  |  | \| \| |  |  |  |  |  |  |
| 1 | Vitvi07g04063\_t001 |  |  |  |  |  | \| \| |  |  |  |  |  |  |
| 1 | Vitvi07g04064\_t001 |  |  |  |  |  | \| \| |  |  |  |  |  |  |
| 1 | Vitvi07g02173\_t001 |  |  |  |  |  | \| \| |  |  |  |  |  |  |
| 1 | Vitvi07g02174\_t001 |  |  |  |  |  | \| \| |  |  |  |  |  |  |
| 1 | Vitvi07g00266\_t001 |  |  |  |  |  | \| \| |  |  |  |  |  |  |
| 1 | Vitvi07g00267\_t001 |  |  |  |  |  | \| \| |  |  |  |  |  |  |
| 2 | Vitvi07g00268\_t001 |  | Vitvi05g00296\_t001 |  |  |  | \| \| |  |  |  |  |  |  |
| 2 | Vitvi07g00269\_t001 |  | \| \| |  |  |  | Vitvi05g00326\_t001 |  |  |  |  |  |  |
| 2 | Vitvi07g00270\_t001 |  | \| \| |  |  |  | \| \| |  |  |  |  |  |  |
| 2 | Vitvi07g00271\_t001 |  | \| \| |  |  |  | \| \| |  |  |  |  |  |  |
| 2 | Vitvi07g00272\_t001 |  | Vitvi05g00301\_t001 |  |  |  | Vitvi05g00301\_t001 |  |  |  |  |  |  |
| 2 | Vitvi07g04065\_t001 |  | \| \| |  |  |  | \| \| |  |  |  |  |  |  |
| 2 | Vitvi07g00273\_t001 |  | \| \| |  |  |  | Vitvi05g00299\_t001 |  |  |  |  |  |  |
| 1 | Vitvi07g04066\_t001 |  | \| \| |  |  |  |  |  |  |  |  |  |  |
| 1 | Vitvi07g00274\_t001 |  | \| \| |  |  |  |  |  |  |  |  |  |  |
| 1 | Vitvi07g04067\_t001 |  | \| \| |  |  |  |  |  |  |  |  |  |  |
| 1 | Vitvi07g02178\_t001 |  | \| \| |  |  |  |  |  |  |  |  |  |  |
| 1 | Vitvi07g00275\_t001 |  | \| \| |  |  |  |  |  |  |  |  |  |  |
| 1 | Vitvi07g04068\_t001 |  | \| \| |  |  |  |  |  |  |  |  |  |  |
| 1 | Vitvi07g04069\_t001 |  | \| \| |  |  |  |  |  |  |  |  |  |  |
| 1 | Vitvi07g04070\_t001 |  | \| \| |  |  |  |  |  |  |  |  |  |  |
| 1 | Vitvi07g04071\_t001 |  | \| \| |  |  |  |  |  |  |  |  |  |  |
| 1 | Vitvi07g02181\_t001 |  | \| \| |  |  |  |  |  |  |  |  |  |  |
| 1 | Vitvi07g02182\_t001 |  | \| \| |  |  |  |  |  |  |  |  |  |  |
| 1 | Vitvi07g02183\_t001 |  | \| \| |  |  |  |  |  |  |  |  |  |  |
| 1 | Vitvi07g00276\_t001 |  | \| \| |  |  |  |  |  |  |  |  |  |  |
| 1 | Vitvi07g00277\_t001 |  | \| \| |  |  |  |  |  |  |  |  |  |  |
| 1 | Vitvi07g00278\_t001 |  | Vitvi05g00309\_t001 |  |  |  |  |  |  |  |  |  |  |
| 1 | Vitvi07g02184\_t001 |  | \| \| |  |  |  |  |  |  |  |  |  |  |
| 1 | Vitvi07g02185\_t001 |  | \| \| |  |  |  |  |  |  |  |  |  |  |
| 1 | Vitvi07g00279\_t001 |  | \| \| |  |  |  |  |  |  |  |  |  |  |
| 1 | Vitvi07g00280\_t001 |  | \| \| |  |  |  |  |  |  |  |  |  |  |
| 1 | Vitvi07g04072\_t001 |  | \| \| |  |  |  |  |  |  |  |  |  |  |
| 1 | Vitvi07g00282\_t001 |  | \| \| |  |  |  |  |  |  |  |  |  |  |
| 1 | Vitvi07g02188\_t003 |  | \| \| |  |  |  |  |  |  |  |  |  |  |
| 1 | Vitvi07g02190\_t001 |  | \| \| |  |  |  |  |  |  |  |  |  |  |
| 1 | Vitvi07g00283\_t001 |  | \| \| |  |  |  |  |  |  |  |  |  |  |
| 1 | Vitvi07g00284\_t001 |  | \| \| |  |  |  |  |  |  |  |  |  |  |
| 1 | Vitvi07g00285\_t001 |  | \| \| |  |  |  |  |  |  |  |  |  |  |
| 1 | Vitvi07g02191\_t001 |  | \| \| |  |  |  |  |  |  |  |  |  |  |
| 1 | Vitvi07g00286\_t001 |  | \| \| |  |  |  |  |  |  |  |  |  |  |
| 1 | Vitvi07g00287\_t001 |  | \| \| |  |  |  |  |  |  |  |  |  |  |
| 1 | Vitvi07g00289\_t001 |  | \| \| |  |  |  |  |  |  |  |  |  |  |
| 1 | Vitvi07g00290\_t001 |  | Vitvi05g00313\_t001 |  |  |  |  |  |  |  |  |  |  |
| 1 | Vitvi07g00291\_t001 |  | \| \| |  |  |  |  |  |  |  |  |  |  |
| 1 | Vitvi07g02193\_t001 |  | \| \| |  |  |  |  |  |  |  |  |  |  |
| 1 | Vitvi07g00292\_t001 |  | \| \| |  |  |  |  |  |  |  |  |  |  |
| 1 | Vitvi07g00293\_t001 |  | \| \| |  |  |  |  |  |  |  |  |  |  |
| 1 | Vitvi07g00294\_t001 |  | \| \| |  |  |  |  |  |  |  |  |  |  |
| 1 | Vitvi07g00295\_t001 |  | \| \| |  |  |  |  |  |  |  |  |  |  |
| 1 | Vitvi07g02194\_t001 |  | \| \| |  |  |  |  |  |  |  |  |  |  |
| 1 | Vitvi07g00296\_t001 |  | \| \| |  |  |  |  |  |  |  |  |  |  |
| 1 | Vitvi07g00298\_t001 |  | Vitvi05g00319\_t001 |  |  |  |  |  |  |  |  |  |  |
| 1 | Vitvi07g00299\_t001 |  | Vitvi05g00321\_t001 |  |  |  |  |  |  |  |  |  |  |
| 0 | Vitvi07g00300\_t001 |  |  |  |  |  |  |  |  |  |  |  |  |
| 0 | Vitvi07g00301\_t001 |  |  |  |  |  |  |  |  |  |  |  |  |
| 0 | Vitvi07g00302\_t001 |  |  |  |  |  |  |  |  |  |  |  |  |
| 0 | Vitvi07g04073\_t001 |  |  |  |  |  |  |  |  |  |  |  |  |
| 0 | Vitvi07g00303\_t001 |  |  |  |  |  |  |  |  |  |  |  |  |
| 0 | Vitvi07g00306\_t002 |  |  |  |  |  |  |  |  |  |  |  |  |
| 0 | Vitvi07g00307\_t001 |  |  |  |  |  |  |  |  |  |  |  |  |
| 0 | Vitvi07g00308\_t002 |  |  |  |  |  |  |  |  |  |  |  |  |
| 0 | Vitvi07g00309\_t001 |  |  |  |  |  |  |  |  |  |  |  |  |
| 0 | Vitvi07g00310\_t003 |  |  |  |  |  |  |  |  |  |  |  |  |
| 0 | Vitvi07g04074\_t001 |  |  |  |  |  |  |  |  |  |  |  |  |
| 0 | Vitvi07g00311\_t001 |  |  |  |  |  |  |  |  |  |  |  |  |
| 0 | Vitvi07g00312\_t001 |  |  |  |  |  |  |  |  |  |  |  |  |
| 0 | Vitvi07g00313\_t001 |  |  |  |  |  |  |  |  |  |  |  |  |
| 0 | Vitvi07g00314\_t001 |  |  |  |  |  |  |  |  |  |  |  |  |
| 0 | Vitvi07g04075\_t001 |  |  |  |  |  |  |  |  |  |  |  |  |
| 0 | Vitvi07g00315\_t001 |  |  |  |  |  |  |  |  |  |  |  |  |
| 1 | Vitvi07g00316\_t001 |  | Vitvi14g02509\_t001 |  |  |  |  |  |  |  |  |  |  |
| 1 | Vitvi07g00317\_t001 |  | \| \| |  |  |  |  |  |  |  |  |  |  |
| 1 | Vitvi07g02198\_t001 |  | \| \| |  |  |  |  |  |  |  |  |  |  |
| 1 | Vitvi07g02201\_t002 |  | \| \| |  |  |  |  |  |  |  |  |  |  |
| 1 | Vitvi07g04076\_t001 |  | \| \| |  |  |  |  |  |  |  |  |  |  |
| 1 | Vitvi07g04077\_t001 |  | \| \| |  |  |  |  |  |  |  |  |  |  |
| 1 | Vitvi07g04078\_t001 |  | \| \| |  |  |  |  |  |  |  |  |  |  |
| 1 | Vitvi07g00320\_t003 |  | \| \| |  |  |  |  |  |  |  |  |  |  |
| 1 | Vitvi07g02202\_t001 |  | Vitvi14g00180\_t003 |  |  |  |  |  |  |  |  |  |  |
| 1 | Vitvi07g04079\_t001 |  | \| \| |  |  |  |  |  |  |  |  |  |  |
| 1 | Vitvi07g04080\_t001 |  | \| \| |  |  |  |  |  |  |  |  |  |  |
| 1 | Vitvi07g04081\_t001 |  | \| \| |  |  |  |  |  |  |  |  |  |  |
| 1 | Vitvi07g00321\_t001 |  | \| \| |  |  |  |  |  |  |  |  |  |  |
| 1 | Vitvi07g02203\_t001 |  | \| \| |  |  |  |  |  |  |  |  |  |  |
| 1 | Vitvi07g00322\_t001 |  | \| \| |  |  |  |  |  |  |  |  |  |  |
| 1 | Vitvi07g00323\_t001 |  | \| \| |  |  |  |  |  |  |  |  |  |  |
| 1 | Vitvi07g00324\_t001 |  | \| \| |  |  |  |  |  |  |  |  |  |  |
| 2 | Vitvi07g00325\_t001 |  | \| \| |  | Vitvi05g00009\_t001 |  |  |  |  |  |  |  |  |
| 2 | Vitvi07g04082\_t001 |  | \| \| |  | \| \| |  |  |  |  |  |  |  |  |
| 2 | Vitvi07g00327\_t001 |  | \| \| |  | \| \| |  |  |  |  |  |  |  |  |
| 2 | Vitvi07g04083\_t001 |  | \| \| |  | \| \| |  |  |  |  |  |  |  |  |
| 2 | Vitvi07g00328\_t001 |  | \| \| |  | \| \| |  |  |  |  |  |  |  |  |
| 2 | Vitvi07g02204\_t001 |  | \| \| |  | \| \| |  |  |  |  |  |  |  |  |
| 2 | Vitvi07g00329\_t001 |  | \| \| |  | \| \| |  |  |  |  |  |  |  |  |
| 2 | Vitvi07g04084\_t001 |  | \| \| |  | \| \| |  |  |  |  |  |  |  |  |
| 2 | Vitvi07g00330\_t001 |  | Vitvi14g00159\_t001 |  | Vitvi05g00010\_t001 |  |  |  |  |  |  |  |  |
| 2 | Vitvi07g00331\_t001 |  | \| \| |  | \| \| |  |  |  |  |  |  |  |  |
| 2 | Vitvi07g00332\_t001 |  | \| \| |  | \| \| |  |  |  |  |  |  |  |  |
| 2 | Vitvi07g00333\_t001 |  | \| \| |  | \| \| |  |  |  |  |  |  |  |  |
| 2 | Vitvi07g00334\_t001 |  | \| \| |  | \| \| |  |  |  |  |  |  |  |  |
| 2 | Vitvi07g00335\_t001.1.6037826e |  | \| \| |  | \| \| |  |  |  |  |  |  |  |  |
| 2 | Vitvi07g00336\_t001 |  | \| \| |  | \| \| |  |  |  |  |  |  |  |  |
| 2 | Vitvi07g00337\_t001 |  | \| \| |  | \| \| |  |  |  |  |  |  |  |  |
| 2 | Vitvi07g00338\_t001 |  | \| \| |  | \| \| |  |  |  |  |  |  |  |  |
| 2 | Vitvi07g00339\_t001 |  | \| \| |  | \| \| |  |  |  |  |  |  |  |  |
| 2 | Vitvi07g00340\_t001 |  | \| \| |  | \| \| |  |  |  |  |  |  |  |  |
| 2 | Vitvi07g00341\_t001 |  | \| \| |  | \| \| |  |  |  |  |  |  |  |  |
| 2 | Vitvi07g00342\_t001 |  | \| \| |  | \| \| |  |  |  |  |  |  |  |  |
| 2 | Vitvi07g00343\_t001 |  | \| \| |  | \| \| |  |  |  |  |  |  |  |  |
| 2 | Vitvi07g00346\_t001 |  | Vitvi14g00151\_t001 |  | Vitvi05g00033\_t001 |  |  |  |  |  |  |  |  |
| 2 | Vitvi07g00347\_t001 |  | \| \| |  | Vitvi05g01742\_t002 |  |  |  |  |  |  |  |  |
| 2 | Vitvi07g00348\_t001 |  | \| \| |  | Vitvi05g00041\_t001 |  |  |  |  |  |  |  |  |
| 2 | Vitvi07g00349\_t001 |  | \| \| |  | \| \| |  |  |  |  |  |  |  |  |
| 2 | Vitvi07g00350\_t001 |  | \| \| |  | \| \| |  |  |  |  |  |  |  |  |
| 2 | Vitvi07g00351\_t001 |  | \| \| |  | \| \| |  |  |  |  |  |  |  |  |
| 2 | Vitvi07g04085\_t001 |  | \| \| |  | \| \| |  |  |  |  |  |  |  |  |
| 2 | Vitvi07g00352\_t001 |  | \| \| |  | \| \| |  |  |  |  |  |  |  |  |
| 2 | Vitvi07g00353\_t001 |  | \| \| |  | Vitvi05g00045\_t001 |  |  |  |  |  |  |  |  |
| 2 | Vitvi07g00354\_t001 |  | \| \| |  | Vitvi05g00047\_t001 |  |  |  |  |  |  |  |  |
| 2 | Vitvi07g02206\_t001 |  | \| \| |  | \| \| |  |  |  |  |  |  |  |  |
| 2 | Vitvi07g00355\_t001 |  | \| \| |  | \| \| |  |  |  |  |  |  |  |  |
| 2 | Vitvi07g02207\_t001 |  | \| \| |  | \| \| |  |  |  |  |  |  |  |  |
| 2 | Vitvi07g02208\_t001 |  | \| \| |  | \| \| |  |  |  |  |  |  |  |  |
| 2 | Vitvi07g00356\_t001 |  | \| \| |  | \| \| |  |  |  |  |  |  |  |  |
| 2 | Vitvi07g02209\_t001 |  | \| \| |  | \| \| |  |  |  |  |  |  |  |  |
| 2 | Vitvi07g04086\_t001 |  | \| \| |  | \| \| |  |  |  |  |  |  |  |  |
| 2 | Vitvi07g00357\_t001 |  | \| \| |  | Vitvi05g01724\_t001 |  |  |  |  |  |  |  |  |
| 2 | Vitvi07g00358\_t001 |  | \| \| |  | \| \| |  |  |  |  |  |  |  |  |
| 2 | Vitvi07g02210\_t001 |  | \| \| |  | Vitvi05g00053\_t001 |  |  |  |  |  |  |  |  |
| 2 | Vitvi07g00359\_t001 |  | \| \| |  | \| \| |  |  |  |  |  |  |  |  |
| 2 | Vitvi07g04087\_t001 |  | \| \| |  | \| \| |  |  |  |  |  |  |  |  |
| 2 | Vitvi07g00360\_t001 |  | \| \| |  | \| \| |  |  |  |  |  |  |  |  |
| 2 | Vitvi07g00361\_t001 |  | \| \| |  | \| \| |  |  |  |  |  |  |  |  |
| 2 | Vitvi07g00362\_t001 |  | Vitvi14g00138\_t001 |  | \| \| |  |  |  |  |  |  |  |  |
| 2 | Vitvi07g00363\_t001 |  | \| \| |  | Vitvi05g00061\_t001 |  |  |  |  |  |  |  |  |
| 2 | Vitvi07g00364\_t001 |  | \| \| |  | \| \| |  |  |  |  |  |  |  |  |
| 2 | Vitvi07g00365\_t001 |  | \| \| |  | Vitvi05g00065\_t001 |  |  |  |  |  |  |  |  |
| 2 | Vitvi07g00366\_t001 |  | \| \| |  | \| \| |  |  |  |  |  |  |  |  |
| 2 | Vitvi07g00367\_t001 |  | \| \| |  | Vitvi05g01752\_t001 |  |  |  |  |  |  |  |  |
| 2 | Vitvi07g00368\_t001 |  | Vitvi14g00127\_t001 |  | \| \| |  |  |  |  |  |  |  |  |
| 2 | Vitvi07g04088\_t001 |  | \| \| |  | \| \| |  |  |  |  |  |  |  |  |
| 2 | Vitvi07g00369\_t001 |  | \| \| |  | \| \| |  |  |  |  |  |  |  |  |
| 2 | Vitvi07g00370\_t001 |  | \| \| |  | Vitvi05g00073\_t002 |  |  |  |  |  |  |  |  |
| 2 | Vitvi07g00371\_t001 |  | \| \| |  | \| \| |  |  |  |  |  |  |  |  |
| 2 | Vitvi07g04089\_t001 |  | Vitvi14g00126\_t001 |  | Vitvi05g01762\_t001 |  |  |  |  |  |  |  |  |
| 2 | Vitvi07g00373\_t001 |  | Vitvi14g00125\_t001 |  | \| \| |  |  |  |  |  |  |  |  |
| 2 | Vitvi07g00374\_t001 |  | \| \| |  | Vitvi05g00075\_t001 |  |  |  |  |  |  |  |  |
| 2 | Vitvi07g00375\_t001 |  | \| \| |  | Vitvi05g00076\_t001 |  |  |  |  |  |  |  |  |
| 2 | Vitvi07g00376\_t001 |  | \| \| |  | \| \| |  |  |  |  |  |  |  |  |
| 2 | Vitvi07g00378\_t001 |  | \| \| |  | \| \| |  |  |  |  |  |  |  |  |
| 2 | Vitvi07g04090\_t001 |  | \| \| |  | \| \| |  |  |  |  |  |  |  |  |
| 2 | Vitvi07g02211\_t001 |  | \| \| |  | \| \| |  |  |  |  |  |  |  |  |
| 2 | Vitvi07g02212\_t001 |  | \| \| |  | \| \| |  |  |  |  |  |  |  |  |
| 2 | Vitvi07g02213\_t001 |  | \| \| |  | \| \| |  |  |  |  |  |  |  |  |
| 2 | Vitvi07g00380\_t001 |  | \| \| |  | \| \| |  |  |  |  |  |  |  |  |
| 2 | Vitvi07g02214\_t001 |  | Vitvi14g00123\_t001 |  | Vitvi05g00077\_t001 |  |  |  |  |  |  |  |  |
| 2 | Vitvi07g00381\_t001 |  | \| \| |  | Vitvi05g04017\_t001 |  |  |  |  |  |  |  |  |
| 2 | Vitvi07g00382\_t001 |  | Vitvi14g00122\_t001 |  | Vitvi05g01765\_t001 |  |  |  |  |  |  |  |  |
| 2 | Vitvi07g04091\_t001 |  | \| \| |  | \| \| |  |  |  |  |  |  |  |  |
| 2 | Vitvi07g00383\_t001 |  | \| \| |  | \| \| |  |  |  |  |  |  |  |  |
| 2 | Vitvi07g00384\_t001 |  | \| \| |  | Vitvi05g00081\_t002 |  |  |  |  |  |  |  |  |
| 2 | Vitvi07g02216\_t001 |  | Vitvi14g02479\_t001 |  | Vitvi05g01766\_t001 |  |  |  |  |  |  |  |  |
| 2 | Vitvi07g02217\_t001 |  | \| \| |  | \| \| |  |  |  |  |  |  |  |  |
| 2 | Vitvi07g00386\_t001 |  | \| \| |  | \| \| |  |  |  |  |  |  |  |  |
| 2 | Vitvi07g00387\_t001 |  | \| \| |  | \| \| |  |  |  |  |  |  |  |  |
| 2 | Vitvi07g00388\_t001 |  | \| \| |  | \| \| |  |  |  |  |  |  |  |  |
| 2 | Vitvi07g00389\_t001 |  | \| \| |  | \| \| |  |  |  |  |  |  |  |  |
| 2 | Vitvi07g00390\_t001 |  | Vitvi14g00113\_t001 |  | \| \| |  |  |  |  |  |  |  |  |
| 2 | Vitvi07g00391\_t001 |  | \| \| |  | \| \| |  |  |  |  |  |  |  |  |
| 2 | Vitvi07g00392\_t001 |  | \| \| |  | \| \| |  |  |  |  |  |  |  |  |
| 2 | Vitvi07g00393\_t001 |  | \| \| |  | Vitvi05g00084\_t001 |  |  |  |  |  |  |  |  |
| 2 | Vitvi07g04092\_t001 |  | \| \| |  | \| \| |  |  |  |  |  |  |  |  |
| 2 | Vitvi07g00394\_t001 |  | \| \| |  | \| \| |  |  |  |  |  |  |  |  |
| 2 | Vitvi07g02218\_t003 |  | \| \| |  | \| \| |  |  |  |  |  |  |  |  |
| 2 | Vitvi07g04093\_t001 |  | \| \| |  | \| \| |  |  |  |  |  |  |  |  |
| 2 | Vitvi07g00395\_t001 |  | \| \| |  | \| \| |  |  |  |  |  |  |  |  |
| 2 | Vitvi07g04094\_t001 |  | \| \| |  | \| \| |  |  |  |  |  |  |  |  |
| 2 | Vitvi07g00396\_t001 |  | \| \| |  | \| \| |  |  |  |  |  |  |  |  |
| 2 | Vitvi07g00399\_t001 |  | \| \| |  | \| \| |  |  |  |  |  |  |  |  |
| 2 | Vitvi07g04095\_t001 |  | \| \| |  | \| \| |  |  |  |  |  |  |  |  |
| 2 | Vitvi07g04096\_t001 |  | \| \| |  | \| \| |  |  |  |  |  |  |  |  |
| 2 | Vitvi07g00400\_t001 |  | \| \| |  | \| \| |  |  |  |  |  |  |  |  |
| 2 | Vitvi07g00401\_t001 |  | \| \| |  | Vitvi05g00089\_t001 |  |  |  |  |  |  |  |  |
| 2 | Vitvi07g02220\_t001 |  | \| \| |  | \| \| |  |  |  |  |  |  |  |  |
| 2 | Vitvi07g02221\_t001 |  | \| \| |  | \| \| |  |  |  |  |  |  |  |  |
| 2 | Vitvi07g00402\_t001 |  | Vitvi14g04028\_t002 |  | Vitvi05g00097\_t001 |  |  |  |  |  |  |  |  |
| 2 | Vitvi07g00403\_t001 |  | \| \| |  | Vitvi05g00098\_t001 |  |  |  |  |  |  |  |  |
| 2 | Vitvi07g00404\_t001 |  | Vitvi14g02476\_t001 |  | \| \| |  |  |  |  |  |  |  |  |
| 2 | Vitvi07g00405\_t001 |  | \| \| |  | \| \| |  |  |  |  |  |  |  |  |
| 2 | Vitvi07g00406\_t001 |  | \| \| |  | \| \| |  |  |  |  |  |  |  |  |
| 2 | Vitvi07g04097\_t001 |  | \| \| |  | \| \| |  |  |  |  |  |  |  |  |
| 2 | Vitvi07g00407\_t001 |  | \| \| |  | \| \| |  |  |  |  |  |  |  |  |
| 2 | Vitvi07g00408\_t001 |  | Vitvi14g00102\_t001 |  | \| \| |  |  |  |  |  |  |  |  |
| 2 | Vitvi07g02222\_t001 |  | \| \| |  | \| \| |  |  |  |  |  |  |  |  |
| 2 | Vitvi07g00409\_t001 |  | \| \| |  | \| \| |  |  |  |  |  |  |  |  |
| 2 | Vitvi07g00410\_t002 |  | \| \| |  | Vitvi05g00106\_t001 |  |  |  |  |  |  |  |  |
| 2 | Vitvi07g00411\_t001 |  | \| \| |  | \| \| |  |  |  |  |  |  |  |  |
| 2 | Vitvi07g00412\_t001 |  | \| \| |  | \| \| |  |  |  |  |  |  |  |  |
| 2 | Vitvi07g00413\_t001 |  | Vitvi14g00094\_t001 |  | Vitvi05g00108\_t001 |  |  |  |  |  |  |  |  |
| 2 | Vitvi07g04098\_t001 |  | \| \| |  | \| \| |  |  |  |  |  |  |  |  |
| 2 | Vitvi07g00414\_t003 |  | \| \| |  | \| \| |  |  |  |  |  |  |  |  |
| 2 | Vitvi07g02224\_t001 |  | Vitvi14g02473\_t001 |  | \| \| |  |  |  |  |  |  |  |  |
| 2 | Vitvi07g00415\_t001 |  | \| \| |  | \| \| |  |  |  |  |  |  |  |  |
| 2 | Vitvi07g00416\_t001 |  | \| \| |  | \| \| |  |  |  |  |  |  |  |  |
| 2 | Vitvi07g00417\_t001 |  | \| \| |  | \| \| |  |  |  |  |  |  |  |  |
| 2 | Vitvi07g00418\_t001 |  | \| \| |  | Vitvi05g00110\_t001 |  |  |  |  |  |  |  |  |
| 2 | Vitvi07g04099\_t001 |  | \| \| |  | Vitvi05g00112\_t001 |  |  |  |  |  |  |  |  |
| 2 | Vitvi07g04100\_t001 |  | \| \| |  | \| \| |  |  |  |  |  |  |  |  |
| 2 | Vitvi07g00420\_t001 |  | \| \| |  | \| \| |  |  |  |  |  |  |  |  |
| 2 | Vitvi07g00421\_t003 |  | \| \| |  | \| \| |  |  |  |  |  |  |  |  |
| 2 | Vitvi07g00422\_t001 |  | \| \| |  | \| \| |  |  |  |  |  |  |  |  |
| 2 | Vitvi07g02226\_t001 |  | \| \| |  | \| \| |  |  |  |  |  |  |  |  |
| 2 | Vitvi07g04101\_t001 |  | \| \| |  | \| \| |  |  |  |  |  |  |  |  |
| 2 | Vitvi07g00423\_t001.3.6037826e |  | Vitvi14g00090\_t001 |  | Vitvi05g00114\_t001 |  |  |  |  |  |  |  |  |
| 2 | Vitvi07g02228\_t001 |  | \| \| |  | Vitvi05g01772\_t001 |  |  |  |  |  |  |  |  |
| 2 | Vitvi07g00424\_t001 |  | \| \| |  | \| \| |  |  |  |  |  |  |  |  |
| 2 | Vitvi07g00425\_t001 |  | \| \| |  | \| \| |  |  |  |  |  |  |  |  |
| 2 | Vitvi07g00426\_t001 |  | \| \| |  | \| \| |  |  |  |  |  |  |  |  |
| 2 | Vitvi07g00427\_t001 |  | Vitvi14g00089\_t001 |  | \| \| |  |  |  |  |  |  |  |  |
| 2 | Vitvi07g00428\_t001 |  | \| \| |  | Vitvi05g00127\_t001 |  |  |  |  |  |  |  |  |
| 2 | Vitvi07g00429\_t001 |  | \| \| |  | \| \| |  |  |  |  |  |  |  |  |
| 2 | Vitvi07g00430\_t001 |  | \| \| |  | \| \| |  |  |  |  |  |  |  |  |
| 2 | Vitvi07g02229\_t001 |  | \| \| |  | \| \| |  |  |  |  |  |  |  |  |
| 2 | Vitvi07g00431\_t001 |  | \| \| |  | Vitvi05g00139\_t001 |  |  |  |  |  |  |  |  |
| 2 | Vitvi07g04102\_t001 |  | \| \| |  | \| \| |  |  |  |  |  |  |  |  |
| 2 | Vitvi07g04103\_t001 |  | \| \| |  | \| \| |  |  |  |  |  |  |  |  |
| 2 | Vitvi07g02230\_t001 |  | \| \| |  | Vitvi05g01783\_t001 |  |  |  |  |  |  |  |  |
| 2 | Vitvi07g00434\_t001 |  | \| \| |  | Vitvi05g00145\_t001 |  |  |  |  |  |  |  |  |
| 2 | Vitvi07g00435\_t001 |  | \| \| |  | Vitvi05g00147\_t001 |  |  |  |  |  |  |  |  |
| 2 | Vitvi07g04104\_t001 |  | \| \| |  | \| \| |  |  |  |  |  |  |  |  |
| 2 | Vitvi07g00436\_t001 |  | \| \| |  | \| \| |  |  |  |  |  |  |  |  |
| 2 | Vitvi07g04105\_t001 |  | \| \| |  | \| \| |  |  |  |  |  |  |  |  |
| 2 | Vitvi07g00437\_t001 |  | Vitvi14g00080\_t001 |  | \| \| |  |  |  |  |  |  |  |  |
| 2 | Vitvi07g00438\_t001 |  | \| \| |  | \| \| |  |  |  |  |  |  |  |  |
| 2 | Vitvi07g00439\_t001 |  | Vitvi14g02465\_t001 |  | Vitvi05g00150\_t001 |  |  |  |  |  |  |  |  |
| 2 | Vitvi07g00441\_t001 |  | \| \| |  | \| \| |  |  |  |  |  |  |  |  |
| 2 | Vitvi07g04106\_t001 |  | \| \| |  | \| \| |  |  |  |  |  |  |  |  |
| 2 | Vitvi07g00442\_t001 |  | \| \| |  | \| \| |  |  |  |  |  |  |  |  |
| 2 | Vitvi07g00444\_t001 |  | \| \| |  | \| \| |  |  |  |  |  |  |  |  |
| 2 | Vitvi07g04107\_t001 |  | \| \| |  | \| \| |  |  |  |  |  |  |  |  |
| 2 | Vitvi07g00445\_t001 |  | \| \| |  | \| \| |  |  |  |  |  |  |  |  |
| 2 | Vitvi07g00446\_t001 |  | Vitvi14g00072\_t001 |  | \| \| |  |  |  |  |  |  |  |  |
| 2 | Vitvi07g00448\_t001 |  | \| \| |  | \| \| |  |  |  |  |  |  |  |  |
| 2 | Vitvi07g00449\_t001 |  | \| \| |  | \| \| |  |  |  |  |  |  |  |  |
| 2 | Vitvi07g00450\_t001 |  | \| \| |  | \| \| |  |  |  |  |  |  |  |  |
| 2 | Vitvi07g02231\_t001 |  | \| \| |  | \| \| |  |  |  |  |  |  |  |  |
| 2 | Vitvi07g04108\_t001 |  | \| \| |  | \| \| |  |  |  |  |  |  |  |  |
| 2 | Vitvi07g04109\_t001 |  | \| \| |  | \| \| |  |  |  |  |  |  |  |  |
| 2 | Vitvi07g04110\_t001 |  | \| \| |  | \| \| |  |  |  |  |  |  |  |  |
| 2 | Vitvi07g02233\_t001 |  | \| \| |  | \| \| |  |  |  |  |  |  |  |  |
| 2 | Vitvi07g02234\_t001 |  | Vitvi14g02462\_t001 |  | \| \| |  |  |  |  |  |  |  |  |
| 2 | Vitvi07g00452\_t003 |  | \| \| |  | \| \| |  |  |  |  |  |  |  |  |
| 2 | Vitvi07g00453\_t001 |  | \| \| |  | Vitvi05g00163\_t001 |  |  |  |  |  |  |  |  |
| 2 | Vitvi07g00454\_t001 |  | \| \| |  | \| \| |  |  |  |  |  |  |  |  |
| 2 | Vitvi07g00455\_t001 |  | \| \| |  | Vitvi05g00166\_t001 |  |  |  |  |  |  |  |  |
| 2 | Vitvi07g00456\_t001 |  | Vitvi14g00067\_t001 |  | \| \| |  |  |  |  |  |  |  |  |
| 2 | Vitvi07g00457\_t001 |  | Vitvi14g02458\_t001 |  | Vitvi05g00170\_t001 |  |  |  |  |  |  |  |  |
| 1 | Vitvi07g00458\_t001 |  | Vitvi14g02456\_t001 |  |  |  |  |  |  |  |  |  |  |
| 1 | Vitvi07g00459\_t001 |  | \| \| |  |  |  |  |  |  |  |  |  |  |
| 1 | Vitvi07g04111\_t001 |  | \| \| |  |  |  |  |  |  |  |  |  |  |
| 1 | Vitvi07g00460\_t001 |  | \| \| |  |  |  |  |  |  |  |  |  |  |
| 1 | Vitvi07g00463\_t001 |  | \| \| |  |  |  |  |  |  |  |  |  |  |
| 1 | Vitvi07g00464\_t001 |  | Vitvi14g04009\_t001 |  |  |  |  |  |  |  |  |  |  |
| 1 | Vitvi07g00465\_t001 |  | \| \| |  |  |  |  |  |  |  |  |  |  |
| 1 | Vitvi07g04112\_t001 |  | \| \| |  |  |  |  |  |  |  |  |  |  |
| 1 | Vitvi07g04113\_t001 |  | Vitvi14g00059\_t001 |  |  |  |  |  |  |  |  |  |  |
| 1 | Vitvi07g00467\_t001 |  | \| \| |  |  |  |  |  |  |  |  |  |  |
| 1 | Vitvi07g00468\_t001 |  | \| \| |  |  |  |  |  |  |  |  |  |  |
| 1 | Vitvi07g00469\_t001 |  | \| \| |  |  |  |  |  |  |  |  |  |  |
| 1 | Vitvi07g00470\_t001 |  | \| \| |  |  |  |  |  |  |  |  |  |  |
| 1 | Vitvi07g02243\_t001 |  | \| \| |  |  |  |  |  |  |  |  |  |  |
| 1 | Vitvi07g02244\_t001 |  | \| \| |  |  |  |  |  |  |  |  |  |  |
| 1 | Vitvi07g00471\_t001 |  | Vitvi14g00055\_t001 |  |  |  |  |  |  |  |  |  |  |
| 1 | Vitvi07g00472\_t002 |  | Vitvi14g00053\_t003 |  |  |  |  |  |  |  |  |  |  |
| 1 | Vitvi07g04114\_t001 |  | \| \| |  |  |  |  |  |  |  |  |  |  |
| 1 | Vitvi07g00474\_t001 |  | \| \| |  |  |  |  |  |  |  |  |  |  |
| 1 | Vitvi07g00475\_t001 |  | \| \| |  |  |  |  |  |  |  |  |  |  |
| 1 | Vitvi07g00476\_t001 |  | \| \| |  |  |  |  |  |  |  |  |  |  |
| 1 | Vitvi07g00477\_t001 |  | \| \| |  |  |  |  |  |  |  |  |  |  |
| 1 | Vitvi07g00478\_t001 |  | Vitvi14g02447\_t001 |  |  |  |  |  |  |  |  |  |  |
| 1 | Vitvi07g00479\_t001 |  | \| \| |  |  |  |  |  |  |  |  |  |  |
| 1 | Vitvi07g04115\_t001 |  | \| \| |  |  |  |  |  |  |  |  |  |  |
| 1 | Vitvi07g00481\_t001 |  | \| \| |  |  |  |  |  |  |  |  |  |  |
| 1 | Vitvi07g02245\_t001 |  | \| \| |  |  |  |  |  |  |  |  |  |  |
| 1 | Vitvi07g00482\_t001 |  | \| \| |  |  |  |  |  |  |  |  |  |  |
| 1 | Vitvi07g00483\_t001 |  | \| \| |  |  |  |  |  |  |  |  |  |  |
| 1 | Vitvi07g00484\_t002 |  | \| \| |  |  |  |  |  |  |  |  |  |  |
| 1 | Vitvi07g00485\_t001 |  | \| \| |  |  |  |  |  |  |  |  |  |  |
| 1 | Vitvi07g00486\_t001 |  | \| \| |  |  |  |  |  |  |  |  |  |  |
| 1 | Vitvi07g02246\_t001 |  | \| \| |  |  |  |  |  |  |  |  |  |  |
| 1 | Vitvi07g02247\_t001 |  | Vitvi14g02444\_t001 |  |  |  |  |  |  |  |  |  |  |
| 1 | Vitvi07g00487\_t001 |  | \| \| |  |  |  |  |  |  |  |  |  |  |
| 1 | Vitvi07g00488\_t001 |  | Vitvi14g00043\_t001 |  |  |  |  |  |  |  |  |  |  |
| 1 | Vitvi07g00490\_t001 |  | \| \| |  |  |  |  |  |  |  |  |  |  |
| 1 | Vitvi07g00491\_t001 |  | \| \| |  |  |  |  |  |  |  |  |  |  |
| 1 | Vitvi07g00492\_t001 |  | \| \| |  |  |  |  |  |  |  |  |  |  |
| 1 | Vitvi07g04116\_t001 |  | \| \| |  |  |  |  |  |  |  |  |  |  |
| 1 | Vitvi07g00493\_t001 |  | \| \| |  |  |  |  |  |  |  |  |  |  |
| 1 | Vitvi07g00494\_t001 |  | Vitvi14g00039\_t001 |  |  |  |  |  |  |  |  |  |  |
| 1 | Vitvi07g00495\_t001 |  | Vitvi14g00037\_t001 |  |  |  |  |  |  |  |  |  |  |
| 0 | Vitvi07g04117\_t001 |  |  |  |  |  |  |  |  |  |  |  |  |
| 0 | Vitvi07g00496\_t001 |  |  |  |  |  |  |  |  |  |  |  |  |
| 0 | Vitvi07g00497\_t001 |  |  |  |  |  |  |  |  |  |  |  |  |
| 0 | Vitvi07g04118\_t001 |  |  |  |  |  |  |  |  |  |  |  |  |
| 0 | Vitvi07g02248\_t001 |  |  |  |  |  |  |  |  |  |  |  |  |
| 0 | Vitvi07g00502\_t001 |  |  |  |  |  |  |  |  |  |  |  |  |
| 0 | Vitvi07g04119\_t001 |  |  |  |  |  |  |  |  |  |  |  |  |
| 0 | Vitvi07g04120\_t001 |  |  |  |  |  |  |  |  |  |  |  |  |
| 0 | Vitvi07g04121\_t001 |  |  |  |  |  |  |  |  |  |  |  |  |
| 0 | Vitvi07g00505\_t001 |  |  |  |  |  |  |  |  |  |  |  |  |
| 0 | Vitvi07g00506\_t002 |  |  |  |  |  |  |  |  |  |  |  |  |
| 0 | Vitvi07g00507\_t001 |  |  |  |  |  |  |  |  |  |  |  |  |
| 0 | Vitvi07g00508\_t001 |  |  |  |  |  |  |  |  |  |  |  |  |
| 0 | Vitvi07g00509\_t001 |  |  |  |  |  |  |  |  |  |  |  |  |
| 0 | Vitvi07g04122\_t001 |  |  |  |  |  |  |  |  |  |  |  |  |
| 0 | Vitvi07g00510\_t001 |  |  |  |  |  |  |  |  |  |  |  |  |
| 1 | Vitvi07g02249\_t003 |  | Vitvi05g00276\_t001 |  |  |  |  |  |  |  |  |  |  |
| 1 | Vitvi07g02074\_t001 |  | \| \| |  |  |  |  |  |  |  |  |  |  |
| 1 | Vitvi07g04123\_t001 |  | \| \| |  |  |  |  |  |  |  |  |  |  |
| 1 | Vitvi07g00513\_t001 |  | \| \| |  |  |  |  |  |  |  |  |  |  |
| 1 | Vitvi07g00515\_t001 |  | Vitvi05g00275\_t001 |  |  |  |  |  |  |  |  |  |  |
| 1 | Vitvi07g00516\_t001 |  | \| \| |  |  |  |  |  |  |  |  |  |  |
| 1 | Vitvi07g00517\_t001 |  | \| \| |  |  |  |  |  |  |  |  |  |  |
| 1 | Vitvi07g04124\_t001 |  | \| \| |  |  |  |  |  |  |  |  |  |  |
| 1 | Vitvi07g02251\_t002 |  | \| \| |  |  |  |  |  |  |  |  |  |  |
| 1 | Vitvi07g00518\_t001 |  | \| \| |  |  |  |  |  |  |  |  |  |  |
| 1 | Vitvi07g00520\_t001 |  | \| \| |  |  |  |  |  |  |  |  |  |  |
| 1 | Vitvi07g00521\_t001 |  | Vitvi05g00271\_t001 |  |  |  |  |  |  |  |  |  |  |
| 1 | Vitvi07g00522\_t001 |  | \| \| |  |  |  |  |  |  |  |  |  |  |
| 1 | Vitvi07g00523\_t001 |  | \| \| |  |  |  |  |  |  |  |  |  |  |
| 1 | Vitvi07g00524\_t001 |  | \| \| |  |  |  |  |  |  |  |  |  |  |
| 1 | Vitvi07g00525\_t001 |  | \| \| |  |  |  |  |  |  |  |  |  |  |
| 1 | Vitvi07g00526\_t001 |  | \| \| |  |  |  |  |  |  |  |  |  |  |
| 1 | Vitvi07g00527\_t001 |  | \| \| |  |  |  |  |  |  |  |  |  |  |
| 1 | Vitvi07g00529\_t002 |  | \| \| |  |  |  |  |  |  |  |  |  |  |
| 1 | Vitvi07g00530\_t001 |  | \| \| |  |  |  |  |  |  |  |  |  |  |
| 1 | Vitvi07g04125\_t001 |  | \| \| |  |  |  |  |  |  |  |  |  |  |
| 1 | Vitvi07g00531\_t001 |  | \| \| |  |  |  |  |  |  |  |  |  |  |
| 1 | Vitvi07g04126\_t001 |  | \| \| |  |  |  |  |  |  |  |  |  |  |
| 1 | Vitvi07g00532\_t001 |  | Vitvi05g01835\_t001 |  |  |  |  |  |  |  |  |  |  |
| 1 | Vitvi07g00533\_t001 |  | \| \| |  |  |  |  |  |  |  |  |  |  |
| 1 | Vitvi07g04127\_t001 |  | \| \| |  |  |  |  |  |  |  |  |  |  |
| 1 | Vitvi07g04128\_t001 |  | \| \| |  |  |  |  |  |  |  |  |  |  |
| 1 | Vitvi07g00534\_t001 |  | Vitvi05g00261\_t001 |  |  |  |  |  |  |  |  |  |  |
| 1 | Vitvi07g04129\_t001 |  | \| \| |  |  |  |  |  |  |  |  |  |  |
| 1 | Vitvi07g00535\_t001 |  | \| \| |  |  |  |  |  |  |  |  |  |  |
| 1 | Vitvi07g00537\_t001 |  | \| \| |  |  |  |  |  |  |  |  |  |  |
| 1 | Vitvi07g00538\_t001 |  | \| \| |  |  |  |  |  |  |  |  |  |  |
| 1 | Vitvi07g02253\_t002 |  | \| \| |  |  |  |  |  |  |  |  |  |  |
| 1 | Vitvi07g00539\_t001 |  | \| \| |  |  |  |  |  |  |  |  |  |  |
| 1 | Vitvi07g04130\_t001 |  | \| \| |  |  |  |  |  |  |  |  |  |  |
| 1 | Vitvi07g04131\_t001 |  | Vitvi05g00253\_t001 |  |  |  |  |  |  |  |  |  |  |
| 0 | Vitvi07g02254\_t001 |  |  |  |  |  |  |  |  |  |  |  |  |
| 0 | Vitvi07g00540\_t001 |  |  |  |  |  |  |  |  |  |  |  |  |
| 0 | Vitvi07g00542\_t001 |  |  |  |  |  |  |  |  |  |  |  |  |
| 0 | Vitvi07g04132\_t001 |  |  |  |  |  |  |  |  |  |  |  |  |
| 0 | Vitvi07g00543\_t001 |  |  |  |  |  |  |  |  |  |  |  |  |
| 0 | Vitvi07g00544\_t001 |  |  |  |  |  |  |  |  |  |  |  |  |
| 0 | Vitvi07g00545\_t001 |  |  |  |  |  |  |  |  |  |  |  |  |
| 0 | Vitvi07g00549\_t001 |  |  |  |  |  |  |  |  |  |  |  |  |
| 0 | Vitvi07g04133\_t001 |  |  |  |  |  |  |  |  |  |  |  |  |
| 0 | Vitvi07g00550\_t001 |  |  |  |  |  |  |  |  |  |  |  |  |
| 0 | Vitvi07g04134\_t001 |  |  |  |  |  |  |  |  |  |  |  |  |
| 0 | Vitvi07g04135\_t001 |  |  |  |  |  |  |  |  |  |  |  |  |
| 0 | Vitvi07g00551\_t001 |  |  |  |  |  |  |  |  |  |  |  |  |
| 0 | Vitvi07g04136\_t001 |  |  |  |  |  |  |  |  |  |  |  |  |
| 0 | Vitvi07g04137\_t001 |  |  |  |  |  |  |  |  |  |  |  |  |
| 0 | Vitvi07g00554\_t001 |  |  |  |  |  |  |  |  |  |  |  |  |
| 0 | Vitvi07g04138\_t001 |  |  |  |  |  |  |  |  |  |  |  |  |
| 1 | Vitvi07g04139\_t001 |  | Vitvi05g01909\_t001 |  |  |  |  |  |  |  |  |  |  |
| 1 | Vitvi07g00559\_t001 |  | \| \| |  |  |  |  |  |  |  |  |  |  |
| 1 | Vitvi07g04140\_t001 |  | \| \| |  |  |  |  |  |  |  |  |  |  |
| 1 | Vitvi07g04141\_t001 |  | \| \| |  |  |  |  |  |  |  |  |  |  |
| 1 | Vitvi07g04142\_t001 |  | \| \| |  |  |  |  |  |  |  |  |  |  |
| 1 | Vitvi07g02259\_t001 |  | \| \| |  |  |  |  |  |  |  |  |  |  |
| 1 | Vitvi07g04143\_t001 |  | \| \| |  |  |  |  |  |  |  |  |  |  |
| 1 | Vitvi07g04144\_t001 |  | \| \| |  |  |  |  |  |  |  |  |  |  |
| 1 | Vitvi07g04145\_t001 |  | \| \| |  |  |  |  |  |  |  |  |  |  |
| 1 | Vitvi07g02260\_t001 |  | \| \| |  |  |  |  |  |  |  |  |  |  |
| 1 | Vitvi07g00561\_t001 |  | \| \| |  |  |  |  |  |  |  |  |  |  |
| 1 | Vitvi07g00563\_t001 |  | \| \| |  |  |  |  |  |  |  |  |  |  |
| 1 | Vitvi07g00564\_t001 |  | \| \| |  |  |  |  |  |  |  |  |  |  |
| 1 | Vitvi07g04146\_t001 |  | \| \| |  |  |  |  |  |  |  |  |  |  |
| 1 | Vitvi07g00565\_t001 |  | \| \| |  |  |  |  |  |  |  |  |  |  |
| 1 | Vitvi07g04147\_t001 |  | \| \| |  |  |  |  |  |  |  |  |  |  |
| 1 | Vitvi07g02261\_t001 |  | Vitvi05g01913\_t001 |  |  |  |  |  |  |  |  |  |  |
| 1 | Vitvi07g02262\_t001 |  | Vitvi05g01915\_t001 |  |  |  |  |  |  |  |  |  |  |
| 1 | Vitvi07g04148\_t001 |  | \| \| |  |  |  |  |  |  |  |  |  |  |
| 1 | Vitvi07g00567\_t001 |  | \| \| |  |  |  |  |  |  |  |  |  |  |
| 1 | Vitvi07g00569\_t001 |  | \| \| |  |  |  |  |  |  |  |  |  |  |
| 1 | Vitvi07g00571\_t001 |  | \| \| |  |  |  |  |  |  |  |  |  |  |
| 1 | Vitvi07g00572\_t001 |  | \| \| |  |  |  |  |  |  |  |  |  |  |
| 1 | Vitvi07g00573\_t001 |  | \| \| |  |  |  |  |  |  |  |  |  |  |
| 1 | Vitvi07g00574\_t001 |  | \| \| |  |  |  |  |  |  |  |  |  |  |
| 1 | Vitvi07g00575\_t001 |  | \| \| |  |  |  |  |  |  |  |  |  |  |
| 1 | Vitvi07g00576\_t001 |  | Vitvi05g01922\_t001 |  |  |  |  |  |  |  |  |  |  |
| 1 | Vitvi07g00577\_t001 |  | \| \| |  |  |  |  |  |  |  |  |  |  |
| 1 | Vitvi07g04149\_t001 |  | \| \| |  |  |  |  |  |  |  |  |  |  |
| 1 | Vitvi07g00578\_t001 |  | Vitvi05g00710\_t001 |  |  |  |  |  |  |  |  |  |  |
| 1 | Vitvi07g00579\_t001 |  | \| \| |  |  |  |  |  |  |  |  |  |  |
| 1 | Vitvi07g00580\_t002 |  | \| \| |  |  |  |  |  |  |  |  |  |  |
| 1 | Vitvi07g00581\_t001 |  | \| \| |  |  |  |  |  |  |  |  |  |  |
| 1 | Vitvi07g00582\_t001 |  | Vitvi05g00713\_t001 |  |  |  |  |  |  |  |  |  |  |
| 1 | Vitvi07g04150\_t001 |  | \| \| |  |  |  |  |  |  |  |  |  |  |
| 1 | Vitvi07g00583\_t001 |  | \| \| |  |  |  |  |  |  |  |  |  |  |
| 1 | Vitvi07g00586\_t001 |  | \| \| |  |  |  |  |  |  |  |  |  |  |
| 1 | Vitvi07g00587\_t001 |  | \| \| |  |  |  |  |  |  |  |  |  |  |
| 1 | Vitvi07g00588\_t001 |  | \| \| |  |  |  |  |  |  |  |  |  |  |
| 1 | Vitvi07g04151\_t001 |  | \| \| |  |  |  |  |  |  |  |  |  |  |
| 1 | Vitvi07g02062\_t001 |  | Vitvi05g01722\_t001 |  |  |  |  |  |  |  |  |  |  |
| 1 | Vitvi07g02063\_t001 |  | \| \| |  |  |  |  |  |  |  |  |  |  |
| 1 | Vitvi07g04152\_t001 |  | \| \| |  |  |  |  |  |  |  |  |  |  |
| 1 | Vitvi07g02064\_t001 |  | \| \| |  |  |  |  |  |  |  |  |  |  |
| 1 | Vitvi07g00590\_t001 |  | \| \| |  |  |  |  |  |  |  |  |  |  |
| 1 | Vitvi07g02070\_t001 |  | \| \| |  |  |  |  |  |  |  |  |  |  |
| 1 | Vitvi07g04153\_t001 |  | \| \| |  |  |  |  |  |  |  |  |  |  |
| 1 | Vitvi07g02067\_t001 |  | Vitvi05g00715\_t001 |  |  |  |  |  |  |  |  |  |  |
| 0 | Vitvi07g02065\_t001 |  |  |  |  |  |  |  |  |  |  |  |  |
| 0 | Vitvi07g00591\_t001 |  |  |  |  |  |  |  |  |  |  |  |  |
| 0 | Vitvi07g00592\_t001 |  |  |  |  |  |  |  |  |  |  |  |  |
| 0 | Vitvi07g02068\_t001 |  |  |  |  |  |  |  |  |  |  |  |  |
| 1 | Vitvi07g00594\_t001 |  | Vitvi05g00733\_t001 |  |  |  |  |  |  |  |  |  |  |
| 1 | Vitvi07g04154\_t001 |  | \| \| |  |  |  |  |  |  |  |  |  |  |
| 1 | Vitvi07g02266\_t001 |  | \| \| |  |  |  |  |  |  |  |  |  |  |
| 1 | Vitvi07g04155\_t001 |  | \| \| |  |  |  |  |  |  |  |  |  |  |
| 1 | Vitvi07g00595\_t002 |  | Vitvi05g00739\_t001 |  |  |  |  |  |  |  |  |  |  |
| 1 | Vitvi07g04156\_t001 |  | Vitvi05g00742\_t001 |  |  |  |  |  |  |  |  |  |  |
| 1 | Vitvi07g00597\_t001 |  | \| \| |  |  |  |  |  |  |  |  |  |  |
| 1 | Vitvi07g00598\_t001 |  | Vitvi05g01732\_t001 |  |  |  |  |  |  |  |  |  |  |
| 1 | Vitvi07g04157\_t005 |  | \| \| |  |  |  |  |  |  |  |  |  |  |
| 1 | Vitvi07g00601\_t001 |  | \| \| |  |  |  |  |  |  |  |  |  |  |
| 1 | Vitvi07g02267\_t001 |  | Vitvi05g01958\_t001 |  |  |  |  |  |  |  |  |  |  |
| 1 | Vitvi07g00603\_t001 |  | \| \| |  |  |  |  |  |  |  |  |  |  |
| 1 | Vitvi07g00604\_t001 |  | \| \| |  |  |  |  |  |  |  |  |  |  |
| 1 | Vitvi07g00605\_t001 |  | \| \| |  |  |  |  |  |  |  |  |  |  |
| 1 | Vitvi07g02268\_t001 |  | \| \| |  |  |  |  |  |  |  |  |  |  |
| 1 | Vitvi07g02269\_t001 |  | \| \| |  |  |  |  |  |  |  |  |  |  |
| 1 | Vitvi07g00606\_t001 |  | \| \| |  |  |  |  |  |  |  |  |  |  |
| 1 | Vitvi07g00607\_t001 |  | \| \| |  |  |  |  |  |  |  |  |  |  |
| 1 | Vitvi07g02270\_t001 |  | \| \| |  |  |  |  |  |  |  |  |  |  |
| 1 | Vitvi07g00610\_t001 |  | \| \| |  |  |  |  |  |  |  |  |  |  |
| 1 | Vitvi07g00612\_t001 |  | \| \| |  |  |  |  |  |  |  |  |  |  |
| 1 | Vitvi07g00613\_t001 |  | \| \| |  |  |  |  |  |  |  |  |  |  |
| 1 | Vitvi07g00614\_t001 |  | \| \| |  |  |  |  |  |  |  |  |  |  |
| 1 | Vitvi07g00615\_t001 |  | Vitvi05g00757\_t001 |  |  |  |  |  |  |  |  |  |  |
| 1 | Vitvi07g04158\_t001 |  | \| \| |  |  |  |  |  |  |  |  |  |  |
| 1 | Vitvi07g02271\_t001 |  | \| \| |  |  |  |  |  |  |  |  |  |  |
| 1 | Vitvi07g04159\_t001 |  | \| \| |  |  |  |  |  |  |  |  |  |  |
| 1 | Vitvi07g00618\_t002 |  | \| \| |  |  |  |  |  |  |  |  |  |  |
| 1 | Vitvi07g00619\_t001 |  | \| \| |  |  |  |  |  |  |  |  |  |  |
| 1 | Vitvi07g00620\_t001 |  | \| \| |  |  |  |  |  |  |  |  |  |  |
| 1 | Vitvi07g00621\_t002 |  | Vitvi05g00759\_t001 |  |  |  |  |  |  |  |  |  |  |
| 1 | Vitvi07g00622\_t002 |  | \| \| |  |  |  |  |  |  |  |  |  |  |
| 1 | Vitvi07g00623\_t001 |  | \| \| |  |  |  |  |  |  |  |  |  |  |
| 1 | Vitvi07g00624\_t001 |  | \| \| |  |  |  |  |  |  |  |  |  |  |
| 1 | Vitvi07g00625\_t001 |  | \| \| |  |  |  |  |  |  |  |  |  |  |
| 1 | Vitvi07g04160\_t001 |  | \| \| |  |  |  |  |  |  |  |  |  |  |
| 1 | Vitvi07g00626\_t001 |  | Vitvi05g00764\_t001 |  |  |  |  |  |  |  |  |  |  |
| 1 | Vitvi07g00627\_t001 |  | \| \| |  |  |  |  |  |  |  |  |  |  |
| 1 | Vitvi07g00628\_t001 |  | Vitvi05g00767\_t001 |  |  |  |  |  |  |  |  |  |  |
| 1 | Vitvi07g00629\_t001 |  | \| \| |  |  |  |  |  |  |  |  |  |  |
| 1 | Vitvi07g00630\_t002 |  | \| \| |  |  |  |  |  |  |  |  |  |  |
| 1 | Vitvi07g00633\_t002 |  | \| \| |  |  |  |  |  |  |  |  |  |  |
| 1 | Vitvi07g02274\_t001 |  | Vitvi05g01965\_t001 |  |  |  |  |  |  |  |  |  |  |
| 1 | Vitvi07g00634\_t001 |  | \| \| |  |  |  |  |  |  |  |  |  |  |
| 1 | Vitvi07g04161\_t001 |  | \| \| |  |  |  |  |  |  |  |  |  |  |
| 1 | Vitvi07g00635\_t001 |  | \| \| |  |  |  |  |  |  |  |  |  |  |
| 1 | Vitvi07g04162\_t001 |  | \| \| |  |  |  |  |  |  |  |  |  |  |
| 1 | Vitvi07g00636\_t001 |  | Vitvi05g00784\_t001 |  |  |  |  |  |  |  |  |  |  |
| 1 | Vitvi07g00637\_t001 |  | \| \| |  |  |  |  |  |  |  |  |  |  |
| 1 | Vitvi07g02276\_t001 |  | \| \| |  |  |  |  |  |  |  |  |  |  |
| 1 | Vitvi07g00639\_t001 |  | Vitvi05g00788\_t001 |  |  |  |  |  |  |  |  |  |  |
| 1 | Vitvi07g00640\_t001 |  | \| \| |  |  |  |  |  |  |  |  |  |  |
| 1 | Vitvi07g02277\_t001 |  | \| \| |  |  |  |  |  |  |  |  |  |  |
| 1 | Vitvi07g00643\_t001 |  | Vitvi05g00790\_t002 |  |  |  |  |  |  |  |  |  |  |
| 1 | Vitvi07g04163\_t001 |  | \| \| |  |  |  |  |  |  |  |  |  |  |
| 1 | Vitvi07g02278\_t001 |  | \| \| |  |  |  |  |  |  |  |  |  |  |
| 1 | Vitvi07g02279\_t001 |  | \| \| |  |  |  |  |  |  |  |  |  |  |
| 1 | Vitvi07g02280\_t001 |  | \| \| |  |  |  |  |  |  |  |  |  |  |
| 1 | Vitvi07g02281\_t001 |  | \| \| |  |  |  |  |  |  |  |  |  |  |
| 1 | Vitvi07g02282\_t001 |  | \| \| |  |  |  |  |  |  |  |  |  |  |
| 1 | Vitvi07g00644\_t001 |  | \| \| |  |  |  |  |  |  |  |  |  |  |
| 1 | Vitvi07g02283\_t001 |  | \| \| |  |  |  |  |  |  |  |  |  |  |
| 1 | Vitvi07g02284\_t001 |  | \| \| |  |  |  |  |  |  |  |  |  |  |
| 1 | Vitvi07g02285\_t001 |  | \| \| |  |  |  |  |  |  |  |  |  |  |
| 1 | Vitvi07g02286\_t001 |  | \| \| |  |  |  |  |  |  |  |  |  |  |
| 1 | Vitvi07g00647\_t001 |  | \| \| |  |  |  |  |  |  |  |  |  |  |
| 1 | Vitvi07g04164\_t001 |  | \| \| |  |  |  |  |  |  |  |  |  |  |
| 1 | Vitvi07g00649\_t001 |  | \| \| |  |  |  |  |  |  |  |  |  |  |
| 1 | Vitvi07g00650\_t001 |  | \| \| |  |  |  |  |  |  |  |  |  |  |
| 1 | Vitvi07g00652\_t001 |  | Vitvi05g00799\_t001 |  |  |  |  |  |  |  |  |  |  |
| 1 | Vitvi07g00653\_t001 |  | \| \| |  |  |  |  |  |  |  |  |  |  |
| 1 | Vitvi07g00654\_t001 |  | \| \| |  |  |  |  |  |  |  |  |  |  |
| 1 | Vitvi07g00656\_t001 |  | Vitvi05g00802\_t001 |  |  |  |  |  |  |  |  |  |  |
| 1 | Vitvi07g00657\_t001 |  | \| \| |  |  |  |  |  |  |  |  |  |  |
| 1 | Vitvi07g00658\_t002 |  | Vitvi05g00812\_t002 |  |  |  |  |  |  |  |  |  |  |
| 1 | Vitvi07g00659\_t001 |  | Vitvi05g00815\_t001 |  |  |  |  |  |  |  |  |  |  |
| 1 | Vitvi07g02289\_t001 |  | \| \| |  |  |  |  |  |  |  |  |  |  |
| 1 | Vitvi07g02290\_t001 |  | \| \| |  |  |  |  |  |  |  |  |  |  |
| 1 | Vitvi07g02291\_t001 |  | \| \| |  |  |  |  |  |  |  |  |  |  |
| 1 | Vitvi07g04165\_t001 |  | \| \| |  |  |  |  |  |  |  |  |  |  |
| 1 | Vitvi07g00661\_t001 |  | \| \| |  |  |  |  |  |  |  |  |  |  |
| 1 | Vitvi07g00664\_t001 |  | \| \| |  |  |  |  |  |  |  |  |  |  |
| 1 | Vitvi07g02294\_t001 |  | \| \| |  |  |  |  |  |  |  |  |  |  |
| 1 | Vitvi07g00665\_t001 |  | \| \| |  |  |  |  |  |  |  |  |  |  |
| 1 | Vitvi07g00666\_t001 |  | \| \| |  |  |  |  |  |  |  |  |  |  |
| 1 | Vitvi07g00667\_t001 |  | \| \| |  |  |  |  |  |  |  |  |  |  |
| 1 | Vitvi07g00668\_t001 |  | \| \| |  |  |  |  |  |  |  |  |  |  |
| 1 | Vitvi07g04166\_t001 |  | Vitvi05g04210\_t001 |  |  |  |  |  |  |  |  |  |  |
| 1 | Vitvi07g00672\_t001 |  | \| \| |  |  |  |  |  |  |  |  |  |  |
| 1 | Vitvi07g00674\_t001 |  | \| \| |  |  |  |  |  |  |  |  |  |  |
| 1 | Vitvi07g00675\_t001 |  | Vitvi05g00821\_t001 |  |  |  |  |  |  |  |  |  |  |
| 1 | Vitvi07g02295\_t001 |  | \| \| |  |  |  |  |  |  |  |  |  |  |
| 1 | Vitvi07g00676\_t001 |  | Vitvi05g00828\_t001 |  |  |  |  |  |  |  |  |  |  |
| 1 | Vitvi07g00677\_t001 |  | Vitvi05g00829\_t001 |  |  |  |  |  |  |  |  |  |  |
| 1 | Vitvi07g00678\_t001 |  | \| \| |  |  |  |  |  |  |  |  |  |  |
| 1 | Vitvi07g00679\_t001 |  | \| \| |  |  |  |  |  |  |  |  |  |  |
| 1 | Vitvi07g00680\_t001 |  | \| \| |  |  |  |  |  |  |  |  |  |  |
| 1 | Vitvi07g00682\_t001 |  | \| \| |  |  |  |  |  |  |  |  |  |  |
| 1 | Vitvi07g00683\_t001 |  | Vitvi05g00835\_t002 |  |  |  |  |  |  |  |  |  |  |
| 1 | Vitvi07g02298\_t001 |  | \| \| |  |  |  |  |  |  |  |  |  |  |
| 1 | Vitvi07g04167\_t001 |  | \| \| |  |  |  |  |  |  |  |  |  |  |
| 1 | Vitvi07g00684\_t001 |  | Vitvi05g00836\_t002 |  |  |  |  |  |  |  |  |  |  |
| 1 | Vitvi07g00685\_t001 |  | \| \| |  |  |  |  |  |  |  |  |  |  |
| 1 | Vitvi07g00686\_t001 |  | \| \| |  |  |  |  |  |  |  |  |  |  |
| 1 | Vitvi07g04168\_t001 |  | \| \| |  |  |  |  |  |  |  |  |  |  |
| 1 | Vitvi07g00687\_t001 |  | Vitvi05g00838\_t001 |  |  |  |  |  |  |  |  |  |  |
| 0 | Vitvi07g00688\_t001 |  |  |  |  |  |  |  |  |  |  |  |  |
| 0 | Vitvi07g00690\_t001 |  |  |  |  |  |  |  |  |  |  |  |  |
| 0 | Vitvi07g02300\_t001 |  |  |  |  |  |  |  |  |  |  |  |  |
| 0 | Vitvi07g00691\_t001 |  |  |  |  |  |  |  |  |  |  |  |  |
| 0 | Vitvi07g00693\_t001 |  |  |  |  |  |  |  |  |  |  |  |  |
| 0 | Vitvi07g04169\_t001 |  |  |  |  |  |  |  |  |  |  |  |  |
| 0 | Vitvi07g04170\_t001 |  |  |  |  |  |  |  |  |  |  |  |  |
| 0 | Vitvi07g00695\_t001 |  |  |  |  |  |  |  |  |  |  |  |  |
| 0 | Vitvi07g02301\_t001 |  |  |  |  |  |  |  |  |  |  |  |  |
| 0 | Vitvi07g00696\_t001 |  |  |  |  |  |  |  |  |  |  |  |  |
| 0 | Vitvi07g00697\_t001 |  |  |  |  |  |  |  |  |  |  |  |  |
| 0 | Vitvi07g00698\_t001 |  |  |  |  |  |  |  |  |  |  |  |  |
| 0 | Vitvi07g00701\_t001 |  |  |  |  |  |  |  |  |  |  |  |  |
| 0 | Vitvi07g04171\_t001 |  |  |  |  |  |  |  |  |  |  |  |  |
| 0 | Vitvi07g00703\_t001 |  |  |  |  |  |  |  |  |  |  |  |  |
| 0 | Vitvi07g00704\_t001 |  |  |  |  |  |  |  |  |  |  |  |  |
| 0 | Vitvi07g00705\_t001 |  |  |  |  |  |  |  |  |  |  |  |  |
| 0 | Vitvi07g04172\_t001 |  |  |  |  |  |  |  |  |  |  |  |  |
| 0 | Vitvi07g00706\_t001 |  |  |  |  |  |  |  |  |  |  |  |  |
| 0 | Vitvi07g00707\_t001 |  |  |  |  |  |  |  |  |  |  |  |  |
| 0 | Vitvi07g04173\_t001 |  |  |  |  |  |  |  |  |  |  |  |  |
| 0 | Vitvi07g00709\_t001 |  |  |  |  |  |  |  |  |  |  |  |  |
| 0 | Vitvi07g00710\_t001 |  |  |  |  |  |  |  |  |  |  |  |  |
| 0 | Vitvi07g00711\_t001 |  |  |  |  |  |  |  |  |  |  |  |  |
| 0 | Vitvi07g02304\_t001 |  |  |  |  |  |  |  |  |  |  |  |  |
| 0 | Vitvi07g02305\_t001 |  |  |  |  |  |  |  |  |  |  |  |  |
| 0 | Vitvi07g00716\_t001 |  |  |  |  |  |  |  |  |  |  |  |  |
| 0 | Vitvi07g02306\_t001 |  |  |  |  |  |  |  |  |  |  |  |  |
| 0 | Vitvi07g04174\_t001 |  |  |  |  |  |  |  |  |  |  |  |  |
| 0 | Vitvi07g00719\_t001 |  |  |  |  |  |  |  |  |  |  |  |  |
| 0 | Vitvi07g00720\_t001 |  |  |  |  |  |  |  |  |  |  |  |  |
| 0 | Vitvi07g00721\_t001 |  |  |  |  |  |  |  |  |  |  |  |  |
| 0 | Vitvi07g00722\_t001 |  |  |  |  |  |  |  |  |  |  |  |  |
| 0 | Vitvi07g00724\_t001 |  |  |  |  |  |  |  |  |  |  |  |  |
| 0 | Vitvi07g00726\_t001 |  |  |  |  |  |  |  |  |  |  |  |  |
| 0 | Vitvi07g04175\_t001 |  |  |  |  |  |  |  |  |  |  |  |  |
| 0 | Vitvi07g04176\_t001 |  |  |  |  |  |  |  |  |  |  |  |  |
| 0 | Vitvi07g00727\_t001 |  |  |  |  |  |  |  |  |  |  |  |  |
| 0 | Vitvi07g00730\_t001 |  |  |  |  |  |  |  |  |  |  |  |  |
| 0 | Vitvi07g00731\_t001 |  |  |  |  |  |  |  |  |  |  |  |  |
| 0 | Vitvi07g00732\_t001 |  |  |  |  |  |  |  |  |  |  |  |  |
| 0 | Vitvi07g00733\_t001 |  |  |  |  |  |  |  |  |  |  |  |  |
| 0 | Vitvi07g00734\_t001 |  |  |  |  |  |  |  |  |  |  |  |  |
| 0 | Vitvi07g00735\_t001 |  |  |  |  |  |  |  |  |  |  |  |  |
| 0 | Vitvi07g00736\_t001 |  |  |  |  |  |  |  |  |  |  |  |  |
| 0 | Vitvi07g00737\_t001 |  |  |  |  |  |  |  |  |  |  |  |  |
| 0 | Vitvi07g04177\_t001 |  |  |  |  |  |  |  |  |  |  |  |  |
| 0 | Vitvi07g02309\_t001 |  |  |  |  |  |  |  |  |  |  |  |  |
| 0 | Vitvi07g00738\_t001 |  |  |  |  |  |  |  |  |  |  |  |  |
| 0 | Vitvi07g00739\_t001 |  |  |  |  |  |  |  |  |  |  |  |  |
| 0 | Vitvi07g00740\_t001 |  |  |  |  |  |  |  |  |  |  |  |  |
| 0 | Vitvi07g00741\_t001 |  |  |  |  |  |  |  |  |  |  |  |  |
| 0 | Vitvi07g00742\_t001 |  |  |  |  |  |  |  |  |  |  |  |  |
| 0 | Vitvi07g04178\_t001 |  |  |  |  |  |  |  |  |  |  |  |  |
| 0 | Vitvi07g04179\_t001 |  |  |  |  |  |  |  |  |  |  |  |  |
| 0 | Vitvi07g04180\_t001 |  |  |  |  |  |  |  |  |  |  |  |  |
| 0 | Vitvi07g00743\_t001 |  |  |  |  |  |  |  |  |  |  |  |  |
| 0 | Vitvi07g00744\_t001 |  |  |  |  |  |  |  |  |  |  |  |  |
| 0 | Vitvi07g00745\_t001 |  |  |  |  |  |  |  |  |  |  |  |  |
| 0 | Vitvi07g04181\_t001 |  |  |  |  |  |  |  |  |  |  |  |  |
| 0 | Vitvi07g04182\_t001 |  |  |  |  |  |  |  |  |  |  |  |  |
| 0 | Vitvi07g04183\_t001 |  |  |  |  |  |  |  |  |  |  |  |  |
| 0 | Vitvi07g04184\_t001 |  |  |  |  |  |  |  |  |  |  |  |  |
| 0 | Vitvi07g04185\_t001 |  |  |  |  |  |  |  |  |  |  |  |  |
| 0 | Vitvi07g04186\_t001 |  |  |  |  |  |  |  |  |  |  |  |  |
| 0 | Vitvi07g04187\_t001 |  |  |  |  |  |  |  |  |  |  |  |  |
| 0 | Vitvi07g04188\_t001 |  |  |  |  |  |  |  |  |  |  |  |  |
| 0 | Vitvi07g04189\_t001 |  |  |  |  |  |  |  |  |  |  |  |  |
| 0 | Vitvi07g04190\_t001 |  |  |  |  |  |  |  |  |  |  |  |  |
| 0 | Vitvi07g04191\_t001 |  |  |  |  |  |  |  |  |  |  |  |  |
| 0 | Vitvi07g00762\_t001 |  |  |  |  |  |  |  |  |  |  |  |  |
| 0 | Vitvi07g02313\_t001 |  |  |  |  |  |  |  |  |  |  |  |  |
| 0 | Vitvi07g00765\_t001 |  |  |  |  |  |  |  |  |  |  |  |  |
| 0 | Vitvi07g02315\_t001 |  |  |  |  |  |  |  |  |  |  |  |  |
| 0 | Vitvi07g00767\_t001 |  |  |  |  |  |  |  |  |  |  |  |  |
| 0 | Vitvi07g02317\_t001 |  |  |  |  |  |  |  |  |  |  |  |  |
| 0 | Vitvi07g02318\_t001 |  |  |  |  |  |  |  |  |  |  |  |  |
| 0 | Vitvi07g04192\_t001 |  |  |  |  |  |  |  |  |  |  |  |  |
| 0 | Vitvi07g04193\_t001 |  |  |  |  |  |  |  |  |  |  |  |  |
| 0 | Vitvi07g04194\_t001 |  |  |  |  |  |  |  |  |  |  |  |  |
| 0 | Vitvi07g04195\_t001 |  |  |  |  |  |  |  |  |  |  |  |  |
| 0 | Vitvi07g04196\_t001 |  |  |  |  |  |  |  |  |  |  |  |  |
| 0 | Vitvi07g04197\_t001 |  |  |  |  |  |  |  |  |  |  |  |  |
| 0 | Vitvi07g02320\_t001 |  |  |  |  |  |  |  |  |  |  |  |  |
| 0 | Vitvi07g00773\_t001 |  |  |  |  |  |  |  |  |  |  |  |  |
| 0 | Vitvi07g02321\_t001 |  |  |  |  |  |  |  |  |  |  |  |  |
| 0 | Vitvi07g04198\_t001 |  |  |  |  |  |  |  |  |  |  |  |  |
| 0 | Vitvi07g02323\_t001 |  |  |  |  |  |  |  |  |  |  |  |  |
| 0 | Vitvi07g02326\_t001 |  |  |  |  |  |  |  |  |  |  |  |  |
| 0 | Vitvi07g00780\_t001 |  |  |  |  |  |  |  |  |  |  |  |  |
| 0 | Vitvi07g04199\_t001 |  |  |  |  |  |  |  |  |  |  |  |  |
| 0 | Vitvi07g00781\_t001 |  |  |  |  |  |  |  |  |  |  |  |  |
| 0 | Vitvi07g02330\_t001 |  |  |  |  |  |  |  |  |  |  |  |  |
| 0 | Vitvi07g04200\_t001 |  |  |  |  |  |  |  |  |  |  |  |  |
| 0 | Vitvi07g04201\_t001 |  |  |  |  |  |  |  |  |  |  |  |  |
| 0 | Vitvi07g04202\_t001 |  |  |  |  |  |  |  |  |  |  |  |  |
| 0 | Vitvi07g02334\_t001 |  |  |  |  |  |  |  |  |  |  |  |  |
| 0 | Vitvi07g00786\_t001 |  |  |  |  |  |  |  |  |  |  |  |  |
| 0 | Vitvi07g00787\_t001 |  |  |  |  |  |  |  |  |  |  |  |  |
| 0 | Vitvi07g00790\_t001 |  |  |  |  |  |  |  |  |  |  |  |  |
| 0 | Vitvi07g02337\_t001 |  |  |  |  |  |  |  |  |  |  |  |  |
| 0 | Vitvi07g04203\_t001 |  |  |  |  |  |  |  |  |  |  |  |  |
| 0 | Vitvi07g04204\_t001 |  |  |  |  |  |  |  |  |  |  |  |  |
| 0 | Vitvi07g02342\_t001 |  |  |  |  |  |  |  |  |  |  |  |  |
| 0 | Vitvi07g00792\_t001 |  |  |  |  |  |  |  |  |  |  |  |  |
| 0 | Vitvi07g00795\_t001 |  |  |  |  |  |  |  |  |  |  |  |  |
| 0 | Vitvi07g00796\_t001 |  |  |  |  |  |  |  |  |  |  |  |  |
| 0 | Vitvi07g00801\_t003 |  |  |  |  |  |  |  |  |  |  |  |  |
| 0 | Vitvi07g02345\_t001 |  |  |  |  |  |  |  |  |  |  |  |  |
| 0 | Vitvi07g04205\_t001 |  |  |  |  |  |  |  |  |  |  |  |  |
| 0 | Vitvi07g00803\_t001 |  |  |  |  |  |  |  |  |  |  |  |  |
| 0 | Vitvi07g00806\_t001 |  |  |  |  |  |  |  |  |  |  |  |  |
| 0 | Vitvi07g00807\_t001 |  |  |  |  |  |  |  |  |  |  |  |  |
| 0 | Vitvi07g04206\_t001 |  |  |  |  |  |  |  |  |  |  |  |  |
| 0 | Vitvi07g00809\_t001 |  |  |  |  |  |  |  |  |  |  |  |  |
| 0 | Vitvi07g04207\_t001 |  |  |  |  |  |  |  |  |  |  |  |  |
| 0 | Vitvi07g04208\_t001 |  |  |  |  |  |  |  |  |  |  |  |  |
| 0 | Vitvi07g00810\_t001 |  |  |  |  |  |  |  |  |  |  |  |  |
| 0 | Vitvi07g00812\_t001 |  |  |  |  |  |  |  |  |  |  |  |  |
| 0 | Vitvi07g04209\_t001 |  |  |  |  |  |  |  |  |  |  |  |  |
| 0 | Vitvi07g04210\_t001 |  |  |  |  |  |  |  |  |  |  |  |  |
| 0 | Vitvi07g00814\_t001 |  |  |  |  |  |  |  |  |  |  |  |  |
| 0 | Vitvi07g02346\_t001 |  |  |  |  |  |  |  |  |  |  |  |  |
| 0 | Vitvi07g02347\_t001 |  |  |  |  |  |  |  |  |  |  |  |  |
| 0 | Vitvi07g00822\_t001 |  |  |  |  |  |  |  |  |  |  |  |  |
| 0 | Vitvi07g00824\_t001 |  |  |  |  |  |  |  |  |  |  |  |  |
| 0 | Vitvi07g00825\_t001 |  |  |  |  |  |  |  |  |  |  |  |  |
| 0 | Vitvi07g04211\_t001 |  |  |  |  |  |  |  |  |  |  |  |  |
| 0 | Vitvi07g00827\_t001 |  |  |  |  |  |  |  |  |  |  |  |  |
| 0 | Vitvi07g04212\_t001 |  |  |  |  |  |  |  |  |  |  |  |  |
| 0 | Vitvi07g00830\_t001 |  |  |  |  |  |  |  |  |  |  |  |  |
| 0 | Vitvi07g00831\_t001 |  |  |  |  |  |  |  |  |  |  |  |  |
| 0 | Vitvi07g04213\_t001 |  |  |  |  |  |  |  |  |  |  |  |  |
| 0 | Vitvi07g04214\_t001 |  |  |  |  |  |  |  |  |  |  |  |  |
| 0 | Vitvi07g00837\_t001 |  |  |  |  |  |  |  |  |  |  |  |  |
| 0 | Vitvi07g04215\_t001 |  |  |  |  |  |  |  |  |  |  |  |  |
| 0 | Vitvi07g04216\_t001 |  |  |  |  |  |  |  |  |  |  |  |  |
| 0 | Vitvi07g04217\_t001 |  |  |  |  |  |  |  |  |  |  |  |  |
| 0 | Vitvi07g04218\_t001 |  |  |  |  |  |  |  |  |  |  |  |  |
| 0 | Vitvi07g00846\_t001 |  |  |  |  |  |  |  |  |  |  |  |  |
| 0 | Vitvi07g04219\_t001 |  |  |  |  |  |  |  |  |  |  |  |  |
| 0 | Vitvi07g04220\_t001 |  |  |  |  |  |  |  |  |  |  |  |  |
| 0 | Vitvi07g04221\_t001 |  |  |  |  |  |  |  |  |  |  |  |  |
| 0 | Vitvi07g02355\_t001 |  |  |  |  |  |  |  |  |  |  |  |  |
| 0 | Vitvi07g00850\_t001 |  |  |  |  |  |  |  |  |  |  |  |  |
| 0 | Vitvi07g00853\_t001 |  |  |  |  |  |  |  |  |  |  |  |  |
| 0 | Vitvi07g04222\_t001 |  |  |  |  |  |  |  |  |  |  |  |  |
| 0 | Vitvi07g04223\_t001 |  |  |  |  |  |  |  |  |  |  |  |  |
| 0 | Vitvi07g00857\_t001 |  |  |  |  |  |  |  |  |  |  |  |  |
| 0 | Vitvi07g02357\_t001 |  |  |  |  |  |  |  |  |  |  |  |  |
| 0 | Vitvi07g04224\_t001 |  |  |  |  |  |  |  |  |  |  |  |  |
| 0 | Vitvi07g04225\_t001 |  |  |  |  |  |  |  |  |  |  |  |  |
| 0 | Vitvi07g00867\_t001 |  |  |  |  |  |  |  |  |  |  |  |  |
| 0 | Vitvi07g00869\_t001 |  |  |  |  |  |  |  |  |  |  |  |  |
| 0 | Vitvi07g04226\_t001 |  |  |  |  |  |  |  |  |  |  |  |  |
| 0 | Vitvi07g04227\_t001 |  |  |  |  |  |  |  |  |  |  |  |  |
| 0 | Vitvi07g04228\_t001 |  |  |  |  |  |  |  |  |  |  |  |  |
| 0 | Vitvi07g00871\_t001 |  |  |  |  |  |  |  |  |  |  |  |  |
| 0 | Vitvi07g00874\_t001 |  |  |  |  |  |  |  |  |  |  |  |  |
| 0 | Vitvi07g04229\_t001 |  |  |  |  |  |  |  |  |  |  |  |  |
| 0 | Vitvi07g04230\_t001 |  |  |  |  |  |  |  |  |  |  |  |  |
| 0 | Vitvi07g04231\_t001 |  |  |  |  |  |  |  |  |  |  |  |  |
| 0 | Vitvi07g02360\_t001 |  |  |  |  |  |  |  |  |  |  |  |  |
| 0 | Vitvi07g04232\_t001 |  |  |  |  |  |  |  |  |  |  |  |  |
| 0 | Vitvi07g04233\_t001 |  |  |  |  |  |  |  |  |  |  |  |  |
| 0 | Vitvi07g02362\_t001 |  |  |  |  |  |  |  |  |  |  |  |  |
| 0 | Vitvi07g00879\_t001 |  |  |  |  |  |  |  |  |  |  |  |  |
| 0 | Vitvi07g02363\_t001 |  |  |  |  |  |  |  |  |  |  |  |  |
| 0 | Vitvi07g04234\_t001 |  |  |  |  |  |  |  |  |  |  |  |  |
| 0 | Vitvi07g00883\_t001 |  |  |  |  |  |  |  |  |  |  |  |  |
| 0 | Vitvi07g00884\_t001 |  |  |  |  |  |  |  |  |  |  |  |  |
| 0 | Vitvi07g02364\_t001 |  |  |  |  |  |  |  |  |  |  |  |  |
| 0 | Vitvi07g00887\_t001 |  |  |  |  |  |  |  |  |  |  |  |  |
| 0 | Vitvi07g04235\_t001 |  |  |  |  |  |  |  |  |  |  |  |  |
| 0 | Vitvi07g00889\_t001 |  |  |  |  |  |  |  |  |  |  |  |  |
| 0 | Vitvi07g00890\_t001 |  |  |  |  |  |  |  |  |  |  |  |  |
| 0 | Vitvi07g00892\_t001 |  |  |  |  |  |  |  |  |  |  |  |  |
| 0 | Vitvi07g00894\_t001 |  |  |  |  |  |  |  |  |  |  |  |  |
| 0 | Vitvi07g04236\_t001 |  |  |  |  |  |  |  |  |  |  |  |  |
| 0 | Vitvi07g04237\_t001 |  |  |  |  |  |  |  |  |  |  |  |  |
| 0 | Vitvi07g00899\_t001 |  |  |  |  |  |  |  |  |  |  |  |  |
| 0 | Vitvi07g04238\_t001 |  |  |  |  |  |  |  |  |  |  |  |  |
| 0 | Vitvi07g00903\_t001 |  |  |  |  |  |  |  |  |  |  |  |  |
| 0 | Vitvi07g04239\_t001 |  |  |  |  |  |  |  |  |  |  |  |  |
| 0 | Vitvi07g00905\_t001 |  |  |  |  |  |  |  |  |  |  |  |  |
| 0 | Vitvi07g00906\_t001 |  |  |  |  |  |  |  |  |  |  |  |  |
| 0 | Vitvi07g00907\_t001 |  |  |  |  |  |  |  |  |  |  |  |  |
| 0 | Vitvi07g02371\_t001 |  |  |  |  |  |  |  |  |  |  |  |  |
| 0 | Vitvi07g00908\_t001 |  |  |  |  |  |  |  |  |  |  |  |  |
| 0 | Vitvi07g00910\_t001 |  |  |  |  |  |  |  |  |  |  |  |  |
| 0 | Vitvi07g04240\_t001 |  |  |  |  |  |  |  |  |  |  |  |  |
| 0 | Vitvi07g00912\_t001 |  |  |  |  |  |  |  |  |  |  |  |  |
| 0 | Vitvi07g02374\_t001 |  |  |  |  |  |  |  |  |  |  |  |  |
| 0 | Vitvi07g04241\_t001 |  |  |  |  |  |  |  |  |  |  |  |  |
| 0 | Vitvi07g04242\_t001 |  |  |  |  |  |  |  |  |  |  |  |  |
| 0 | Vitvi07g00914\_t002 |  |  |  |  |  |  |  |  |  |  |  |  |
| 0 | Vitvi07g04243\_t001 |  |  |  |  |  |  |  |  |  |  |  |  |
| 0 | Vitvi07g00916\_t001 |  |  |  |  |  |  |  |  |  |  |  |  |
| 0 | Vitvi07g00918\_t001 |  |  |  |  |  |  |  |  |  |  |  |  |
| 0 | Vitvi07g00919\_t001 |  |  |  |  |  |  |  |  |  |  |  |  |
| 0 | Vitvi07g04244\_t001 |  |  |  |  |  |  |  |  |  |  |  |  |
| 0 | Vitvi07g00921\_t001 |  |  |  |  |  |  |  |  |  |  |  |  |
| 0 | Vitvi07g00923\_t001 |  |  |  |  |  |  |  |  |  |  |  |  |
| 0 | Vitvi07g00924\_t001 |  |  |  |  |  |  |  |  |  |  |  |  |
| 0 | Vitvi07g04245\_t001 |  |  |  |  |  |  |  |  |  |  |  |  |
| 0 | Vitvi07g00930\_t002 |  |  |  |  |  |  |  |  |  |  |  |  |
| 0 | Vitvi07g04246\_t001 |  |  |  |  |  |  |  |  |  |  |  |  |
| 0 | Vitvi07g04247\_t001 |  |  |  |  |  |  |  |  |  |  |  |  |
| 0 | Vitvi07g04248\_t001 |  |  |  |  |  |  |  |  |  |  |  |  |
| 0 | Vitvi07g04249\_t001 |  |  |  |  |  |  |  |  |  |  |  |  |
| 0 | Vitvi07g04250\_t001 |  |  |  |  |  |  |  |  |  |  |  |  |
| 0 | Vitvi07g02071\_t001 |  |  |  |  |  |  |  |  |  |  |  |  |
| 0 | Vitvi07g00933\_t001 |  |  |  |  |  |  |  |  |  |  |  |  |
| 0 | Vitvi07g00934\_t001 |  |  |  |  |  |  |  |  |  |  |  |  |
| 0 | Vitvi07g00936\_t001 |  |  |  |  |  |  |  |  |  |  |  |  |
| 0 | Vitvi07g04251\_t001 |  |  |  |  |  |  |  |  |  |  |  |  |
| 0 | Vitvi07g04252\_t001 |  |  |  |  |  |  |  |  |  |  |  |  |
| 0 | Vitvi07g00939\_t001 |  |  |  |  |  |  |  |  |  |  |  |  |
| 0 | Vitvi07g02382\_t001 |  |  |  |  |  |  |  |  |  |  |  |  |
| 0 | Vitvi07g00940\_t001 |  |  |  |  |  |  |  |  |  |  |  |  |
| 0 | Vitvi07g00941\_t001 |  |  |  |  |  |  |  |  |  |  |  |  |
| 0 | Vitvi07g00943\_t002 |  |  |  |  |  |  |  |  |  |  |  |  |
| 0 | Vitvi07g04253\_t001 |  |  |  |  |  |  |  |  |  |  |  |  |
| 0 | Vitvi07g00949\_t001 |  |  |  |  |  |  |  |  |  |  |  |  |
| 0 | Vitvi07g02734\_t001 |  |  |  |  |  |  |  |  |  |  |  |  |
| 0 | Vitvi07g04254\_t001 |  |  |  |  |  |  |  |  |  |  |  |  |
| 0 | Vitvi07g04255\_t001 |  |  |  |  |  |  |  |  |  |  |  |  |
| 0 | Vitvi07g00955\_t001 |  |  |  |  |  |  |  |  |  |  |  |  |
| 0 | Vitvi07g04256\_t001 |  |  |  |  |  |  |  |  |  |  |  |  |
| 0 | Vitvi07g00960\_t001 |  |  |  |  |  |  |  |  |  |  |  |  |
| 0 | Vitvi07g02730\_t001 |  |  |  |  |  |  |  |  |  |  |  |  |
| 0 | Vitvi07g04257\_t001 |  |  |  |  |  |  |  |  |  |  |  |  |
| 0 | Vitvi07g04258\_t001 |  |  |  |  |  |  |  |  |  |  |  |  |
| 0 | Vitvi07g02386\_t001 |  |  |  |  |  |  |  |  |  |  |  |  |
| 0 | Vitvi07g04259\_t001 |  |  |  |  |  |  |  |  |  |  |  |  |
| 0 | Vitvi07g02389\_t001 |  |  |  |  |  |  |  |  |  |  |  |  |
| 0 | Vitvi07g04260\_t001 |  |  |  |  |  |  |  |  |  |  |  |  |
| 0 | Vitvi07g04261\_t001 |  |  |  |  |  |  |  |  |  |  |  |  |
| 0 | Vitvi07g04262\_t001 |  |  |  |  |  |  |  |  |  |  |  |  |
| 0 | Vitvi07g00974\_t001 |  |  |  |  |  |  |  |  |  |  |  |  |
| 0 | Vitvi07g00978\_t001 |  |  |  |  |  |  |  |  |  |  |  |  |
| 0 | Vitvi07g04263\_t001 |  |  |  |  |  |  |  |  |  |  |  |  |
| 0 | Vitvi07g04264\_t001 |  |  |  |  |  |  |  |  |  |  |  |  |
| 0 | Vitvi07g00980\_t001 |  |  |  |  |  |  |  |  |  |  |  |  |
| 0 | Vitvi07g04265\_t001 |  |  |  |  |  |  |  |  |  |  |  |  |
| 0 | Vitvi07g04266\_t001 |  |  |  |  |  |  |  |  |  |  |  |  |
| 0 | Vitvi07g00983\_t001 |  |  |  |  |  |  |  |  |  |  |  |  |
| 0 | Vitvi07g04267\_t001 |  |  |  |  |  |  |  |  |  |  |  |  |
| 0 | Vitvi07g00984\_t001 |  |  |  |  |  |  |  |  |  |  |  |  |
| 0 | Vitvi07g04268\_t001 |  |  |  |  |  |  |  |  |  |  |  |  |
| 0 | Vitvi07g04269\_t001 |  |  |  |  |  |  |  |  |  |  |  |  |
| 0 | Vitvi07g00988\_t001 |  |  |  |  |  |  |  |  |  |  |  |  |
| 0 | Vitvi07g02397\_t001 |  |  |  |  |  |  |  |  |  |  |  |  |
| 0 | Vitvi07g04270\_t001 |  |  |  |  |  |  |  |  |  |  |  |  |
| 0 | Vitvi07g04271\_t001 |  |  |  |  |  |  |  |  |  |  |  |  |
| 0 | Vitvi07g01004\_t001 |  |  |  |  |  |  |  |  |  |  |  |  |
| 0 | Vitvi07g02399\_t001 |  |  |  |  |  |  |  |  |  |  |  |  |
| 0 | Vitvi07g02402\_t001 |  |  |  |  |  |  |  |  |  |  |  |  |
| 0 | Vitvi07g02403\_t001 |  |  |  |  |  |  |  |  |  |  |  |  |
| 0 | Vitvi07g02405\_t001 |  |  |  |  |  |  |  |  |  |  |  |  |
| 0 | Vitvi07g04272\_t001 |  |  |  |  |  |  |  |  |  |  |  |  |
| 0 | Vitvi07g01017\_t001 |  |  |  |  |  |  |  |  |  |  |  |  |
| 0 | Vitvi07g02410\_t001 |  |  |  |  |  |  |  |  |  |  |  |  |
| 0 | Vitvi07g02412\_t001 |  |  |  |  |  |  |  |  |  |  |  |  |
| 0 | Vitvi07g01026\_t001 |  |  |  |  |  |  |  |  |  |  |  |  |
| 0 | Vitvi07g04273\_t001 |  |  |  |  |  |  |  |  |  |  |  |  |
| 0 | Vitvi07g04274\_t001 |  |  |  |  |  |  |  |  |  |  |  |  |
| 0 | Vitvi07g04275\_t001 |  |  |  |  |  |  |  |  |  |  |  |  |
| 0 | Vitvi07g02414\_t001 |  |  |  |  |  |  |  |  |  |  |  |  |
| 0 | Vitvi07g02415\_t001 |  |  |  |  |  |  |  |  |  |  |  |  |
| 0 | Vitvi07g04276\_t001 |  |  |  |  |  |  |  |  |  |  |  |  |
| 0 | Vitvi07g04277\_t001 |  |  |  |  |  |  |  |  |  |  |  |  |
| 0 | Vitvi07g01032\_t001 |  |  |  |  |  |  |  |  |  |  |  |  |
| 0 | Vitvi07g01033\_t001 |  |  |  |  |  |  |  |  |  |  |  |  |
| 0 | Vitvi07g01038\_t001 |  |  |  |  |  |  |  |  |  |  |  |  |
| 0 | Vitvi07g04278\_t001 |  |  |  |  |  |  |  |  |  |  |  |  |
| 0 | Vitvi07g01039\_t001 |  |  |  |  |  |  |  |  |  |  |  |  |
| 0 | Vitvi07g01041\_t001 |  |  |  |  |  |  |  |  |  |  |  |  |
| 0 | Vitvi07g01042\_t001 |  |  |  |  |  |  |  |  |  |  |  |  |
| 0 | Vitvi07g04279\_t001 |  |  |  |  |  |  |  |  |  |  |  |  |
| 0 | Vitvi07g01045\_t001 |  |  |  |  |  |  |  |  |  |  |  |  |
| 0 | Vitvi07g04280\_t001 |  |  |  |  |  |  |  |  |  |  |  |  |
| 0 | Vitvi07g02418\_t001 |  |  |  |  |  |  |  |  |  |  |  |  |
| 0 | Vitvi07g01050\_t001 |  |  |  |  |  |  |  |  |  |  |  |  |
| 0 | Vitvi07g04281\_t001 |  |  |  |  |  |  |  |  |  |  |  |  |
| 0 | Vitvi07g04282\_t001 |  |  |  |  |  |  |  |  |  |  |  |  |
| 0 | Vitvi07g04283\_t001 |  |  |  |  |  |  |  |  |  |  |  |  |
| 0 | Vitvi07g04284\_t001 |  |  |  |  |  |  |  |  |  |  |  |  |
| 0 | Vitvi07g04285\_t001 |  |  |  |  |  |  |  |  |  |  |  |  |
| 0 | Vitvi07g02878\_t001 |  |  |  |  |  |  |  |  |  |  |  |  |
| 0 | Vitvi07g02881\_t001 |  |  |  |  |  |  |  |  |  |  |  |  |
| 0 | Vitvi07g04286\_t001 |  |  |  |  |  |  |  |  |  |  |  |  |
| 0 | Vitvi07g02882\_t001 |  |  |  |  |  |  |  |  |  |  |  |  |
| 0 | Vitvi07g02883\_t001 |  |  |  |  |  |  |  |  |  |  |  |  |
| 0 | Vitvi07g02885\_t001 |  |  |  |  |  |  |  |  |  |  |  |  |
| 0 | Vitvi07g02886\_t001 |  |  |  |  |  |  |  |  |  |  |  |  |
| 0 | Vitvi07g04287\_t001 |  |  |  |  |  |  |  |  |  |  |  |  |
| 0 | Vitvi07g02888\_t001 |  |  |  |  |  |  |  |  |  |  |  |  |
| 0 | Vitvi07g04288\_t001 |  |  |  |  |  |  |  |  |  |  |  |  |
| 0 | Vitvi07g04289\_t001 |  |  |  |  |  |  |  |  |  |  |  |  |
| 0 | Vitvi07g04290\_t001 |  |  |  |  |  |  |  |  |  |  |  |  |
| 0 | Vitvi07g04291\_t001 |  |  |  |  |  |  |  |  |  |  |  |  |
| 0 | Vitvi07g02745\_t001 |  |  |  |  |  |  |  |  |  |  |  |  |
| 0 | Vitvi07g02751\_t001 |  |  |  |  |  |  |  |  |  |  |  |  |
| 0 | Vitvi07g02752\_t001 |  |  |  |  |  |  |  |  |  |  |  |  |
| 0 | Vitvi07g02753\_t001 |  |  |  |  |  |  |  |  |  |  |  |  |
| 0 | Vitvi07g02755\_t001 |  |  |  |  |  |  |  |  |  |  |  |  |
| 0 | Vitvi07g04292\_t001 |  |  |  |  |  |  |  |  |  |  |  |  |
| 0 | Vitvi07g02756\_t001 |  |  |  |  |  |  |  |  |  |  |  |  |
| 0 | Vitvi07g02757\_t001 |  |  |  |  |  |  |  |  |  |  |  |  |
| 0 | Vitvi07g02759\_t001 |  |  |  |  |  |  |  |  |  |  |  |  |
| 0 | Vitvi07g04293\_t001 |  |  |  |  |  |  |  |  |  |  |  |  |
| 0 | Vitvi07g04294\_t001 |  |  |  |  |  |  |  |  |  |  |  |  |
| 0 | Vitvi07g02767\_t001 |  |  |  |  |  |  |  |  |  |  |  |  |
| 0 | Vitvi07g02768\_t002 |  |  |  |  |  |  |  |  |  |  |  |  |
| 0 | Vitvi07g04295\_t001 |  |  |  |  |  |  |  |  |  |  |  |  |
| 0 | Vitvi07g02776\_t001 |  |  |  |  |  |  |  |  |  |  |  |  |
| 0 | Vitvi07g02787\_t001 |  |  |  |  |  |  |  |  |  |  |  |  |
| 0 | Vitvi07g02789\_t001 |  |  |  |  |  |  |  |  |  |  |  |  |
| 0 | Vitvi07g02791\_t001 |  |  |  |  |  |  |  |  |  |  |  |  |
| 0 | Vitvi07g04298\_t001 |  |  |  |  |  |  |  |  |  |  |  |  |
| 0 | Vitvi07g04299\_t001 |  |  |  |  |  |  |  |  |  |  |  |  |
| 0 | Vitvi07g04300\_t001 |  |  |  |  |  |  |  |  |  |  |  |  |
| 0 | Vitvi07g01054\_t001 |  |  |  |  |  |  |  |  |  |  |  |  |
| 0 | Vitvi07g04301\_t001 |  |  |  |  |  |  |  |  |  |  |  |  |
| 0 | Vitvi07g01055\_t001 |  |  |  |  |  |  |  |  |  |  |  |  |
| 0 | Vitvi07g04302\_t002 |  |  |  |  |  |  |  |  |  |  |  |  |
| 0 | Vitvi07g01057\_t001 |  |  |  |  |  |  |  |  |  |  |  |  |
| 0 | Vitvi07g04303\_t001 |  |  |  |  |  |  |  |  |  |  |  |  |
| 0 | Vitvi07g04304\_t001 |  |  |  |  |  |  |  |  |  |  |  |  |
| 0 | Vitvi07g04305\_t001 |  |  |  |  |  |  |  |  |  |  |  |  |
| 0 | Vitvi07g04306\_t002 |  |  |  |  |  |  |  |  |  |  |  |  |
| 0 | Vitvi07g01088\_t001 |  |  |  |  |  |  |  |  |  |  |  |  |
| 0 | Vitvi07g04307\_t002 |  |  |  |  |  |  |  |  |  |  |  |  |
| 0 | Vitvi07g01086\_t001 |  |  |  |  |  |  |  |  |  |  |  |  |
| 0 | Vitvi07g02433\_t001 |  |  |  |  |  |  |  |  |  |  |  |  |
| 0 | Vitvi07g02432\_t001 |  |  |  |  |  |  |  |  |  |  |  |  |
| 0 | Vitvi07g02431\_t001 |  |  |  |  |  |  |  |  |  |  |  |  |
| 0 | Vitvi07g04308\_t001 |  |  |  |  |  |  |  |  |  |  |  |  |
| 0 | Vitvi07g04309\_t001 |  |  |  |  |  |  |  |  |  |  |  |  |
| 0 | Vitvi07g01078\_t001 |  |  |  |  |  |  |  |  |  |  |  |  |
| 0 | Vitvi07g04310\_t001 |  |  |  |  |  |  |  |  |  |  |  |  |
| 0 | Vitvi07g04311\_t001 |  |  |  |  |  |  |  |  |  |  |  |  |
| 0 | Vitvi07g04312\_t001 |  |  |  |  |  |  |  |  |  |  |  |  |
| 0 | Vitvi07g04313\_t001 |  |  |  |  |  |  |  |  |  |  |  |  |
| 0 | Vitvi07g04314\_t001 |  |  |  |  |  |  |  |  |  |  |  |  |
| 0 | Vitvi07g01113\_t001 |  |  |  |  |  |  |  |  |  |  |  |  |
| 0 | Vitvi07g04315\_t001 |  |  |  |  |  |  |  |  |  |  |  |  |
| 0 | Vitvi07g01114\_t001 |  |  |  |  |  |  |  |  |  |  |  |  |
| 0 | Vitvi07g04316\_t001 |  |  |  |  |  |  |  |  |  |  |  |  |
| 0 | Vitvi07g01119\_t001 |  |  |  |  |  |  |  |  |  |  |  |  |
| 0 | Vitvi07g02441\_t001 |  |  |  |  |  |  |  |  |  |  |  |  |
| 0 | Vitvi07g01120\_t001 |  |  |  |  |  |  |  |  |  |  |  |  |
| 0 | Vitvi07g01124\_t001 |  |  |  |  |  |  |  |  |  |  |  |  |
| 0 | Vitvi07g04317\_t001 |  |  |  |  |  |  |  |  |  |  |  |  |
| 0 | Vitvi07g02902\_t001 |  |  |  |  |  |  |  |  |  |  |  |  |
| 0 | Vitvi07g04318\_t001 |  |  |  |  |  |  |  |  |  |  |  |  |
| 0 | Vitvi07g04319\_t001 |  |  |  |  |  |  |  |  |  |  |  |  |
| 0 | Vitvi07g02955\_t001 |  |  |  |  |  |  |  |  |  |  |  |  |
| 0 | Vitvi07g04320\_t001 |  |  |  |  |  |  |  |  |  |  |  |  |
| 0 | Vitvi07g04321\_t001 |  |  |  |  |  |  |  |  |  |  |  |  |
| 0 | Vitvi07g04322\_t001 |  |  |  |  |  |  |  |  |  |  |  |  |
| 0 | Vitvi07g02951\_t001 |  |  |  |  |  |  |  |  |  |  |  |  |
| 0 | Vitvi07g04323\_t001 |  |  |  |  |  |  |  |  |  |  |  |  |
| 0 | Vitvi07g02949\_t001 |  |  |  |  |  |  |  |  |  |  |  |  |
| 0 | Vitvi07g02948\_t001 |  |  |  |  |  |  |  |  |  |  |  |  |
| 0 | Vitvi07g04324\_t001 |  |  |  |  |  |  |  |  |  |  |  |  |
| 0 | Vitvi07g04325\_t001 |  |  |  |  |  |  |  |  |  |  |  |  |
| 0 | Vitvi07g02940\_t001 |  |  |  |  |  |  |  |  |  |  |  |  |
| 0 | Vitvi07g04326\_t001 |  |  |  |  |  |  |  |  |  |  |  |  |
| 0 | Vitvi07g04327\_t001 |  |  |  |  |  |  |  |  |  |  |  |  |
| 0 | Vitvi07g01110\_t003 |  |  |  |  |  |  |  |  |  |  |  |  |
| 0 | Vitvi07g04328\_t001 |  |  |  |  |  |  |  |  |  |  |  |  |
| 0 | Vitvi07g01109\_t001 |  |  |  |  |  |  |  |  |  |  |  |  |
| 0 | Vitvi07g01108\_t001 |  |  |  |  |  |  |  |  |  |  |  |  |
| 0 | Vitvi07g04329\_t001 |  |  |  |  |  |  |  |  |  |  |  |  |
| 0 | Vitvi07g01104\_t001 |  |  |  |  |  |  |  |  |  |  |  |  |
| 0 | Vitvi07g04330\_t001 |  |  |  |  |  |  |  |  |  |  |  |  |
| 0 | Vitvi07g04331\_t001 |  |  |  |  |  |  |  |  |  |  |  |  |
| 0 | Vitvi07g04332\_t001 |  |  |  |  |  |  |  |  |  |  |  |  |
| 0 | Vitvi07g04333\_t001 |  |  |  |  |  |  |  |  |  |  |  |  |
| 0 | Vitvi07g04334\_t001 |  |  |  |  |  |  |  |  |  |  |  |  |
| 0 | Vitvi07g04335\_t001 |  |  |  |  |  |  |  |  |  |  |  |  |
| 0 | Vitvi07g04336\_t001 |  |  |  |  |  |  |  |  |  |  |  |  |
| 0 | Vitvi07g04337\_t001 |  |  |  |  |  |  |  |  |  |  |  |  |
| 0 | Vitvi07g01097\_t001 |  |  |  |  |  |  |  |  |  |  |  |  |
| 0 | Vitvi07g04338\_t001 |  |  |  |  |  |  |  |  |  |  |  |  |
| 0 | Vitvi07g04339\_t001 |  |  |  |  |  |  |  |  |  |  |  |  |
| 0 | Vitvi07g04340\_t001 |  |  |  |  |  |  |  |  |  |  |  |  |
| 0 | Vitvi07g04341\_t001 |  |  |  |  |  |  |  |  |  |  |  |  |
| 0 | Vitvi07g04342\_t001 |  |  |  |  |  |  |  |  |  |  |  |  |
| 0 | Vitvi07g01095\_t001 |  |  |  |  |  |  |  |  |  |  |  |  |
| 0 | Vitvi07g04343\_t001 |  |  |  |  |  |  |  |  |  |  |  |  |
| 0 | Vitvi07g04344\_t001 |  |  |  |  |  |  |  |  |  |  |  |  |
| 0 | Vitvi07g02804\_t001 |  |  |  |  |  |  |  |  |  |  |  |  |
| 0 | Vitvi07g04345\_t001 |  |  |  |  |  |  |  |  |  |  |  |  |
| 0 | Vitvi07g01059\_t002 |  |  |  |  |  |  |  |  |  |  |  |  |
| 0 | Vitvi07g01060\_t001 |  |  |  |  |  |  |  |  |  |  |  |  |
| 0 | Vitvi07g01061\_t001 |  |  |  |  |  |  |  |  |  |  |  |  |
| 0 | Vitvi07g01064\_t001 |  |  |  |  |  |  |  |  |  |  |  |  |
| 0 | Vitvi07g01066\_t001 |  |  |  |  |  |  |  |  |  |  |  |  |
| 0 | Vitvi07g04346\_t001 |  |  |  |  |  |  |  |  |  |  |  |  |
| 0 | Vitvi07g04347\_t001 |  |  |  |  |  |  |  |  |  |  |  |  |
| 0 | Vitvi07g04348\_t001 |  |  |  |  |  |  |  |  |  |  |  |  |
| 0 | Vitvi07g04349\_t001 |  |  |  |  |  |  |  |  |  |  |  |  |
| 0 | Vitvi07g04350\_t001 |  |  |  |  |  |  |  |  |  |  |  |  |
| 0 | Vitvi07g04351\_t001 |  |  |  |  |  |  |  |  |  |  |  |  |
| 0 | Vitvi07g04352\_t001 |  |  |  |  |  |  |  |  |  |  |  |  |
| 0 | Vitvi07g02420\_t001 |  |  |  |  |  |  |  |  |  |  |  |  |
| 0 | Vitvi07g02421\_t001 |  |  |  |  |  |  |  |  |  |  |  |  |
| 0 | Vitvi07g02422\_t001 |  |  |  |  |  |  |  |  |  |  |  |  |
| 0 | Vitvi07g04353\_t001 |  |  |  |  |  |  |  |  |  |  |  |  |
| 0 | Vitvi07g04354\_t001 |  |  |  |  |  |  |  |  |  |  |  |  |
| 0 | Vitvi07g04355\_t001 |  |  |  |  |  |  |  |  |  |  |  |  |
| 0 | Vitvi07g04356\_t001 |  |  |  |  |  |  |  |  |  |  |  |  |
| 0 | Vitvi07g04357\_t001 |  |  |  |  |  |  |  |  |  |  |  |  |
| 0 | Vitvi07g04358\_t001 |  |  |  |  |  |  |  |  |  |  |  |  |
| 0 | Vitvi07g04359\_t001 |  |  |  |  |  |  |  |  |  |  |  |  |
| 0 | Vitvi07g04360\_t001 |  |  |  |  |  |  |  |  |  |  |  |  |
| 0 | Vitvi07g01071\_t001 |  |  |  |  |  |  |  |  |  |  |  |  |
| 0 | Vitvi07g02425\_t003 |  |  |  |  |  |  |  |  |  |  |  |  |
| 0 | Vitvi07g01075\_t001 |  |  |  |  |  |  |  |  |  |  |  |  |
| 0 | Vitvi07g04363\_t001 |  |  |  |  |  |  |  |  |  |  |  |  |
| 0 | Vitvi07g02428\_t001 |  |  |  |  |  |  |  |  |  |  |  |  |
| 0 | Vitvi07g04364\_t001 |  |  |  |  |  |  |  |  |  |  |  |  |
| 0 | Vitvi07g02430\_t001 |  |  |  |  |  |  |  |  |  |  |  |  |
| 0 | Vitvi07g04365\_t001 |  |  |  |  |  |  |  |  |  |  |  |  |
| 0 | Vitvi07g04366\_t001 |  |  |  |  |  |  |  |  |  |  |  |  |
| 0 | Vitvi07g04367\_t001 |  |  |  |  |  |  |  |  |  |  |  |  |
| 0 | Vitvi07g04368\_t001 |  |  |  |  |  |  |  |  |  |  |  |  |
| 0 | Vitvi07g04369\_t001 |  |  |  |  |  |  |  |  |  |  |  |  |
| 0 | Vitvi07g04370\_t001 |  |  |  |  |  |  |  |  |  |  |  |  |
| 0 | Vitvi07g04371\_t001 |  |  |  |  |  |  |  |  |  |  |  |  |
| 0 | Vitvi07g04372\_t001 |  |  |  |  |  |  |  |  |  |  |  |  |
| 0 | Vitvi07g04373\_t001 |  |  |  |  |  |  |  |  |  |  |  |  |
| 0 | Vitvi07g02812\_t001 |  |  |  |  |  |  |  |  |  |  |  |  |
| 0 | Vitvi07g04374\_t001 |  |  |  |  |  |  |  |  |  |  |  |  |
| 0 | Vitvi07g02831\_t001 |  |  |  |  |  |  |  |  |  |  |  |  |
| 0 | Vitvi07g04375\_t001 |  |  |  |  |  |  |  |  |  |  |  |  |
| 0 | Vitvi07g02817\_t001 |  |  |  |  |  |  |  |  |  |  |  |  |
| 0 | Vitvi07g02820\_t001 |  |  |  |  |  |  |  |  |  |  |  |  |
| 0 | Vitvi07g04376\_t001 |  |  |  |  |  |  |  |  |  |  |  |  |
| 0 | Vitvi07g04377\_t001 |  |  |  |  |  |  |  |  |  |  |  |  |
| 0 | Vitvi07g02826\_t001 |  |  |  |  |  |  |  |  |  |  |  |  |
| 0 | Vitvi07g04378\_t001 |  |  |  |  |  |  |  |  |  |  |  |  |
| 0 | Vitvi07g04379\_t001 |  |  |  |  |  |  |  |  |  |  |  |  |
| 0 | Vitvi07g04380\_t001 |  |  |  |  |  |  |  |  |  |  |  |  |
| 0 | Vitvi07g04381\_t001 |  |  |  |  |  |  |  |  |  |  |  |  |
| 0 | Vitvi07g02832\_t001 |  |  |  |  |  |  |  |  |  |  |  |  |
| 0 | Vitvi07g04382\_t001 |  |  |  |  |  |  |  |  |  |  |  |  |
| 0 | Vitvi07g04383\_t001 |  |  |  |  |  |  |  |  |  |  |  |  |
| 0 | Vitvi07g04384\_t001 |  |  |  |  |  |  |  |  |  |  |  |  |
| 0 | Vitvi07g04385\_t001 |  |  |  |  |  |  |  |  |  |  |  |  |
| 0 | Vitvi07g04386\_t001 |  |  |  |  |  |  |  |  |  |  |  |  |
| 0 | Vitvi07g04387\_t001 |  |  |  |  |  |  |  |  |  |  |  |  |
| 0 | Vitvi07g04388\_t001 |  |  |  |  |  |  |  |  |  |  |  |  |
| 0 | Vitvi07g04389\_t001 |  |  |  |  |  |  |  |  |  |  |  |  |
| 0 | Vitvi07g04390\_t001 |  |  |  |  |  |  |  |  |  |  |  |  |
| 0 | Vitvi07g04391\_t001 |  |  |  |  |  |  |  |  |  |  |  |  |
| 0 | Vitvi07g04392\_t001 |  |  |  |  |  |  |  |  |  |  |  |  |
| 0 | Vitvi07g01133\_t001 |  |  |  |  |  |  |  |  |  |  |  |  |
| 0 | Vitvi07g04393\_t001 |  |  |  |  |  |  |  |  |  |  |  |  |
| 0 | Vitvi07g04394\_t001 |  |  |  |  |  |  |  |  |  |  |  |  |
| 0 | Vitvi07g01130\_t001 |  |  |  |  |  |  |  |  |  |  |  |  |
| 0 | Vitvi07g01129\_t001 |  |  |  |  |  |  |  |  |  |  |  |  |
| 0 | Vitvi07g04395\_t001 |  |  |  |  |  |  |  |  |  |  |  |  |
| 0 | Vitvi07g01128\_t001 |  |  |  |  |  |  |  |  |  |  |  |  |
| 0 | Vitvi07g04396\_t001 |  |  |  |  |  |  |  |  |  |  |  |  |
| 0 | Vitvi07g04397\_t002 |  |  |  |  |  |  |  |  |  |  |  |  |
| 0 | Vitvi07g01140\_t001 |  |  |  |  |  |  |  |  |  |  |  |  |
| 0 | Vitvi07g04398\_t001 |  |  |  |  |  |  |  |  |  |  |  |  |
| 0 | Vitvi07g01139\_t001 |  |  |  |  |  |  |  |  |  |  |  |  |
| 0 | Vitvi07g04399\_t001 |  |  |  |  |  |  |  |  |  |  |  |  |
| 0 | Vitvi07g01137\_t002 |  |  |  |  |  |  |  |  |  |  |  |  |
| 0 | Vitvi07g04400\_t001 |  |  |  |  |  |  |  |  |  |  |  |  |
| 0 | Vitvi07g04401\_t001 |  |  |  |  |  |  |  |  |  |  |  |  |
| 0 | Vitvi07g04402\_t001 |  |  |  |  |  |  |  |  |  |  |  |  |
| 0 | Vitvi07g04403\_t001 |  |  |  |  |  |  |  |  |  |  |  |  |
| 0 | Vitvi07g01165\_t001 |  |  |  |  |  |  |  |  |  |  |  |  |
| 0 | Vitvi07g01164\_t001 |  |  |  |  |  |  |  |  |  |  |  |  |
| 0 | Vitvi07g01163\_t001 |  |  |  |  |  |  |  |  |  |  |  |  |
| 0 | Vitvi07g01162\_t002 |  |  |  |  |  |  |  |  |  |  |  |  |
| 0 | Vitvi07g01160\_t001 |  |  |  |  |  |  |  |  |  |  |  |  |
| 0 | Vitvi07g01159\_t001 |  |  |  |  |  |  |  |  |  |  |  |  |
| 0 | Vitvi07g04404\_t001 |  |  |  |  |  |  |  |  |  |  |  |  |
| 0 | Vitvi07g04405\_t001 |  |  |  |  |  |  |  |  |  |  |  |  |
| 0 | Vitvi07g04406\_t001 |  |  |  |  |  |  |  |  |  |  |  |  |
| 0 | Vitvi07g04407\_t001 |  |  |  |  |  |  |  |  |  |  |  |  |
| 0 | Vitvi07g01151\_t001 |  |  |  |  |  |  |  |  |  |  |  |  |
| 0 | Vitvi07g04408\_t001 |  |  |  |  |  |  |  |  |  |  |  |  |
| 0 | Vitvi07g04409\_t001 |  |  |  |  |  |  |  |  |  |  |  |  |
| 0 | Vitvi07g02447\_t001 |  |  |  |  |  |  |  |  |  |  |  |  |
| 0 | Vitvi07g04410\_t001 |  |  |  |  |  |  |  |  |  |  |  |  |
| 0 | Vitvi07g01147\_t001 |  |  |  |  |  |  |  |  |  |  |  |  |
| 0 | Vitvi07g02463\_t001 |  |  |  |  |  |  |  |  |  |  |  |  |
| 0 | Vitvi07g04411\_t001 |  |  |  |  |  |  |  |  |  |  |  |  |
| 0 | Vitvi07g02450\_t001 |  |  |  |  |  |  |  |  |  |  |  |  |
| 0 | Vitvi07g02465\_t001 |  |  |  |  |  |  |  |  |  |  |  |  |
| 0 | Vitvi07g04412\_t001 |  |  |  |  |  |  |  |  |  |  |  |  |
| 0 | Vitvi07g04413\_t001 |  |  |  |  |  |  |  |  |  |  |  |  |
| 0 | Vitvi07g04414\_t001 |  |  |  |  |  |  |  |  |  |  |  |  |
| 0 | Vitvi07g03153\_t001 |  |  |  |  |  |  |  |  |  |  |  |  |
| 0 | Vitvi07g01178\_t001 |  |  |  |  |  |  |  |  |  |  |  |  |
| 0 | Vitvi07g01179\_t001 |  |  |  |  |  |  |  |  |  |  |  |  |
| 0 | Vitvi07g02467\_t002 |  |  |  |  |  |  |  |  |  |  |  |  |
| 0 | Vitvi07g01180\_t001 |  |  |  |  |  |  |  |  |  |  |  |  |
| 0 | Vitvi07g01181\_t001 |  |  |  |  |  |  |  |  |  |  |  |  |
| 0 | Vitvi07g01182\_t001 |  |  |  |  |  |  |  |  |  |  |  |  |
| 0 | Vitvi07g01183\_t001 |  |  |  |  |  |  |  |  |  |  |  |  |
| 0 | Vitvi07g04415\_t001 |  |  |  |  |  |  |  |  |  |  |  |  |
| 0 | Vitvi07g04416\_t001 |  |  |  |  |  |  |  |  |  |  |  |  |
| 0 | Vitvi07g04417\_t001 |  |  |  |  |  |  |  |  |  |  |  |  |
| 0 | Vitvi07g04418\_t001 |  |  |  |  |  |  |  |  |  |  |  |  |
| 0 | Vitvi07g04419\_t001 |  |  |  |  |  |  |  |  |  |  |  |  |
| 0 | Vitvi07g04420\_t001 |  |  |  |  |  |  |  |  |  |  |  |  |
| 0 | Vitvi07g04421\_t001 |  |  |  |  |  |  |  |  |  |  |  |  |
| 0 | Vitvi07g04422\_t001 |  |  |  |  |  |  |  |  |  |  |  |  |
| 0 | Vitvi07g04423\_t001 |  |  |  |  |  |  |  |  |  |  |  |  |
| 0 | Vitvi07g04424\_t001 |  |  |  |  |  |  |  |  |  |  |  |  |
| 0 | Vitvi07g04425\_t001 |  |  |  |  |  |  |  |  |  |  |  |  |
| 0 | Vitvi07g04426\_t001 |  |  |  |  |  |  |  |  |  |  |  |  |
| 0 | Vitvi07g04427\_t001 |  |  |  |  |  |  |  |  |  |  |  |  |
| 0 | Vitvi07g04428\_t001 |  |  |  |  |  |  |  |  |  |  |  |  |
| 0 | Vitvi07g04429\_t001 |  |  |  |  |  |  |  |  |  |  |  |  |
| 0 | Vitvi07g04430\_t001 |  |  |  |  |  |  |  |  |  |  |  |  |
| 0 | Vitvi07g02962\_t001 |  |  |  |  |  |  |  |  |  |  |  |  |
| 0 | Vitvi07g02963\_t001 |  |  |  |  |  |  |  |  |  |  |  |  |
| 0 | Vitvi07g04431\_t001 |  |  |  |  |  |  |  |  |  |  |  |  |
| 0 | Vitvi07g02969\_t001 |  |  |  |  |  |  |  |  |  |  |  |  |
| 0 | Vitvi07g02970\_t001 |  |  |  |  |  |  |  |  |  |  |  |  |
| 0 | Vitvi07g02971\_t001 |  |  |  |  |  |  |  |  |  |  |  |  |
| 0 | Vitvi07g04432\_t001 |  |  |  |  |  |  |  |  |  |  |  |  |
| 0 | Vitvi07g04433\_t001 |  |  |  |  |  |  |  |  |  |  |  |  |
| 0 | Vitvi07g04434\_t001 |  |  |  |  |  |  |  |  |  |  |  |  |
| 0 | Vitvi07g04435\_t001 |  |  |  |  |  |  |  |  |  |  |  |  |
| 0 | Vitvi07g04436\_t001 |  |  |  |  |  |  |  |  |  |  |  |  |
| 0 | Vitvi07g04437\_t001 |  |  |  |  |  |  |  |  |  |  |  |  |
| 0 | Vitvi07g04438\_t001 |  |  |  |  |  |  |  |  |  |  |  |  |
| 0 | Vitvi07g04439\_t001 |  |  |  |  |  |  |  |  |  |  |  |  |
| 0 | Vitvi07g04440\_t001 |  |  |  |  |  |  |  |  |  |  |  |  |
| 0 | Vitvi07g03001\_t001 |  |  |  |  |  |  |  |  |  |  |  |  |
| 0 | Vitvi07g04441\_t001 |  |  |  |  |  |  |  |  |  |  |  |  |
| 0 | Vitvi07g02461\_t001 |  |  |  |  |  |  |  |  |  |  |  |  |
| 0 | Vitvi07g04443\_t001 |  |  |  |  |  |  |  |  |  |  |  |  |
| 0 | Vitvi07g04444\_t001 |  |  |  |  |  |  |  |  |  |  |  |  |
| 0 | Vitvi07g04445\_t001 |  |  |  |  |  |  |  |  |  |  |  |  |
| 0 | Vitvi07g04446\_t002 |  |  |  |  |  |  |  |  |  |  |  |  |
| 0 | Vitvi07g04447\_t001 |  |  |  |  |  |  |  |  |  |  |  |  |
| 0 | Vitvi07g01198\_t001 |  |  |  |  |  |  |  |  |  |  |  |  |
| 0 | Vitvi07g01199\_t001 |  |  |  |  |  |  |  |  |  |  |  |  |
| 0 | Vitvi07g02480\_t002 |  |  |  |  |  |  |  |  |  |  |  |  |
| 0 | Vitvi07g01200\_t001 |  |  |  |  |  |  |  |  |  |  |  |  |
| 0 | Vitvi07g02481\_t001 |  |  |  |  |  |  |  |  |  |  |  |  |
| 0 | Vitvi07g04448\_t001 |  |  |  |  |  |  |  |  |  |  |  |  |
| 0 | Vitvi07g01202\_t001 |  |  |  |  |  |  |  |  |  |  |  |  |
| 0 | Vitvi07g02482\_t002 |  |  |  |  |  |  |  |  |  |  |  |  |
| 0 | Vitvi07g04449\_t001 |  |  |  |  |  |  |  |  |  |  |  |  |
| 0 | Vitvi07g04450\_t001 |  |  |  |  |  |  |  |  |  |  |  |  |
| 0 | Vitvi07g04451\_t001 |  |  |  |  |  |  |  |  |  |  |  |  |
| 0 | Vitvi07g01203\_t005 |  |  |  |  |  |  |  |  |  |  |  |  |
| 0 | Vitvi07g04452\_t001 |  |  |  |  |  |  |  |  |  |  |  |  |
| 0 | Vitvi07g02484\_t001 |  |  |  |  |  |  |  |  |  |  |  |  |
| 0 | Vitvi07g02485\_t001 |  |  |  |  |  |  |  |  |  |  |  |  |
| 0 | Vitvi07g04453\_t001 |  |  |  |  |  |  |  |  |  |  |  |  |
| 0 | Vitvi07g04454\_t001 |  |  |  |  |  |  |  |  |  |  |  |  |
| 0 | Vitvi07g04455\_t001 |  |  |  |  |  |  |  |  |  |  |  |  |
| 0 | Vitvi07g01205\_t001 |  |  |  |  |  |  |  |  |  |  |  |  |
| 0 | Vitvi07g01209\_t001 |  |  |  |  |  |  |  |  |  |  |  |  |
| 0 | Vitvi07g02487\_t001 |  |  |  |  |  |  |  |  |  |  |  |  |
| 0 | Vitvi07g01211\_t001 |  |  |  |  |  |  |  |  |  |  |  |  |
| 0 | Vitvi07g04456\_t001 |  |  |  |  |  |  |  |  |  |  |  |  |
| 0 | Vitvi07g02488\_t001 |  |  |  |  |  |  |  |  |  |  |  |  |
| 0 | Vitvi07g04457\_t001 |  |  |  |  |  |  |  |  |  |  |  |  |
| 0 | Vitvi07g04458\_t001 |  |  |  |  |  |  |  |  |  |  |  |  |
| 0 | Vitvi07g04459\_t001 |  |  |  |  |  |  |  |  |  |  |  |  |
| 0 | Vitvi07g04460\_t001 |  |  |  |  |  |  |  |  |  |  |  |  |
| 0 | Vitvi07g04461\_t001 |  |  |  |  |  |  |  |  |  |  |  |  |
| 0 | Vitvi07g04462\_t001 |  |  |  |  |  |  |  |  |  |  |  |  |
| 0 | Vitvi07g02491\_t002 |  |  |  |  |  |  |  |  |  |  |  |  |
| 0 | Vitvi07g04463\_t002 |  |  |  |  |  |  |  |  |  |  |  |  |
| 0 | Vitvi07g01215\_t001 |  |  |  |  |  |  |  |  |  |  |  |  |
| 0 | Vitvi07g04464\_t001 |  |  |  |  |  |  |  |  |  |  |  |  |
| 0 | Vitvi07g01217\_t001 |  |  |  |  |  |  |  |  |  |  |  |  |
| 0 | Vitvi07g01219\_t001 |  |  |  |  |  |  |  |  |  |  |  |  |
| 0 | Vitvi07g04465\_t001 |  |  |  |  |  |  |  |  |  |  |  |  |
| 0 | Vitvi07g01221\_t001 |  |  |  |  |  |  |  |  |  |  |  |  |
| 0 | Vitvi07g04466\_t001 |  |  |  |  |  |  |  |  |  |  |  |  |
| 0 | Vitvi07g04467\_t001 |  |  |  |  |  |  |  |  |  |  |  |  |
| 0 | Vitvi07g04468\_t001 |  |  |  |  |  |  |  |  |  |  |  |  |
| 0 | Vitvi07g04469\_t001 |  |  |  |  |  |  |  |  |  |  |  |  |
| 0 | Vitvi07g04470\_t001 |  |  |  |  |  |  |  |  |  |  |  |  |
| 0 | Vitvi07g01225\_t001 |  |  |  |  |  |  |  |  |  |  |  |  |
| 0 | Vitvi07g04471\_t001 |  |  |  |  |  |  |  |  |  |  |  |  |
| 0 | Vitvi07g03118\_t001 |  |  |  |  |  |  |  |  |  |  |  |  |
| 0 | Vitvi07g02995\_t001 |  |  |  |  |  |  |  |  |  |  |  |  |
| 0 | Vitvi07g04472\_t001 |  |  |  |  |  |  |  |  |  |  |  |  |
| 0 | Vitvi07g04473\_t001 |  |  |  |  |  |  |  |  |  |  |  |  |
| 0 | Vitvi07g04474\_t001 |  |  |  |  |  |  |  |  |  |  |  |  |
| 0 | Vitvi07g04475\_t001 |  |  |  |  |  |  |  |  |  |  |  |  |
| 0 | Vitvi07g04476\_t001 |  |  |  |  |  |  |  |  |  |  |  |  |
| 0 | Vitvi07g04477\_t001 |  |  |  |  |  |  |  |  |  |  |  |  |
| 0 | Vitvi07g04478\_t001 |  |  |  |  |  |  |  |  |  |  |  |  |
| 0 | Vitvi07g01231\_t002 |  |  |  |  |  |  |  |  |  |  |  |  |
| 0 | Vitvi07g01233\_t001 |  |  |  |  |  |  |  |  |  |  |  |  |
| 0 | Vitvi07g01234\_t001 |  |  |  |  |  |  |  |  |  |  |  |  |
| 0 | Vitvi07g04479\_t001 |  |  |  |  |  |  |  |  |  |  |  |  |
| 0 | Vitvi07g01236\_t001 |  |  |  |  |  |  |  |  |  |  |  |  |
| 0 | Vitvi07g01237\_t001 |  |  |  |  |  |  |  |  |  |  |  |  |
| 0 | Vitvi07g02992\_t001 |  |  |  |  |  |  |  |  |  |  |  |  |
| 0 | Vitvi07g02991\_t001 |  |  |  |  |  |  |  |  |  |  |  |  |
| 0 | Vitvi07g02990\_t001 |  |  |  |  |  |  |  |  |  |  |  |  |
| 0 | Vitvi07g04480\_t001 |  |  |  |  |  |  |  |  |  |  |  |  |
| 0 | Vitvi07g04481\_t001 |  |  |  |  |  |  |  |  |  |  |  |  |
| 0 | Vitvi07g04482\_t001 |  |  |  |  |  |  |  |  |  |  |  |  |
| 0 | Vitvi07g04483\_t001 |  |  |  |  |  |  |  |  |  |  |  |  |
| 2 | Vitvi07g01242\_t001 |  | Vitvi05g01683\_t001 |  | Vitvi18g00901\_t001 |  |  |  |  |  |  |  |  |
| 2 | Vitvi07g02498\_t001 |  | Vitvi05g01679\_t001 |  | \| \| |  |  |  |  |  |  |  |  |
| 2 | Vitvi07g01243\_t001 |  | \| \| |  | \| \| |  |  |  |  |  |  |  |  |
| 2 | Vitvi07g01244\_t001 |  | \| \| |  | \| \| |  |  |  |  |  |  |  |  |
| 2 | Vitvi07g04484\_t001 |  | \| \| |  | \| \| |  |  |  |  |  |  |  |  |
| 2 | Vitvi07g01246\_t001 |  | Vitvi05g01676\_t001 |  | \| \| |  |  |  |  |  |  |  |  |
| 2 | Vitvi07g01247\_t001 |  | Vitvi05g01660\_t001 |  | \| \| |  |  |  |  |  |  |  |  |
| 2 | Vitvi07g04485\_t002 |  | Vitvi05g01655\_t001 |  | \| \| |  |  |  |  |  |  |  |  |
| 2 | Vitvi07g01249\_t001 |  | \| \| |  | \| \| |  |  |  |  |  |  |  |  |
| 2 | Vitvi07g01250\_t001 |  | \| \| |  | \| \| |  |  |  |  |  |  |  |  |
| 2 | Vitvi07g01251\_t001 |  | \| \| |  | \| \| |  |  |  |  |  |  |  |  |
| 2 | Vitvi07g01252\_t001 |  | Vitvi05g01640\_t001 |  | \| \| |  |  |  |  |  |  |  |  |
| 2 | Vitvi07g01253\_t001 |  | \| \| |  | \| \| |  |  |  |  |  |  |  |  |
| 2 | Vitvi07g02984\_t001 |  | \| \| |  | \| \| |  |  |  |  |  |  |  |  |
| 2 | Vitvi07g02983\_t001 |  | \| \| |  | \| \| |  |  |  |  |  |  |  |  |
| 2 | Vitvi07g02982\_t001 |  | Vitvi05g01619\_t001 |  | \| \| |  |  |  |  |  |  |  |  |
| 1 | Vitvi07g04486\_t001 |  |  |  | \| \| |  |  |  |  |  |  |  |  |
| 1 | Vitvi07g04487\_t001 |  |  |  | \| \| |  |  |  |  |  |  |  |  |
| 1 | Vitvi07g01255\_t001 |  |  |  | \| \| |  |  |  |  |  |  |  |  |
| 1 | Vitvi07g02500\_t002 |  |  |  | \| \| |  |  |  |  |  |  |  |  |
| 1 | Vitvi07g04488\_t001 |  |  |  | \| \| |  |  |  |  |  |  |  |  |
| 1 | Vitvi07g04489\_t001 |  |  |  | \| \| |  |  |  |  |  |  |  |  |
| 2 | Vitvi07g01261\_t001 |  | Vitvi03g00377\_t001 |  | Vitvi18g00897\_t001 |  |  |  |  |  |  |  |  |
| 2 | Vitvi07g01262\_t001 |  | \| \| |  | \| \| |  |  |  |  |  |  |  |  |
| 2 | Vitvi07g04490\_t001 |  | \| \| |  | \| \| |  |  |  |  |  |  |  |  |
| 2 | Vitvi07g04491\_t001 |  | \| \| |  | \| \| |  |  |  |  |  |  |  |  |
| 2 | Vitvi07g01264\_t001 |  | Vitvi03g00378\_t001 |  | \| \| |  |  |  |  |  |  |  |  |
| 2 | Vitvi07g01265\_t001 |  | \| \| |  | \| \| |  |  |  |  |  |  |  |  |
| 2 | Vitvi07g01266\_t001 |  | \| \| |  | \| \| |  |  |  |  |  |  |  |  |
| 2 | Vitvi07g04492\_t001 |  | \| \| |  | \| \| |  |  |  |  |  |  |  |  |
| 2 | Vitvi07g02502\_t001 |  | Vitvi03g00380\_t001 |  | \| \| |  |  |  |  |  |  |  |  |
| 2 | Vitvi07g01267\_t001 |  | Vitvi03g01518\_t001 |  | \| \| |  |  |  |  |  |  |  |  |
| 2 | Vitvi07g01273\_t001 |  | \| \| |  | \| \| |  |  |  |  |  |  |  |  |
| 2 | Vitvi07g01274\_t001 |  | Vitvi03g00384\_t001 |  | \| \| |  |  |  |  |  |  |  |  |
| 2 | Vitvi07g04493\_t001 |  | \| \| |  | \| \| |  |  |  |  |  |  |  |  |
| 2 | Vitvi07g01275\_t001 |  | \| \| |  | Vitvi18g00894\_t001 |  |  |  |  |  |  |  |  |
| 2 | Vitvi07g01276\_t001 |  | Vitvi03g00389\_t001 |  | \| \| |  |  |  |  |  |  |  |  |
| 2 | Vitvi07g04494\_t001 |  | \| \| |  | \| \| |  |  |  |  |  |  |  |  |
| 2 | Vitvi07g01277\_t001 |  | \| \| |  | \| \| |  |  |  |  |  |  |  |  |
| 2 | Vitvi07g01278\_t002 |  | \| \| |  | Vitvi18g04213\_t001 |  |  |  |  |  |  |  |  |
| 2 | Vitvi07g01279\_t001 |  | \| \| |  | \| \| |  |  |  |  |  |  |  |  |
| 2 | Vitvi07g02504\_t001 |  | \| \| |  | \| \| |  |  |  |  |  |  |  |  |
| 2 | Vitvi07g01280\_t001 |  | \| \| |  | Vitvi18g00885\_t001 |  |  |  |  |  |  |  |  |
| 2 | Vitvi07g01281\_t001 |  | \| \| |  | \| \| |  |  |  |  |  |  |  |  |
| 2 | Vitvi07g04495\_t001 |  | \| \| |  | Vitvi18g02724\_t001 |  |  |  |  |  |  |  |  |
| 2 | Vitvi07g04496\_t001 |  | \| \| |  | \| \| |  |  |  |  |  |  |  |  |
| 2 | Vitvi07g04497\_t001 |  | \| \| |  | \| \| |  |  |  |  |  |  |  |  |
| 2 | Vitvi07g04498\_t001 |  | \| \| |  | \| \| |  |  |  |  |  |  |  |  |
| 2 | Vitvi07g01286\_t001 |  | Vitvi03g00397\_t001 |  | Vitvi18g00882\_t001 |  |  |  |  |  |  |  |  |
| 2 | Vitvi07g04499\_t001 |  | \| \| |  | \| \| |  |  |  |  |  |  |  |  |
| 2 | Vitvi07g01287\_t001 |  | Vitvi03g00398\_t001 |  | \| \| |  |  |  |  |  |  |  |  |
| 1 | Vitvi07g04500\_t001 |  |  |  | \| \| |  |  |  |  |  |  |  |  |
| 1 | Vitvi07g04501\_t001 |  |  |  | \| \| |  |  |  |  |  |  |  |  |
| 1 | Vitvi07g01288\_t001 |  |  |  | Vitvi18g02718\_t001 |  |  |  |  |  |  |  |  |
| 2 | Vitvi07g04502\_t001 |  | Vitvi03g04147\_t001 |  | \| \| |  |  |  |  |  |  |  |  |
| 2 | Vitvi07g04503\_t001 |  | Vitvi03g04151\_t001 |  | \| \| |  |  |  |  |  |  |  |  |
| 2 | Vitvi07g04504\_t001 |  | \| \| |  | \| \| |  |  |  |  |  |  |  |  |
| 2 | Vitvi07g01291\_t001 |  | Vitvi03g00416\_t001 |  | Vitvi18g00866\_t001 |  |  |  |  |  |  |  |  |
| 2 | Vitvi07g01292\_t001 |  | \| \| |  | \| \| |  |  |  |  |  |  |  |  |
| 2 | Vitvi07g01293\_t001 |  | Vitvi03g00419\_t001 |  | \| \| |  |  |  |  |  |  |  |  |
| 2 | Vitvi07g01294\_t001 |  | \| \| |  | \| \| |  |  |  |  |  |  |  |  |
| 2 | Vitvi07g01295\_t001 |  | \| \| |  | Vitvi18g00863\_t001 |  |  |  |  |  |  |  |  |
| 2 | Vitvi07g01296\_t001 |  | Vitvi03g00423\_t001 |  | \| \| |  |  |  |  |  |  |  |  |
| 2 | Vitvi07g01297\_t001 |  | \| \| |  | \| \| |  |  |  |  |  |  |  |  |
| 2 | Vitvi07g01298\_t001 |  | Vitvi03g00430\_t001 |  | Vitvi18g00858\_t001 |  |  |  |  |  |  |  |  |
| 2 | Vitvi07g04505\_t001 |  | \| \| |  | \| \| |  |  |  |  |  |  |  |  |
| 2 | Vitvi07g04506\_t001 |  | \| \| |  | \| \| |  |  |  |  |  |  |  |  |
| 2 | Vitvi07g04507\_t001 |  | \| \| |  | \| \| |  |  |  |  |  |  |  |  |
| 2 | Vitvi07g04508\_t001 |  | \| \| |  | \| \| |  |  |  |  |  |  |  |  |
| 2 | Vitvi07g02512\_t001 |  | Vitvi03g00433\_t001 |  | \| \| |  |  |  |  |  |  |  |  |
| 2 | Vitvi07g02513\_t001 |  | Vitvi03g04160\_t001 |  | \| \| |  |  |  |  |  |  |  |  |
| 1 | Vitvi07g01300\_t001 |  |  |  | \| \| |  |  |  |  |  |  |  |  |
| 1 | Vitvi07g02516\_t001 |  |  |  | \| \| |  |  |  |  |  |  |  |  |
| 1 | Vitvi07g02517\_t001 |  |  |  | \| \| |  |  |  |  |  |  |  |  |
| 1 | Vitvi07g04509\_t001 |  |  |  | \| \| |  |  |  |  |  |  |  |  |
| 1 | Vitvi07g01302\_t003 |  |  |  | \| \| |  |  |  |  |  |  |  |  |
| 1 | Vitvi07g04510\_t001 |  |  |  | \| \| |  |  |  |  |  |  |  |  |
| 1 | Vitvi07g01306\_t001 |  |  |  | \| \| |  |  |  |  |  |  |  |  |
| 1 | Vitvi07g04511\_t001 |  |  |  | \| \| |  |  |  |  |  |  |  |  |
| 1 | Vitvi07g04512\_t001 |  |  |  | \| \| |  |  |  |  |  |  |  |  |
| 1 | Vitvi07g01308\_t002 |  |  |  | \| \| |  |  |  |  |  |  |  |  |
| 1 | Vitvi07g01309\_t001 |  |  |  | \| \| |  |  |  |  |  |  |  |  |
| 1 | Vitvi07g04513\_t001 |  |  |  | \| \| |  |  |  |  |  |  |  |  |
| 1 | Vitvi07g04514\_t001 |  |  |  | \| \| |  |  |  |  |  |  |  |  |
| 1 | Vitvi07g04515\_t001 |  |  |  | \| \| |  |  |  |  |  |  |  |  |
| 1 | Vitvi07g02519\_t001 |  |  |  | \| \| |  |  |  |  |  |  |  |  |
| 1 | Vitvi07g01312\_t001 |  |  |  | \| \| |  |  |  |  |  |  |  |  |
| 1 | Vitvi07g01313\_t001 |  |  |  | Vitvi18g00850\_t001 |  |  |  |  |  |  |  |  |
| 1 | Vitvi07g02521\_t001 |  |  |  | \| \| |  |  |  |  |  |  |  |  |
| 1 | Vitvi07g04516\_t001 |  |  |  | \| \| |  |  |  |  |  |  |  |  |
| 1 | Vitvi07g04517\_t001 |  |  |  | \| \| |  |  |  |  |  |  |  |  |
| 1 | Vitvi07g04518\_t001 |  |  |  | \| \| |  |  |  |  |  |  |  |  |
| 1 | Vitvi07g01315\_t001 |  |  |  | \| \| |  |  |  |  |  |  |  |  |
| 1 | Vitvi07g04519\_t001 |  |  |  | \| \| |  |  |  |  |  |  |  |  |
| 1 | Vitvi07g04520\_t001 |  |  |  | \| \| |  |  |  |  |  |  |  |  |
| 1 | Vitvi07g01317\_t001 |  |  |  | \| \| |  |  |  |  |  |  |  |  |
| 1 | Vitvi07g04521\_t001 |  |  |  | \| \| |  |  |  |  |  |  |  |  |
| 1 | Vitvi07g04522\_t001 |  |  |  | \| \| |  |  |  |  |  |  |  |  |
| 1 | Vitvi07g04523\_t001 |  |  |  | \| \| |  |  |  |  |  |  |  |  |
| 1 | Vitvi07g04524\_t001 |  |  |  | \| \| |  |  |  |  |  |  |  |  |
| 1 | Vitvi07g04525\_t001 |  |  |  | \| \| |  |  |  |  |  |  |  |  |
| 1 | Vitvi07g01323\_t001 |  |  |  | \| \| |  |  |  |  |  |  |  |  |
| 1 | Vitvi07g04526\_t001 |  |  |  | \| \| |  |  |  |  |  |  |  |  |
| 1 | Vitvi07g01324\_t001 |  |  |  | \| \| |  |  |  |  |  |  |  |  |
| 1 | Vitvi07g01325\_t001 |  |  |  | \| \| |  |  |  |  |  |  |  |  |
| 1 | Vitvi07g04527\_t001 |  |  |  | \| \| |  |  |  |  |  |  |  |  |
| 1 | Vitvi07g02529\_t001.1.6037826f |  |  |  | \| \| |  |  |  |  |  |  |  |  |
| 1 | Vitvi07g04528\_t001 |  |  |  | \| \| |  |  |  |  |  |  |  |  |
| 1 | Vitvi07g01326\_t001 |  |  |  | \| \| |  |  |  |  |  |  |  |  |
| 1 | Vitvi07g04529\_t001 |  |  |  | \| \| |  |  |  |  |  |  |  |  |
| 1 | Vitvi07g01327\_t001 |  |  |  | \| \| |  |  |  |  |  |  |  |  |
| 1 | Vitvi07g01328\_t001 |  |  |  | \| \| |  |  |  |  |  |  |  |  |
| 1 | Vitvi07g01329\_t001 |  |  |  | \| \| |  |  |  |  |  |  |  |  |
| 1 | Vitvi07g04530\_t001 |  |  |  | Vitvi18g00849\_t001 |  |  |  |  |  |  |  |  |
| 1 | Vitvi07g02075\_t001 |  |  |  | \| \| |  |  |  |  |  |  |  |  |
| 1 | Vitvi07g02541\_t001 |  |  |  | \| \| |  |  |  |  |  |  |  |  |
| 1 | Vitvi07g01368\_t003 |  |  |  | \| \| |  |  |  |  |  |  |  |  |
| 1 | Vitvi07g04531\_t001 |  |  |  | \| \| |  |  |  |  |  |  |  |  |
| 1 | Vitvi07g01366\_t001 |  |  |  | \| \| |  |  |  |  |  |  |  |  |
| 1 | Vitvi07g04532\_t001 |  |  |  | \| \| |  |  |  |  |  |  |  |  |
| 1 | Vitvi07g04533\_t001 |  |  |  | \| \| |  |  |  |  |  |  |  |  |
| 1 | Vitvi07g01345\_t001 |  |  |  | \| \| |  |  |  |  |  |  |  |  |
| 1 | Vitvi07g01342\_t001 |  |  |  | \| \| |  |  |  |  |  |  |  |  |
| 1 | Vitvi07g04534\_t001 |  |  |  | \| \| |  |  |  |  |  |  |  |  |
| 1 | Vitvi07g04535\_t001 |  |  |  | \| \| |  |  |  |  |  |  |  |  |
| 1 | Vitvi07g01337\_t001 |  |  |  | \| \| |  |  |  |  |  |  |  |  |
| 1 | Vitvi07g01335\_t001 |  |  |  | \| \| |  |  |  |  |  |  |  |  |
| 1 | Vitvi07g04536\_t001 |  |  |  | \| \| |  |  |  |  |  |  |  |  |
| 1 | Vitvi07g01333\_t001 |  |  |  | \| \| |  |  |  |  |  |  |  |  |
| 1 | Vitvi07g01332\_t001 |  |  |  | \| \| |  |  |  |  |  |  |  |  |
| 1 | Vitvi07g02531\_t001 |  |  |  | \| \| |  |  |  |  |  |  |  |  |
| 1 | Vitvi07g04537\_t001 |  |  |  | \| \| |  |  |  |  |  |  |  |  |
| 1 | Vitvi07g01331\_t001 |  |  |  | \| \| |  |  |  |  |  |  |  |  |
| 1 | Vitvi07g01330\_t001 |  |  |  | \| \| |  |  |  |  |  |  |  |  |
| 1 | Vitvi07g04538\_t001 |  |  |  | \| \| |  |  |  |  |  |  |  |  |
| 1 | Vitvi10g02293\_t001 |  |  |  | \| \| |  |  |  |  |  |  |  |  |
| 1 | Vitvi07g04539\_t001 |  |  |  | \| \| |  |  |  |  |  |  |  |  |
| 1 | Vitvi07g04540\_t001 |  |  |  | \| \| |  |  |  |  |  |  |  |  |
| 1 | Vitvi07g04541\_t001 |  |  |  | Vitvi18g00838\_t001 |  |  |  |  |  |  |  |  |
| 0 | Vitvi07g04542\_t001 |  |  |  |  |  |  |  |  |  |  |  |  |
| 1 | Vitvi07g04543\_t001 |  | Vitvi18g02569\_t001 |  |  |  |  |  |  |  |  |  |  |
| 1 | Vitvi07g02535\_t001 |  | \| \| |  |  |  |  |  |  |  |  |  |  |
| 1 | Vitvi07g02536\_t001 |  | Vitvi18g02570\_t001 |  |  |  |  |  |  |  |  |  |  |
| 1 | Vitvi07g02537\_t001 |  | \| \| |  |  |  |  |  |  |  |  |  |  |
| 1 | Vitvi07g02538\_t001 |  | \| \| |  |  |  |  |  |  |  |  |  |  |
| 1 | Vitvi07g01350\_t001 |  | \| \| |  |  |  |  |  |  |  |  |  |  |
| 1 | Vitvi07g01351\_t001 |  | Vitvi18g00371\_t001 |  |  |  |  |  |  |  |  |  |  |
| 1 | Vitvi07g01352\_t001 |  | \| \| |  |  |  |  |  |  |  |  |  |  |
| 1 | Vitvi07g01353\_t001 |  | \| \| |  |  |  |  |  |  |  |  |  |  |
| 1 | Vitvi07g01354\_t001 |  | \| \| |  |  |  |  |  |  |  |  |  |  |
| 1 | Vitvi07g04544\_t001 |  | \| \| |  |  |  |  |  |  |  |  |  |  |
| 1 | Vitvi07g03131\_t001 |  | \| \| |  |  |  |  |  |  |  |  |  |  |
| 1 | Vitvi07g03026\_t001 |  | \| \| |  |  |  |  |  |  |  |  |  |  |
| 1 | Vitvi07g04545\_t001 |  | \| \| |  |  |  |  |  |  |  |  |  |  |
| 1 | Vitvi07g03031\_t001 |  | \| \| |  |  |  |  |  |  |  |  |  |  |
| 1 | Vitvi07g04546\_t001 |  | \| \| |  |  |  |  |  |  |  |  |  |  |
| 1 | Vitvi07g03030\_t001 |  | Vitvi18g00381\_t001 |  |  |  |  |  |  |  |  |  |  |
| 1 | Vitvi07g01365\_t001 |  | Vitvi18g00384\_t001 |  |  |  |  |  |  |  |  |  |  |
| 1 | Vitvi07g04547\_t001 |  | \| \| |  |  |  |  |  |  |  |  |  |  |
| 1 | Vitvi07g02540\_t001 |  | Vitvi18g00385\_t001 |  |  |  |  |  |  |  |  |  |  |
| 1 | Vitvi07g01364\_t001 |  | \| \| |  |  |  |  |  |  |  |  |  |  |
| 1 | Vitvi07g01363\_t001 |  | Vitvi18g00390\_t001 |  |  |  |  |  |  |  |  |  |  |
| 1 | Vitvi07g01361\_t001 |  | \| \| |  |  |  |  |  |  |  |  |  |  |
| 1 | Vitvi07g01360\_t001 |  | \| \| |  |  |  |  |  |  |  |  |  |  |
| 1 | Vitvi07g01359\_t001 |  | Vitvi18g00391\_t001 |  |  |  |  |  |  |  |  |  |  |
| 1 | Vitvi07g01358\_t001 |  | Vitvi18g00392\_t001 |  |  |  |  |  |  |  |  |  |  |
| 1 | Vitvi07g04548\_t001 |  | \| \| |  |  |  |  |  |  |  |  |  |  |
| 1 | Vitvi07g04549\_t001 |  | \| \| |  |  |  |  |  |  |  |  |  |  |
| 1 | Vitvi07g04550\_t001 |  | \| \| |  |  |  |  |  |  |  |  |  |  |
| 2 | Vitvi07g04551\_t001 |  | \| \| |  | Vitvi00g04283\_t001 |  |  |  |  |  |  |  |  |
| 2 | Vitvi07g04552\_t001 |  | \| \| |  | Vitvi00g04282\_t001 |  |  |  |  |  |  |  |  |
| 2 | Vitvi07g04553\_t001 |  | \| \| |  | Vitvi00g04280\_t001 |  |  |  |  |  |  |  |  |
| 2 | Vitvi07g04554\_t001 |  | \| \| |  | Vitvi00g04279\_t001 |  |  |  |  |  |  |  |  |
| 2 | Vitvi07g01380\_t001 |  | \| \| |  | Vitvi00g04275\_t001 |  |  |  |  |  |  |  |  |
| 2 | Vitvi07g02548\_t001 |  | \| \| |  | Vitvi00g04274\_t002 |  |  |  |  |  |  |  |  |
| 2 | Vitvi07g01381\_t001 |  | \| \| |  | Vitvi00g04273\_t001 |  |  |  |  |  |  |  |  |
| 1 | Vitvi07g04555\_t001 |  | \| \| |  |  |  |  |  |  |  |  |  |  |
| 1 | Vitvi07g04556\_t002 |  | \| \| |  |  |  |  |  |  |  |  |  |  |
| 1 | Vitvi07g04557\_t001 |  | Vitvi18g00394\_t001 |  |  |  |  |  |  |  |  |  |  |
| 1 | Vitvi07g04558\_t001 |  | \| \| |  |  |  |  |  |  |  |  |  |  |
| 1 | Vitvi07g04559\_t001 |  | \| \| |  |  |  |  |  |  |  |  |  |  |
| 1 | Vitvi07g04560\_t001 |  | \| \| |  |  |  |  |  |  |  |  |  |  |
| 1 | Vitvi07g04561\_t001 |  | \| \| |  |  |  |  |  |  |  |  |  |  |
| 1 | Vitvi07g04562\_t001 |  | \| \| |  |  |  |  |  |  |  |  |  |  |
| 1 | Vitvi07g04563\_t001 |  | \| \| |  |  |  |  |  |  |  |  |  |  |
| 1 | Vitvi07g03178\_t001 |  | \| \| |  |  |  |  |  |  |  |  |  |  |
| 1 | Vitvi07g04564\_t001 |  | \| \| |  |  |  |  |  |  |  |  |  |  |
| 1 | Vitvi07g04565\_t001 |  | \| \| |  |  |  |  |  |  |  |  |  |  |
| 1 | Vitvi07g04566\_t001 |  | \| \| |  |  |  |  |  |  |  |  |  |  |
| 1 | Vitvi07g04567\_t001 |  | \| \| |  |  |  |  |  |  |  |  |  |  |
| 1 | Vitvi07g04568\_t001 |  | \| \| |  |  |  |  |  |  |  |  |  |  |
| 1 | Vitvi07g04569\_t001 |  | Vitvi18g02574\_t002 |  |  |  |  |  |  |  |  |  |  |
| 1 | Vitvi07g04570\_t001 |  | \| \| |  |  |  |  |  |  |  |  |  |  |
| 1 | Vitvi07g04571\_t001 |  | \| \| |  |  |  |  |  |  |  |  |  |  |
| 1 | Vitvi07g04572\_t001 |  | \| \| |  |  |  |  |  |  |  |  |  |  |
| 1 | Vitvi07g04573\_t001 |  | \| \| |  |  |  |  |  |  |  |  |  |  |
| 1 | Vitvi07g01392\_t001 |  | \| \| |  |  |  |  |  |  |  |  |  |  |
| 1 | Vitvi07g04574\_t001 |  | \| \| |  |  |  |  |  |  |  |  |  |  |
| 1 | Vitvi07g01393\_t001 |  | \| \| |  |  |  |  |  |  |  |  |  |  |
| 1 | Vitvi07g01394\_t001 |  | Vitvi18g04082\_t001 |  |  |  |  |  |  |  |  |  |  |
| 1 | Vitvi07g02558\_t001 |  | \| \| |  |  |  |  |  |  |  |  |  |  |
| 1 | Vitvi07g03041\_t001 |  | Vitvi18g00420\_t001 |  |  |  |  |  |  |  |  |  |  |
| 1 | Vitvi07g02560\_t001 |  | \| \| |  |  |  |  |  |  |  |  |  |  |
| 1 | Vitvi07g01396\_t001 |  | Vitvi18g02581\_t001 |  |  |  |  |  |  |  |  |  |  |
| 1 | Vitvi07g03044\_t001 |  | \| \| |  |  |  |  |  |  |  |  |  |  |
| 1 | Vitvi07g04575\_t001 |  | \| \| |  |  |  |  |  |  |  |  |  |  |
| 1 | Vitvi07g04576\_t001 |  | \| \| |  |  |  |  |  |  |  |  |  |  |
| 1 | Vitvi07g03046\_t001 |  | Vitvi18g00435\_t002 |  |  |  |  |  |  |  |  |  |  |
| 1 | Vitvi07g04577\_t001 |  | \| \| |  |  |  |  |  |  |  |  |  |  |
| 1 | Vitvi07g04578\_t001 |  | \| \| |  |  |  |  |  |  |  |  |  |  |
| 1 | Vitvi07g03049\_t001 |  | Vitvi18g00436\_t001 |  |  |  |  |  |  |  |  |  |  |
| 1 | Vitvi07g04579\_t001 |  | \| \| |  |  |  |  |  |  |  |  |  |  |
| 1 | Vitvi07g04580\_t001 |  | \| \| |  |  |  |  |  |  |  |  |  |  |
| 1 | Vitvi07g04581\_t001 |  | \| \| |  |  |  |  |  |  |  |  |  |  |
| 1 | Vitvi07g04582\_t001 |  | \| \| |  |  |  |  |  |  |  |  |  |  |
| 1 | Vitvi07g03112\_t001 |  | \| \| |  |  |  |  |  |  |  |  |  |  |
| 1 | Vitvi07g02605\_t001 |  | \| \| |  |  |  |  |  |  |  |  |  |  |
| 1 | Vitvi07g02604\_t001 |  | \| \| |  |  |  |  |  |  |  |  |  |  |
| 1 | Vitvi07g04583\_t001 |  | \| \| |  |  |  |  |  |  |  |  |  |  |
| 1 | Vitvi07g01504\_t001 |  | \| \| |  |  |  |  |  |  |  |  |  |  |
| 1 | Vitvi07g04584\_t001 |  | \| \| |  |  |  |  |  |  |  |  |  |  |
| 1 | Vitvi07g01503\_t001 |  | \| \| |  |  |  |  |  |  |  |  |  |  |
| 1 | Vitvi07g04585\_t001 |  | \| \| |  |  |  |  |  |  |  |  |  |  |
| 1 | Vitvi07g01502\_t001 |  | \| \| |  |  |  |  |  |  |  |  |  |  |
| 1 | Vitvi07g01501\_t001 |  | Vitvi18g00438\_t001 |  |  |  |  |  |  |  |  |  |  |
| 1 | Vitvi07g01500\_t001 |  | Vitvi18g00440\_t001 |  |  |  |  |  |  |  |  |  |  |
| 1 | Vitvi07g01499\_t001 |  | \| \| |  |  |  |  |  |  |  |  |  |  |
| 1 | Vitvi07g04586\_t001 |  | \| \| |  |  |  |  |  |  |  |  |  |  |
| 1 | Vitvi07g01498\_t001 |  | Vitvi18g00442\_t001 |  |  |  |  |  |  |  |  |  |  |
| 1 | Vitvi07g04587\_t001 |  | \| \| |  |  |  |  |  |  |  |  |  |  |
| 1 | Vitvi07g01495\_t001 |  | \| \| |  |  |  |  |  |  |  |  |  |  |
| 1 | Vitvi07g01494\_t001 |  | \| \| |  |  |  |  |  |  |  |  |  |  |
| 1 | Vitvi07g01492\_t001 |  | \| \| |  |  |  |  |  |  |  |  |  |  |
| 1 | Vitvi07g01489\_t001 |  | \| \| |  |  |  |  |  |  |  |  |  |  |
| 1 | Vitvi07g04588\_t001 |  | \| \| |  |  |  |  |  |  |  |  |  |  |
| 1 | Vitvi07g01488\_t001 |  | Vitvi18g00448\_t001 |  |  |  |  |  |  |  |  |  |  |
| 1 | Vitvi07g01482\_t001 |  | \| \| |  |  |  |  |  |  |  |  |  |  |
| 1 | Vitvi07g01480\_t001 |  | \| \| |  |  |  |  |  |  |  |  |  |  |
| 1 | Vitvi07g02602\_t002 |  | Vitvi18g02589\_t002 |  |  |  |  |  |  |  |  |  |  |
| 1 | Vitvi07g01476\_t001 |  | Vitvi18g00462\_t001 |  |  |  |  |  |  |  |  |  |  |
| 1 | Vitvi07g01475\_t001 |  | Vitvi18g00463\_t001 |  |  |  |  |  |  |  |  |  |  |
| 1 | Vitvi07g01473\_t001 |  | \| \| |  |  |  |  |  |  |  |  |  |  |
| 1 | Vitvi07g02600\_t001 |  | \| \| |  |  |  |  |  |  |  |  |  |  |
| 1 | Vitvi07g01472\_t001 |  | \| \| |  |  |  |  |  |  |  |  |  |  |
| 1 | Vitvi07g04589\_t001 |  | \| \| |  |  |  |  |  |  |  |  |  |  |
| 1 | Vitvi07g04590\_t001 |  | Vitvi18g00464\_t001 |  |  |  |  |  |  |  |  |  |  |
| 1 | Vitvi07g01471\_t001 |  | Vitvi18g00469\_t001 |  |  |  |  |  |  |  |  |  |  |
| 1 | Vitvi07g04591\_t001 |  | Vitvi18g00470\_t001 |  |  |  |  |  |  |  |  |  |  |
| 1 | Vitvi07g01469\_t001 |  | Vitvi18g00488\_t001 |  |  |  |  |  |  |  |  |  |  |
| 1 | Vitvi07g02596\_t001 |  | \| \| |  |  |  |  |  |  |  |  |  |  |
| 1 | Vitvi07g01468\_t001 |  | Vitvi18g00489\_t001 |  |  |  |  |  |  |  |  |  |  |
| 1 | Vitvi07g04592\_t001 |  | Vitvi18g00492\_t001 |  |  |  |  |  |  |  |  |  |  |
| 1 | Vitvi07g04593\_t001 |  | \| \| |  |  |  |  |  |  |  |  |  |  |
| 1 | Vitvi07g04594\_t001 |  | \| \| |  |  |  |  |  |  |  |  |  |  |
| 1 | Vitvi07g01465\_t001 |  | Vitvi18g00493\_t001 |  |  |  |  |  |  |  |  |  |  |
| 1 | Vitvi07g02594\_t001 |  | \| \| |  |  |  |  |  |  |  |  |  |  |
| 1 | Vitvi07g01462\_t001 |  | \| \| |  |  |  |  |  |  |  |  |  |  |
| 1 | Vitvi07g01460\_t001 |  | Vitvi18g00499\_t001 |  |  |  |  |  |  |  |  |  |  |
| 1 | Vitvi07g04595\_t001 |  | \| \| |  |  |  |  |  |  |  |  |  |  |
| 1 | Vitvi07g01459\_t001 |  | \| \| |  |  |  |  |  |  |  |  |  |  |
| 1 | Vitvi07g04596\_t001 |  | \| \| |  |  |  |  |  |  |  |  |  |  |
| 1 | Vitvi07g01457\_t001 |  | Vitvi18g02600\_t001 |  |  |  |  |  |  |  |  |  |  |
| 1 | Vitvi07g02591\_t001 |  | \| \| |  |  |  |  |  |  |  |  |  |  |
| 1 | Vitvi07g02588\_t002 |  | \| \| |  |  |  |  |  |  |  |  |  |  |
| 1 | Vitvi07g02589\_t001 |  | \| \| |  |  |  |  |  |  |  |  |  |  |
| 1 | Vitvi07g01451\_t001 |  | Vitvi18g00505\_t002 |  |  |  |  |  |  |  |  |  |  |
| 1 | Vitvi07g04597\_t001 |  | \| \| |  |  |  |  |  |  |  |  |  |  |
| 1 | Vitvi07g04598\_t001 |  | \| \| |  |  |  |  |  |  |  |  |  |  |
| 1 | Vitvi07g04599\_t001 |  | Vitvi18g00507\_t001 |  |  |  |  |  |  |  |  |  |  |
| 1 | Vitvi07g04600\_t001 |  | Vitvi18g00508\_t001 |  |  |  |  |  |  |  |  |  |  |
| 1 | Vitvi07g01449\_t001 |  | Vitvi18g00511\_t001 |  |  |  |  |  |  |  |  |  |  |
| 1 | Vitvi07g02582\_t001 |  | \| \| |  |  |  |  |  |  |  |  |  |  |
| 1 | Vitvi07g02577\_t001 |  | Vitvi18g02607\_t001 |  |  |  |  |  |  |  |  |  |  |
| 1 | Vitvi07g04601\_t001 |  | \| \| |  |  |  |  |  |  |  |  |  |  |
| 1 | Vitvi07g02580\_t001 |  | \| \| |  |  |  |  |  |  |  |  |  |  |
| 1 | Vitvi07g04602\_t001 |  | \| \| |  |  |  |  |  |  |  |  |  |  |
| 1 | Vitvi07g04603\_t001 |  | Vitvi18g00513\_t001 |  |  |  |  |  |  |  |  |  |  |
| 1 | Vitvi07g01444\_t001 |  | Vitvi18g00516\_t001 |  |  |  |  |  |  |  |  |  |  |
| 1 | Vitvi07g04604\_t001 |  | \| \| |  |  |  |  |  |  |  |  |  |  |
| 1 | Vitvi07g01442\_t001 |  | \| \| |  |  |  |  |  |  |  |  |  |  |
| 1 | Vitvi07g04605\_t001 |  | \| \| |  |  |  |  |  |  |  |  |  |  |
| 1 | Vitvi07g01441\_t001 |  | Vitvi18g04114\_t001 |  |  |  |  |  |  |  |  |  |  |
| 1 | Vitvi07g04606\_t001 |  | \| \| |  |  |  |  |  |  |  |  |  |  |
| 1 | Vitvi07g01439\_t001 |  | \| \| |  |  |  |  |  |  |  |  |  |  |
| 1 | Vitvi07g01437\_t001 |  | Vitvi18g00518\_t002 |  |  |  |  |  |  |  |  |  |  |
| 1 | Vitvi07g04607\_t001 |  | Vitvi18g02610\_t001.1.6037826d |  |  |  |  |  |  |  |  |  |  |
| 1 | Vitvi07g01433\_t001 |  | \| \| |  |  |  |  |  |  |  |  |  |  |
| 1 | Vitvi07g01431\_t001 |  | Vitvi18g00524\_t001 |  |  |  |  |  |  |  |  |  |  |
| 1 | Vitvi07g04608\_t001 |  | Vitvi18g00525\_t001 |  |  |  |  |  |  |  |  |  |  |
| 1 | Vitvi07g04609\_t001 |  | \| \| |  |  |  |  |  |  |  |  |  |  |
| 1 | Vitvi07g01428\_t001 |  | \| \| |  |  |  |  |  |  |  |  |  |  |
| 1 | Vitvi07g01425\_t002 |  | \| \| |  |  |  |  |  |  |  |  |  |  |
| 1 | Vitvi07g04610\_t001 |  | \| \| |  |  |  |  |  |  |  |  |  |  |
| 1 | Vitvi07g04611\_t002 |  | \| \| |  |  |  |  |  |  |  |  |  |  |
| 1 | Vitvi07g01423\_t001 |  | \| \| |  |  |  |  |  |  |  |  |  |  |
| 1 | Vitvi07g01419\_t001 |  | \| \| |  |  |  |  |  |  |  |  |  |  |
| 1 | Vitvi07g02572\_t001 |  | Vitvi18g00533\_t001 |  |  |  |  |  |  |  |  |  |  |
| 1 | Vitvi07g04612\_t001 |  | \| \| |  |  |  |  |  |  |  |  |  |  |
| 1 | Vitvi07g04613\_t001 |  | \| \| |  |  |  |  |  |  |  |  |  |  |
| 1 | Vitvi07g04614\_t001 |  | \| \| |  |  |  |  |  |  |  |  |  |  |
| 1 | Vitvi07g04615\_t001 |  | \| \| |  |  |  |  |  |  |  |  |  |  |
| 1 | Vitvi07g04616\_t001 |  | \| \| |  |  |  |  |  |  |  |  |  |  |
| 1 | Vitvi07g04617\_t001 |  | \| \| |  |  |  |  |  |  |  |  |  |  |
| 1 | Vitvi07g01415\_t001 |  | \| \| |  |  |  |  |  |  |  |  |  |  |
| 1 | Vitvi07g04618\_t001 |  | \| \| |  |  |  |  |  |  |  |  |  |  |
| 1 | Vitvi07g04619\_t001 |  | \| \| |  |  |  |  |  |  |  |  |  |  |
| 1 | Vitvi07g01414\_t001 |  | \| \| |  |  |  |  |  |  |  |  |  |  |
| 1 | Vitvi07g02564\_t001 |  | \| \| |  |  |  |  |  |  |  |  |  |  |
| 1 | Vitvi07g04620\_t001 |  | \| \| |  |  |  |  |  |  |  |  |  |  |
| 1 | Vitvi07g01412\_t001 |  | \| \| |  |  |  |  |  |  |  |  |  |  |
| 1 | Vitvi07g01411\_t001 |  | \| \| |  |  |  |  |  |  |  |  |  |  |
| 1 | Vitvi07g02563\_t001 |  | \| \| |  |  |  |  |  |  |  |  |  |  |
| 1 | Vitvi07g01410\_t001 |  | \| \| |  |  |  |  |  |  |  |  |  |  |
| 1 | Vitvi07g04621\_t001 |  | \| \| |  |  |  |  |  |  |  |  |  |  |
| 1 | Vitvi07g01408\_t001 |  | \| \| |  |  |  |  |  |  |  |  |  |  |
| 1 | Vitvi07g01407\_t001 |  | \| \| |  |  |  |  |  |  |  |  |  |  |
| 1 | Vitvi07g01406\_t001 |  | \| \| |  |  |  |  |  |  |  |  |  |  |
| 1 | Vitvi07g01405\_t001 |  | \| \| |  |  |  |  |  |  |  |  |  |  |
| 1 | Vitvi07g01403\_t001 |  | \| \| |  |  |  |  |  |  |  |  |  |  |
| 1 | Vitvi07g01402\_t001 |  | Vitvi18g00544\_t001 |  |  |  |  |  |  |  |  |  |  |
| 1 | Vitvi07g04622\_t001 |  | \| \| |  |  |  |  |  |  |  |  |  |  |
| 1 | Vitvi07g01401\_t001 |  | \| \| |  |  |  |  |  |  |  |  |  |  |
| 1 | Vitvi07g01400\_t002 |  | \| \| |  |  |  |  |  |  |  |  |  |  |
| 1 | Vitvi07g04623\_t001 |  | \| \| |  |  |  |  |  |  |  |  |  |  |
| 1 | Vitvi07g04624\_t001 |  | \| \| |  |  |  |  |  |  |  |  |  |  |
| 1 | Vitvi07g04625\_t001 |  | \| \| |  |  |  |  |  |  |  |  |  |  |
| 1 | Vitvi07g01399\_t001 |  | \| \| |  |  |  |  |  |  |  |  |  |  |
| 1 | Vitvi07g02561\_t001 |  | \| \| |  |  |  |  |  |  |  |  |  |  |
| 1 | Vitvi07g02556\_t001 |  | \| \| |  |  |  |  |  |  |  |  |  |  |
| 1 | Vitvi07g02555\_t001 |  | \| \| |  |  |  |  |  |  |  |  |  |  |
| 1 | Vitvi07g04626\_t001 |  | \| \| |  |  |  |  |  |  |  |  |  |  |
| 1 | Vitvi07g02554\_t001 |  | \| \| |  |  |  |  |  |  |  |  |  |  |
| 1 | Vitvi07g01389\_t001 |  | \| \| |  |  |  |  |  |  |  |  |  |  |
| 1 | Vitvi07g02553\_t001 |  | \| \| |  |  |  |  |  |  |  |  |  |  |
| 1 | Vitvi07g01388\_t001 |  | Vitvi18g00549\_t001 |  |  |  |  |  |  |  |  |  |  |
| 2 | Vitvi07g01387\_t001 |  | \| \| |  | Vitvi03g01681\_t001 |  |  |  |  |  |  |  |  |
| 2 | Vitvi07g01386\_t001 |  | \| \| |  | \| \| |  |  |  |  |  |  |  |  |
| 2 | Vitvi07g01385\_t001 |  | \| \| |  | \| \| |  |  |  |  |  |  |  |  |
| 2 | Vitvi07g04627\_t001 |  | \| \| |  | Vitvi03g00813\_t001 |  |  |  |  |  |  |  |  |
| 2 | Vitvi07g04628\_t001 |  | \| \| |  | \| \| |  |  |  |  |  |  |  |  |
| 2 | Vitvi07g04629\_t001 |  | \| \| |  | \| \| |  |  |  |  |  |  |  |  |
| 2 | Vitvi07g04630\_t001 |  | \| \| |  | \| \| |  |  |  |  |  |  |  |  |
| 2 | Vitvi07g01520\_t001 |  | Vitvi18g00553\_t001 |  | Vitvi03g00819\_t001 |  |  |  |  |  |  |  |  |
| 2 | Vitvi07g04631\_t001 |  | \| \| |  | \| \| |  |  |  |  |  |  |  |  |
| 2 | Vitvi07g02610\_t001 |  | Vitvi18g00560\_t002 |  | \| \| |  |  |  |  |  |  |  |  |
| 2 | Vitvi07g04632\_t001 |  | \| \| |  | \| \| |  |  |  |  |  |  |  |  |
| 2 | Vitvi07g01516\_t001 |  | \| \| |  | \| \| |  |  |  |  |  |  |  |  |
| 2 | Vitvi07g04633\_t001 |  | \| \| |  | \| \| |  |  |  |  |  |  |  |  |
| 2 | Vitvi07g04634\_t001 |  | \| \| |  | \| \| |  |  |  |  |  |  |  |  |
| 2 | Vitvi07g02608\_t009 |  | \| \| |  | \| \| |  |  |  |  |  |  |  |  |
| 2 | Vitvi07g04635\_t001 |  | \| \| |  | \| \| |  |  |  |  |  |  |  |  |
| 2 | Vitvi07g01511\_t001 |  | \| \| |  | \| \| |  |  |  |  |  |  |  |  |
| 2 | Vitvi07g01510\_t001 |  | \| \| |  | \| \| |  |  |  |  |  |  |  |  |
| 2 | Vitvi07g04636\_t002 |  | \| \| |  | \| \| |  |  |  |  |  |  |  |  |
| 2 | Vitvi07g01508\_t001 |  | \| \| |  | \| \| |  |  |  |  |  |  |  |  |
| 2 | Vitvi07g01507\_t001 |  | \| \| |  | \| \| |  |  |  |  |  |  |  |  |
| 2 | Vitvi07g01506\_t001 |  | \| \| |  | \| \| |  |  |  |  |  |  |  |  |
| 2 | Vitvi07g01524\_t001 |  | \| \| |  | Vitvi03g00845\_t001 |  |  |  |  |  |  |  |  |
| 2 | Vitvi07g01523\_t001 |  | Vitvi18g00562\_t001 |  | \| \| |  |  |  |  |  |  |  |  |
| 2 | Vitvi07g04637\_t001 |  | \| \| |  | Vitvi03g00847\_t003 |  |  |  |  |  |  |  |  |
| 2 | Vitvi07g04638\_t001 |  | \| \| |  | \| \| |  |  |  |  |  |  |  |  |
| 2 | Vitvi07g01522\_t001 |  | \| \| |  | \| \| |  |  |  |  |  |  |  |  |
| 2 | Vitvi07g02613\_t001 |  | Vitvi18g00567\_t001 |  | \| \| |  |  |  |  |  |  |  |  |
| 2 | Vitvi07g01543\_t001 |  | Vitvi18g04122\_t001 |  | \| \| |  |  |  |  |  |  |  |  |
| 2 | Vitvi07g04639\_t001 |  | \| \| |  | \| \| |  |  |  |  |  |  |  |  |
| 2 | Vitvi07g04640\_t001 |  | \| \| |  | \| \| |  |  |  |  |  |  |  |  |
| 2 | Vitvi07g04641\_t001 |  | \| \| |  | \| \| |  |  |  |  |  |  |  |  |
| 2 | Vitvi07g04642\_t003 |  | Vitvi18g00571\_t003 |  | \| \| |  |  |  |  |  |  |  |  |
| 2 | Vitvi07g04643\_t001 |  | \| \| |  | \| \| |  |  |  |  |  |  |  |  |
| 2 | Vitvi07g04644\_t001 |  | Vitvi18g00572\_t001 |  | \| \| |  |  |  |  |  |  |  |  |
| 2 | Vitvi07g01541\_t001 |  | \| \| |  | Vitvi03g00855\_t004 |  |  |  |  |  |  |  |  |
| 2 | Vitvi07g04645\_t001 |  | \| \| |  | \| \| |  |  |  |  |  |  |  |  |
| 2 | Vitvi07g04646\_t001 |  | \| \| |  | \| \| |  |  |  |  |  |  |  |  |
| 2 | Vitvi07g04647\_t001 |  | \| \| |  | \| \| |  |  |  |  |  |  |  |  |
| 2 | Vitvi07g01538\_t001 |  | \| \| |  | \| \| |  |  |  |  |  |  |  |  |
| 2 | Vitvi07g04648\_t001 |  | \| \| |  | \| \| |  |  |  |  |  |  |  |  |
| 2 | Vitvi07g01536\_t001 |  | \| \| |  | Vitvi03g04288\_t001 |  |  |  |  |  |  |  |  |
| 1 | Vitvi07g01535\_t001 |  | \| \| |  |  |  |  |  |  |  |  |  |  |
| 1 | Vitvi07g01534\_t001 |  | \| \| |  |  |  |  |  |  |  |  |  |  |
| 1 | Vitvi07g01533\_t001 |  | \| \| |  |  |  |  |  |  |  |  |  |  |
| 1 | Vitvi07g01532\_t001 |  | \| \| |  |  |  |  |  |  |  |  |  |  |
| 1 | Vitvi07g01531\_t001 |  | \| \| |  |  |  |  |  |  |  |  |  |  |
| 1 | Vitvi07g01530\_t001 |  | \| \| |  |  |  |  |  |  |  |  |  |  |
| 1 | Vitvi07g01529\_t001 |  | \| \| |  |  |  |  |  |  |  |  |  |  |
| 1 | Vitvi07g04649\_t001 |  | \| \| |  |  |  |  |  |  |  |  |  |  |
| 1 | Vitvi07g03072\_t001 |  | \| \| |  |  |  |  |  |  |  |  |  |  |
| 1 | Vitvi07g04650\_t001 |  | \| \| |  |  |  |  |  |  |  |  |  |  |
| 1 | Vitvi07g04651\_t001 |  | \| \| |  |  |  |  |  |  |  |  |  |  |
| 1 | Vitvi07g04652\_t001 |  | \| \| |  |  |  |  |  |  |  |  |  |  |
| 1 | Vitvi07g04653\_t001 |  | \| \| |  |  |  |  |  |  |  |  |  |  |
| 1 | Vitvi07g03070\_t001 |  | \| \| |  |  |  |  |  |  |  |  |  |  |
| 1 | Vitvi07g04654\_t001 |  | \| \| |  |  |  |  |  |  |  |  |  |  |
| 1 | Vitvi07g04655\_t001 |  | Vitvi18g00587\_t001 |  |  |  |  |  |  |  |  |  |  |
| 1 | Vitvi07g04656\_t001 |  | Vitvi18g00590\_t001 |  |  |  |  |  |  |  |  |  |  |
| 1 | Vitvi07g04657\_t001 |  | \| \| |  |  |  |  |  |  |  |  |  |  |
| 1 | Vitvi07g04658\_t001 |  | \| \| |  |  |  |  |  |  |  |  |  |  |
| 1 | Vitvi07g04659\_t001 |  | \| \| |  |  |  |  |  |  |  |  |  |  |
| 1 | Vitvi07g03065\_t001 |  | \| \| |  |  |  |  |  |  |  |  |  |  |
| 1 | Vitvi07g03064\_t001 |  | \| \| |  |  |  |  |  |  |  |  |  |  |
| 1 | Vitvi07g03063\_t002 |  | Vitvi18g02621\_t001 |  |  |  |  |  |  |  |  |  |  |
| 1 | Vitvi07g04660\_t001 |  | \| \| |  |  |  |  |  |  |  |  |  |  |
| 1 | Vitvi07g03061\_t001 |  | Vitvi18g02622\_t001 |  |  |  |  |  |  |  |  |  |  |
| 1 | Vitvi07g04661\_t001 |  | \| \| |  |  |  |  |  |  |  |  |  |  |
| 1 | Vitvi07g04662\_t001 |  | \| \| |  |  |  |  |  |  |  |  |  |  |
| 1 | Vitvi07g03056\_t002 |  | Vitvi18g00603\_t001 |  |  |  |  |  |  |  |  |  |  |
| 1 | Vitvi07g03099\_t001 |  | \| \| |  |  |  |  |  |  |  |  |  |  |
| 1 | Vitvi07g04663\_t001 |  | \| \| |  |  |  |  |  |  |  |  |  |  |
| 1 | Vitvi07g04664\_t001 |  | \| \| |  |  |  |  |  |  |  |  |  |  |
| 1 | Vitvi07g03132\_t001 |  | \| \| |  |  |  |  |  |  |  |  |  |  |
| 1 | Vitvi07g03055\_t001 |  | Vitvi18g00605\_t001 |  |  |  |  |  |  |  |  |  |  |
| 0 | Vitvi07g04665\_t001 |  |  |  |  |  |  |  |  |  |  |  |  |
| 0 | Vitvi07g04666\_t001 |  |  |  |  |  |  |  |  |  |  |  |  |
| 0 | Vitvi07g04667\_t001 |  |  |  |  |  |  |  |  |  |  |  |  |
| 0 | Vitvi07g04668\_t001 |  |  |  |  |  |  |  |  |  |  |  |  |
| 0 | Vitvi07g04669\_t001 |  |  |  |  |  |  |  |  |  |  |  |  |
| 0 | Vitvi07g01570\_t001 |  |  |  |  |  |  |  |  |  |  |  |  |
| 1 | Vitvi07g04670\_t004 |  | Vitvi03g00668\_t001 |  |  |  |  |  |  |  |  |  |  |
| 1 | Vitvi07g01572\_t001 |  | \| \| |  |  |  |  |  |  |  |  |  |  |
| 1 | Vitvi07g04671\_t001 |  | \| \| |  |  |  |  |  |  |  |  |  |  |
| 1 | Vitvi07g01577\_t001 |  | Vitvi03g00665\_t001 |  |  |  |  |  |  |  |  |  |  |
| 1 | Vitvi07g01578\_t001 |  | \| \| |  |  |  |  |  |  |  |  |  |  |
| 1 | Vitvi07g01580\_t002 |  | \| \| |  |  |  |  |  |  |  |  |  |  |
| 1 | Vitvi07g02073\_t001 |  | \| \| |  |  |  |  |  |  |  |  |  |  |
| 1 | Vitvi07g04672\_t001 |  | \| \| |  |  |  |  |  |  |  |  |  |  |
| 1 | Vitvi07g01581\_t001 |  | \| \| |  |  |  |  |  |  |  |  |  |  |
| 1 | Vitvi07g02633\_t001 |  | \| \| |  |  |  |  |  |  |  |  |  |  |
| 1 | Vitvi07g01590\_t001 |  | Vitvi03g00651\_t001 |  |  |  |  |  |  |  |  |  |  |
| 1 | Vitvi07g01591\_t001 |  | \| \| |  |  |  |  |  |  |  |  |  |  |
| 1 | Vitvi07g04673\_t001 |  | \| \| |  |  |  |  |  |  |  |  |  |  |
| 2 | Vitvi07g01593\_t001 |  | \| \| |  | Vitvi18g00664\_t001 |  |  |  |  |  |  |  |  |
| 2 | Vitvi07g01595\_t001 |  | \| \| |  | Vitvi18g00668\_t003 |  |  |  |  |  |  |  |  |
| 2 | Vitvi07g04674\_t001 |  | \| \| |  | \| \| |  |  |  |  |  |  |  |  |
| 2 | Vitvi07g04675\_t001 |  | \| \| |  | Vitvi18g02649\_t001 |  |  |  |  |  |  |  |  |
| 2 | Vitvi07g01598\_t001 |  | \| \| |  | \| \| |  |  |  |  |  |  |  |  |
| 2 | Vitvi07g01599\_t001 |  | \| \| |  | \| \| |  |  |  |  |  |  |  |  |
| 2 | Vitvi07g01600\_t001 |  | \| \| |  | \| \| |  |  |  |  |  |  |  |  |
| 2 | Vitvi07g04676\_t001 |  | \| \| |  | \| \| |  |  |  |  |  |  |  |  |
| 2 | Vitvi07g01602\_t001 |  | Vitvi03g00630\_t001 |  | Vitvi18g00675\_t001 |  |  |  |  |  |  |  |  |
| 2 | Vitvi07g01603\_t003 |  | Vitvi03g00629\_t001 |  | \| \| |  |  |  |  |  |  |  |  |
| 2 | Vitvi07g01604\_t001 |  | \| \| |  | \| \| |  |  |  |  |  |  |  |  |
| 2 | Vitvi07g04677\_t001 |  | \| \| |  | \| \| |  |  |  |  |  |  |  |  |
| 2 | Vitvi07g01605\_t001 |  | \| \| |  | \| \| |  |  |  |  |  |  |  |  |
| 2 | Vitvi07g04678\_t001 |  | \| \| |  | \| \| |  |  |  |  |  |  |  |  |
| 2 | Vitvi07g01606\_t001 |  | \| \| |  | \| \| |  |  |  |  |  |  |  |  |
| 2 | Vitvi07g01608\_t001 |  | \| \| |  | \| \| |  |  |  |  |  |  |  |  |
| 2 | Vitvi07g04679\_t001 |  | \| \| |  | \| \| |  |  |  |  |  |  |  |  |
| 2 | Vitvi07g02635\_t001 |  | \| \| |  | \| \| |  |  |  |  |  |  |  |  |
| 2 | Vitvi07g04680\_t001 |  | \| \| |  | \| \| |  |  |  |  |  |  |  |  |
| 2 | Vitvi07g01610\_t001 |  | Vitvi03g00628\_t001 |  | Vitvi18g00677\_t001 |  |  |  |  |  |  |  |  |
| 2 | Vitvi07g01612\_t001 |  | \| \| |  | \| \| |  |  |  |  |  |  |  |  |
| 2 | Vitvi07g01613\_t001 |  | Vitvi03g00618\_t001 |  | \| \| |  |  |  |  |  |  |  |  |
| 2 | Vitvi07g01614\_t001 |  | \| \| |  | \| \| |  |  |  |  |  |  |  |  |
| 2 | Vitvi07g04681\_t001 |  | \| \| |  | \| \| |  |  |  |  |  |  |  |  |
| 2 | Vitvi07g01616\_t001 |  | \| \| |  | \| \| |  |  |  |  |  |  |  |  |
| 2 | Vitvi07g01617\_t001 |  | \| \| |  | \| \| |  |  |  |  |  |  |  |  |
| 2 | Vitvi07g01619\_t001 |  | \| \| |  | \| \| |  |  |  |  |  |  |  |  |
| 2 | Vitvi07g01620\_t002 |  | \| \| |  | Vitvi18g00681\_t001 |  |  |  |  |  |  |  |  |
| 2 | Vitvi07g01621\_t001 |  | \| \| |  | Vitvi18g00682\_t001 |  |  |  |  |  |  |  |  |
| 2 | Vitvi07g01622\_t001 |  | \| \| |  | Vitvi18g04157\_t001 |  |  |  |  |  |  |  |  |
| 2 | Vitvi07g04682\_t001 |  | \| \| |  | \| \| |  |  |  |  |  |  |  |  |
| 2 | Vitvi07g01623\_t001 |  | \| \| |  | \| \| |  |  |  |  |  |  |  |  |
| 2 | Vitvi07g01624\_t001 |  | Vitvi03g00609\_t001 |  | \| \| |  |  |  |  |  |  |  |  |
| 2 | Vitvi07g01625\_t001 |  | \| \| |  | \| \| |  |  |  |  |  |  |  |  |
| 2 | Vitvi07g01626\_t001 |  | \| \| |  | \| \| |  |  |  |  |  |  |  |  |
| 2 | Vitvi07g01627\_t001 |  | \| \| |  | Vitvi18g00683\_t001 |  |  |  |  |  |  |  |  |
| 2 | Vitvi07g04683\_t001 |  | \| \| |  | \| \| |  |  |  |  |  |  |  |  |
| 2 | Vitvi07g01630\_t001 |  | \| \| |  | \| \| |  |  |  |  |  |  |  |  |
| 2 | Vitvi07g02637\_t001 |  | \| \| |  | \| \| |  |  |  |  |  |  |  |  |
| 2 | Vitvi07g01631\_t001 |  | Vitvi03g00605\_t001 |  | Vitvi18g00686\_t001 |  |  |  |  |  |  |  |  |
| 2 | Vitvi07g01633\_t001 |  | \| \| |  | \| \| |  |  |  |  |  |  |  |  |
| 2 | Vitvi07g01634\_t001 |  | \| \| |  | \| \| |  |  |  |  |  |  |  |  |
| 2 | Vitvi07g01635\_t001 |  | \| \| |  | Vitvi18g00689\_t001 |  |  |  |  |  |  |  |  |
| 2 | Vitvi07g01636\_t001 |  | \| \| |  | \| \| |  |  |  |  |  |  |  |  |
| 2 | Vitvi07g01637\_t001 |  | Vitvi03g00595\_t002 |  | \| \| |  |  |  |  |  |  |  |  |
| 2 | Vitvi07g01638\_t001 |  | Vitvi03g00593\_t001 |  | Vitvi18g00691\_t001 |  |  |  |  |  |  |  |  |
| 2 | Vitvi07g04684\_t001 |  | \| \| |  | Vitvi18g00693\_t001 |  |  |  |  |  |  |  |  |
| 2 | Vitvi07g01641\_t002 |  | \| \| |  | \| \| |  |  |  |  |  |  |  |  |
| 2 | Vitvi07g01642\_t001 |  | \| \| |  | Vitvi18g00695\_t001 |  |  |  |  |  |  |  |  |
| 2 | Vitvi07g04685\_t001 |  | \| \| |  | Vitvi18g00699\_t001 |  |  |  |  |  |  |  |  |
| 2 | Vitvi07g04686\_t001 |  | \| \| |  | Vitvi18g00700\_t001 |  |  |  |  |  |  |  |  |
| 2 | Vitvi07g01644\_t001 |  | Vitvi03g00586\_t001 |  | \| \| |  |  |  |  |  |  |  |  |
| 2 | Vitvi07g01645\_t001 |  | Vitvi03g00582\_t001 |  | Vitvi18g00708\_t001 |  |  |  |  |  |  |  |  |
| 1 | Vitvi07g01646\_t001 |  |  |  | \| \| |  |  |  |  |  |  |  |  |
| 1 | Vitvi07g01647\_t003 |  |  |  | Vitvi18g00711\_t001 |  |  |  |  |  |  |  |  |
| 1 | Vitvi07g04687\_t001 |  |  |  | \| \| |  |  |  |  |  |  |  |  |
| 1 | Vitvi07g01648\_t001 |  |  |  | \| \| |  |  |  |  |  |  |  |  |
| 1 | Vitvi07g01650\_t001 |  |  |  | \| \| |  |  |  |  |  |  |  |  |
| 1 | Vitvi07g04688\_t001 |  |  |  | \| \| |  |  |  |  |  |  |  |  |
| 1 | Vitvi07g04689\_t001 |  |  |  | \| \| |  |  |  |  |  |  |  |  |
| 1 | Vitvi07g04690\_t001 |  |  |  | \| \| |  |  |  |  |  |  |  |  |
| 1 | Vitvi07g02640\_t001 |  |  |  | \| \| |  |  |  |  |  |  |  |  |
| 1 | Vitvi07g04691\_t001 |  |  |  | \| \| |  |  |  |  |  |  |  |  |
| 1 | Vitvi07g04692\_t001 |  |  |  | \| \| |  |  |  |  |  |  |  |  |
| 1 | Vitvi07g01657\_t001 |  |  |  | Vitvi18g00716\_t001 |  |  |  |  |  |  |  |  |
| 1 | Vitvi07g04693\_t001 |  |  |  | \| \| |  |  |  |  |  |  |  |  |
| 1 | Vitvi07g04694\_t001 |  |  |  | \| \| |  |  |  |  |  |  |  |  |
| 1 | Vitvi07g01658\_t001 |  |  |  | \| \| |  |  |  |  |  |  |  |  |
| 1 | Vitvi07g04695\_t001 |  |  |  | \| \| |  |  |  |  |  |  |  |  |
| 1 | Vitvi07g04696\_t001 |  |  |  | \| \| |  |  |  |  |  |  |  |  |
| 1 | Vitvi07g02642\_t001 |  |  |  | \| \| |  |  |  |  |  |  |  |  |
| 1 | Vitvi07g01662\_t001 |  |  |  | Vitvi18g02659\_t001 |  |  |  |  |  |  |  |  |
| 1 | Vitvi07g02643\_t001 |  |  |  | \| \| |  |  |  |  |  |  |  |  |
| 1 | Vitvi07g01663\_t001 |  |  |  | \| \| |  |  |  |  |  |  |  |  |
| 1 | Vitvi07g01665\_t001 |  |  |  | \| \| |  |  |  |  |  |  |  |  |
| 1 | Vitvi07g04697\_t001 |  |  |  | \| \| |  |  |  |  |  |  |  |  |
| 1 | Vitvi07g01667\_t001 |  |  |  | \| \| |  |  |  |  |  |  |  |  |
| 1 | Vitvi07g01669\_t001 |  |  |  | \| \| |  |  |  |  |  |  |  |  |
| 1 | Vitvi07g04698\_t001 |  |  |  | \| \| |  |  |  |  |  |  |  |  |
| 1 | Vitvi07g02644\_t001 |  |  |  | \| \| |  |  |  |  |  |  |  |  |
| 1 | Vitvi07g02645\_t001 |  |  |  | \| \| |  |  |  |  |  |  |  |  |
| 1 | Vitvi07g04699\_t001 |  |  |  | \| \| |  |  |  |  |  |  |  |  |
| 1 | Vitvi07g02647\_t001 |  |  |  | \| \| |  |  |  |  |  |  |  |  |
| 1 | Vitvi07g01670\_t001 |  |  |  | \| \| |  |  |  |  |  |  |  |  |
| 1 | Vitvi07g02648\_t001 |  |  |  | \| \| |  |  |  |  |  |  |  |  |
| 1 | Vitvi07g02649\_t001 |  |  |  | \| \| |  |  |  |  |  |  |  |  |
| 1 | Vitvi07g04700\_t001 |  |  |  | \| \| |  |  |  |  |  |  |  |  |
| 1 | Vitvi07g01671\_t001 |  |  |  | Vitvi18g00720\_t001 |  |  |  |  |  |  |  |  |
| 2 | Vitvi07g02650\_t001 |  | Vitvi03g04192\_t001 |  | \| \| |  |  |  |  |  |  |  |  |
| 2 | Vitvi07g01672\_t001 |  | \| \| |  | Vitvi18g00721\_t002 |  |  |  |  |  |  |  |  |
| 2 | Vitvi07g01673\_t001 |  | Vitvi03g00561\_t001 |  | \| \| |  |  |  |  |  |  |  |  |
| 2 | Vitvi07g04701\_t001 |  | \| \| |  | \| \| |  |  |  |  |  |  |  |  |
| 2 | Vitvi07g01674\_t001 |  | \| \| |  | \| \| |  |  |  |  |  |  |  |  |
| 2 | Vitvi07g01676\_t001 |  | Vitvi03g00559\_t001 |  | Vitvi18g00725\_t001 |  |  |  |  |  |  |  |  |
| 2 | Vitvi07g04702\_t001 |  | \| \| |  | \| \| |  |  |  |  |  |  |  |  |
| 2 | Vitvi07g04703\_t001 |  | \| \| |  | \| \| |  |  |  |  |  |  |  |  |
| 2 | Vitvi07g01677\_t001 |  | \| \| |  | \| \| |  |  |  |  |  |  |  |  |
| 2 | Vitvi07g01678\_t001 |  | Vitvi03g00553\_t001 |  | \| \| |  |  |  |  |  |  |  |  |
| 2 | Vitvi07g01679\_t001 |  | \| \| |  | \| \| |  |  |  |  |  |  |  |  |
| 2 | Vitvi07g04704\_t001 |  | \| \| |  | \| \| |  |  |  |  |  |  |  |  |
| 2 | Vitvi07g01681\_t001 |  | \| \| |  | \| \| |  |  |  |  |  |  |  |  |
| 2 | Vitvi07g01682\_t002 |  | \| \| |  | \| \| |  |  |  |  |  |  |  |  |
| 2 | Vitvi07g01683\_t001 |  | Vitvi03g00542\_t001 |  | Vitvi18g00731\_t001 |  |  |  |  |  |  |  |  |
| 2 | Vitvi07g01684\_t001 |  | \| \| |  | Vitvi18g00734\_t001 |  |  |  |  |  |  |  |  |
| 2 | Vitvi07g01685\_t001 |  | \| \| |  | \| \| |  |  |  |  |  |  |  |  |
| 2 | Vitvi07g01686\_t001 |  | Vitvi03g00540\_t001 |  | \| \| |  |  |  |  |  |  |  |  |
| 2 | Vitvi07g04705\_t001 |  | \| \| |  | \| \| |  |  |  |  |  |  |  |  |
| 2 | Vitvi07g01688\_t001 |  | \| \| |  | \| \| |  |  |  |  |  |  |  |  |
| 2 | Vitvi07g01689\_t001 |  | \| \| |  | \| \| |  |  |  |  |  |  |  |  |
| 2 | Vitvi07g04706\_t001 |  | \| \| |  | \| \| |  |  |  |  |  |  |  |  |
| 2 | Vitvi07g01690\_t001 |  | Vitvi03g00539\_t001 |  | Vitvi18g00740\_t002 |  |  |  |  |  |  |  |  |
| 2 | Vitvi07g01692\_t001 |  | \| \| |  | \| \| |  |  |  |  |  |  |  |  |
| 2 | Vitvi07g01694\_t001 |  | \| \| |  | Vitvi18g00742\_t001 |  |  |  |  |  |  |  |  |
| 2 | Vitvi07g01695\_t001 |  | \| \| |  | \| \| |  |  |  |  |  |  |  |  |
| 2 | Vitvi07g01696\_t002 |  | \| \| |  | \| \| |  |  |  |  |  |  |  |  |
| 2 | Vitvi07g01697\_t001 |  | Vitvi03g00535\_t001 |  | \| \| |  |  |  |  |  |  |  |  |
| 2 | Vitvi07g01698\_t001 |  | \| \| |  | Vitvi18g00747\_t001 |  |  |  |  |  |  |  |  |
| 2 | Vitvi07g01699\_t002 |  | \| \| |  | Vitvi18g00749\_t002 |  |  |  |  |  |  |  |  |
| 2 | Vitvi07g01700\_t001 |  | \| \| |  | Vitvi18g00752\_t001 |  |  |  |  |  |  |  |  |
| 2 | Vitvi07g01701\_t002 |  | \| \| |  | \| \| |  |  |  |  |  |  |  |  |
| 2 | Vitvi07g01702\_t001 |  | \| \| |  | Vitvi18g04174\_t001 |  |  |  |  |  |  |  |  |
| 2 | Vitvi07g01703\_t001 |  | Vitvi03g00532\_t001 |  | \| \| |  |  |  |  |  |  |  |  |
| 2 | Vitvi07g01705\_t001 |  | Vitvi03g00531\_t001 |  | Vitvi18g00758\_t001 |  |  |  |  |  |  |  |  |
| 2 | Vitvi07g01706\_t001 |  | \| \| |  | \| \| |  |  |  |  |  |  |  |  |
| 2 | Vitvi07g01707\_t002 |  | Vitvi03g00530\_t002 |  | \| \| |  |  |  |  |  |  |  |  |
| 2 | Vitvi07g01708\_t001 |  | \| \| |  | \| \| |  |  |  |  |  |  |  |  |
| 2 | Vitvi07g01709\_t001 |  | Vitvi03g00529\_t002 |  | \| \| |  |  |  |  |  |  |  |  |
| 2 | Vitvi07g01710\_t001 |  | Vitvi03g00528\_t001 |  | \| \| |  |  |  |  |  |  |  |  |
| 2 | Vitvi07g04707\_t001 |  | \| \| |  | Vitvi18g00759\_t001 |  |  |  |  |  |  |  |  |
| 2 | Vitvi07g04708\_t001 |  | \| \| |  | \| \| |  |  |  |  |  |  |  |  |
| 2 | Vitvi07g01711\_t001 |  | \| \| |  | \| \| |  |  |  |  |  |  |  |  |
| 2 | Vitvi07g04709\_t001 |  | \| \| |  | \| \| |  |  |  |  |  |  |  |  |
| 2 | Vitvi07g01713\_t001 |  | \| \| |  | \| \| |  |  |  |  |  |  |  |  |
| 2 | Vitvi07g01714\_t001 |  | \| \| |  | Vitvi18g04177\_t001 |  |  |  |  |  |  |  |  |
| 2 | Vitvi07g04710\_t001 |  | \| \| |  | \| \| |  |  |  |  |  |  |  |  |
| 2 | Vitvi07g01718\_t001 |  | \| \| |  | \| \| |  |  |  |  |  |  |  |  |
| 2 | Vitvi07g01719\_t001 |  | \| \| |  | \| \| |  |  |  |  |  |  |  |  |
| 2 | Vitvi07g04711\_t001 |  | \| \| |  | \| \| |  |  |  |  |  |  |  |  |
| 2 | Vitvi07g04712\_t001 |  | \| \| |  | \| \| |  |  |  |  |  |  |  |  |
| 2 | Vitvi07g01721\_t001 |  | \| \| |  | \| \| |  |  |  |  |  |  |  |  |
| 2 | Vitvi07g01722\_t001 |  | \| \| |  | \| \| |  |  |  |  |  |  |  |  |
| 2 | Vitvi07g02656\_t001 |  | \| \| |  | \| \| |  |  |  |  |  |  |  |  |
| 2 | Vitvi07g01723\_t001 |  | Vitvi03g00524\_t001 |  | \| \| |  |  |  |  |  |  |  |  |
| 2 | Vitvi07g01724\_t001 |  | \| \| |  | \| \| |  |  |  |  |  |  |  |  |
| 2 | Vitvi07g02658\_t001 |  | \| \| |  | \| \| |  |  |  |  |  |  |  |  |
| 2 | Vitvi07g04713\_t001 |  | Vitvi03g00521\_t001 |  | \| \| |  |  |  |  |  |  |  |  |
| 2 | Vitvi07g02660\_t001 |  | \| \| |  | \| \| |  |  |  |  |  |  |  |  |
| 2 | Vitvi07g02661\_t001 |  | \| \| |  | \| \| |  |  |  |  |  |  |  |  |
| 2 | Vitvi07g04714\_t001 |  | \| \| |  | \| \| |  |  |  |  |  |  |  |  |
| 2 | Vitvi07g04715\_t001 |  | \| \| |  | \| \| |  |  |  |  |  |  |  |  |
| 2 | Vitvi07g02662\_t001 |  | \| \| |  | \| \| |  |  |  |  |  |  |  |  |
| 2 | Vitvi07g01728\_t001 |  | \| \| |  | Vitvi18g00762\_t001 |  |  |  |  |  |  |  |  |
| 2 | Vitvi07g01729\_t001 |  | \| \| |  | Vitvi18g00763\_t001 |  |  |  |  |  |  |  |  |
| 2 | Vitvi07g01730\_t002 |  | \| \| |  | \| \| |  |  |  |  |  |  |  |  |
| 2 | Vitvi07g01731\_t001 |  | \| \| |  | \| \| |  |  |  |  |  |  |  |  |
| 2 | Vitvi07g01732\_t001 |  | \| \| |  | Vitvi18g00768\_t002 |  |  |  |  |  |  |  |  |
| 2 | Vitvi07g01733\_t001 |  | Vitvi03g00517\_t001 |  | \| \| |  |  |  |  |  |  |  |  |
| 2 | Vitvi07g04716\_t001 |  | \| \| |  | \| \| |  |  |  |  |  |  |  |  |
| 2 | Vitvi07g01734\_t001 |  | \| \| |  | Vitvi18g00770\_t001 |  |  |  |  |  |  |  |  |
| 2 | Vitvi07g01735\_t001 |  | \| \| |  | \| \| |  |  |  |  |  |  |  |  |
| 2 | Vitvi07g01736\_t001 |  | \| \| |  | Vitvi18g00775\_t001 |  |  |  |  |  |  |  |  |
| 2 | Vitvi07g01737\_t001 |  | \| \| |  | \| \| |  |  |  |  |  |  |  |  |
| 2 | Vitvi07g01739\_t001 |  | \| \| |  | \| \| |  |  |  |  |  |  |  |  |
| 2 | Vitvi07g01740\_t001 |  | \| \| |  | Vitvi18g00779\_t001 |  |  |  |  |  |  |  |  |
| 2 | Vitvi07g02664\_t001 |  | \| \| |  | Vitvi18g00780\_t001 |  |  |  |  |  |  |  |  |
| 2 | Vitvi07g02665\_t001 |  | \| \| |  | \| \| |  |  |  |  |  |  |  |  |
| 2 | Vitvi07g01742\_t002 |  | \| \| |  | \| \| |  |  |  |  |  |  |  |  |
| 2 | Vitvi07g01743\_t004 |  | \| \| |  | \| \| |  |  |  |  |  |  |  |  |
| 2 | Vitvi07g01744\_t001 |  | \| \| |  | Vitvi18g00781\_t002 |  |  |  |  |  |  |  |  |
| 2 | Vitvi07g01745\_t001 |  | \| \| |  | \| \| |  |  |  |  |  |  |  |  |
| 2 | Vitvi07g01746\_t001 |  | \| \| |  | \| \| |  |  |  |  |  |  |  |  |
| 2 | Vitvi07g01747\_t001 |  | \| \| |  | \| \| |  |  |  |  |  |  |  |  |
| 2 | Vitvi07g02666\_t001 |  | \| \| |  | Vitvi18g00785\_t001 |  |  |  |  |  |  |  |  |
| 2 | Vitvi07g01748\_t001 |  | \| \| |  | \| \| |  |  |  |  |  |  |  |  |
| 2 | Vitvi07g01749\_t001 |  | \| \| |  | \| \| |  |  |  |  |  |  |  |  |
| 2 | Vitvi07g01750\_t001 |  | \| \| |  | \| \| |  |  |  |  |  |  |  |  |
| 2 | Vitvi07g01751\_t001 |  | Vitvi03g00508\_t001 |  | Vitvi18g00792\_t001 |  |  |  |  |  |  |  |  |
| 2 | Vitvi07g01752\_t001 |  | \| \| |  | \| \| |  |  |  |  |  |  |  |  |
| 2 | Vitvi07g01753\_t001 |  | \| \| |  | Vitvi18g00795\_t001 |  |  |  |  |  |  |  |  |
| 2 | Vitvi07g04717\_t001 |  | \| \| |  | \| \| |  |  |  |  |  |  |  |  |
| 2 | Vitvi07g04718\_t001 |  | Vitvi03g00500\_t001 |  | \| \| |  |  |  |  |  |  |  |  |
| 2 | Vitvi07g04719\_t001 |  | \| \| |  | \| \| |  |  |  |  |  |  |  |  |
| 2 | Vitvi07g01755\_t001 |  | \| \| |  | \| \| |  |  |  |  |  |  |  |  |
| 2 | Vitvi07g02069\_t001 |  | \| \| |  | \| \| |  |  |  |  |  |  |  |  |
| 2 | Vitvi07g01756\_t001 |  | \| \| |  | \| \| |  |  |  |  |  |  |  |  |
| 2 | Vitvi07g04720\_t001 |  | \| \| |  | \| \| |  |  |  |  |  |  |  |  |
| 2 | Vitvi07g01757\_t001 |  | \| \| |  | \| \| |  |  |  |  |  |  |  |  |
| 2 | Vitvi07g01759\_t001 |  | \| \| |  | \| \| |  |  |  |  |  |  |  |  |
| 2 | Vitvi07g01760\_t001 |  | \| \| |  | Vitvi18g02689\_t001 |  |  |  |  |  |  |  |  |
| 2 | Vitvi07g01761\_t001 |  | Vitvi03g00493\_t001 |  | \| \| |  |  |  |  |  |  |  |  |
| 2 | Vitvi07g01762\_t001 |  | Vitvi03g04176\_t001 |  | Vitvi18g00807\_t001 |  |  |  |  |  |  |  |  |
| 2 | Vitvi07g01763\_t001 |  | \| \| |  | \| \| |  |  |  |  |  |  |  |  |
| 2 | Vitvi07g02667\_t002 |  | Vitvi03g01562\_t001 |  | \| \| |  |  |  |  |  |  |  |  |
| 2 | Vitvi07g01765\_t001 |  | \| \| |  | \| \| |  |  |  |  |  |  |  |  |
| 2 | Vitvi07g01766\_t001 |  | \| \| |  | Vitvi18g02692\_t001 |  |  |  |  |  |  |  |  |
| 2 | Vitvi07g01767\_t001 |  | \| \| |  | \| \| |  |  |  |  |  |  |  |  |
| 2 | Vitvi07g01768\_t001 |  | \| \| |  | \| \| |  |  |  |  |  |  |  |  |
| 2 | Vitvi07g01769\_t001 |  | \| \| |  | Vitvi18g02703\_t001 |  |  |  |  |  |  |  |  |
| 2 | Vitvi07g01770\_t001 |  | \| \| |  | \| \| |  |  |  |  |  |  |  |  |
| 2 | Vitvi07g01772\_t001 |  | \| \| |  | \| \| |  |  |  |  |  |  |  |  |
| 2 | Vitvi07g04721\_t001 |  | \| \| |  | \| \| |  |  |  |  |  |  |  |  |
| 2 | Vitvi07g04722\_t001 |  | \| \| |  | \| \| |  |  |  |  |  |  |  |  |
| 2 | Vitvi07g01773\_t001 |  | Vitvi03g00477\_t001 |  | Vitvi18g00831\_t001 |  |  |  |  |  |  |  |  |
| 0 | Vitvi07g04723\_t001 |  |  |  |  |  |  |  |  |  |  |  |  |
| 0 | Vitvi07g02668\_t001 |  |  |  |  |  |  |  |  |  |  |  |  |
| 0 | Vitvi07g02670\_t001 |  |  |  |  |  |  |  |  |  |  |  |  |
| 0 | Vitvi07g04724\_t001 |  |  |  |  |  |  |  |  |  |  |  |  |
| 0 | Vitvi07g04725\_t001 |  |  |  |  |  |  |  |  |  |  |  |  |
| 0 | Vitvi07g04726\_t001 |  |  |  |  |  |  |  |  |  |  |  |  |
| 0 | Vitvi07g04727\_t001 |  |  |  |  |  |  |  |  |  |  |  |  |
| 0 | Vitvi07g01777\_t001 |  |  |  |  |  |  |  |  |  |  |  |  |
| 0 | Vitvi07g01778\_t001 |  |  |  |  |  |  |  |  |  |  |  |  |
| 1 | Vitvi07g01779\_t001 |  | Vitvi18g00363\_t001 |  |  |  |  |  |  |  |  |  |  |
| 1 | Vitvi07g01780\_t001 |  | \| \| |  |  |  |  |  |  |  |  |  |  |
| 1 | Vitvi07g01783\_t002 |  | Vitvi18g00362\_t001 |  |  |  |  |  |  |  |  |  |  |
| 1 | Vitvi07g02672\_t001 |  | \| \| |  |  |  |  |  |  |  |  |  |  |
| 1 | Vitvi07g02674\_t001 |  | \| \| |  |  |  |  |  |  |  |  |  |  |
| 1 | Vitvi07g02675\_t001 |  | \| \| |  |  |  |  |  |  |  |  |  |  |
| 1 | Vitvi07g02676\_t001 |  | \| \| |  |  |  |  |  |  |  |  |  |  |
| 1 | Vitvi07g04728\_t001 |  | \| \| |  |  |  |  |  |  |  |  |  |  |
| 1 | Vitvi07g01784\_t001 |  | \| \| |  |  |  |  |  |  |  |  |  |  |
| 1 | Vitvi07g01787\_t001 |  | \| \| |  |  |  |  |  |  |  |  |  |  |
| 1 | Vitvi07g04729\_t001 |  | \| \| |  |  |  |  |  |  |  |  |  |  |
| 1 | Vitvi07g01789\_t001 |  | \| \| |  |  |  |  |  |  |  |  |  |  |
| 1 | Vitvi07g04730\_t001 |  | \| \| |  |  |  |  |  |  |  |  |  |  |
| 1 | Vitvi07g01790\_t001 |  | \| \| |  |  |  |  |  |  |  |  |  |  |
| 1 | Vitvi07g01791\_t001 |  | \| \| |  |  |  |  |  |  |  |  |  |  |
| 1 | Vitvi07g04731\_t001 |  | \| \| |  |  |  |  |  |  |  |  |  |  |
| 1 | Vitvi07g01792\_t001 |  | Vitvi18g00361\_t001 |  |  |  |  |  |  |  |  |  |  |
| 1 | Vitvi07g04732\_t003 |  | \| \| |  |  |  |  |  |  |  |  |  |  |
| 1 | Vitvi07g01794\_t001 |  | Vitvi18g02559\_t001 |  |  |  |  |  |  |  |  |  |  |
| 1 | Vitvi07g01795\_t001 |  | Vitvi18g00360\_t001 |  |  |  |  |  |  |  |  |  |  |
| 1 | Vitvi07g01796\_t001 |  | \| \| |  |  |  |  |  |  |  |  |  |  |
| 1 | Vitvi07g01797\_t001 |  | \| \| |  |  |  |  |  |  |  |  |  |  |
| 1 | Vitvi07g01798\_t001 |  | \| \| |  |  |  |  |  |  |  |  |  |  |
| 1 | Vitvi07g01800\_t001 |  | Vitvi18g00359\_t001 |  |  |  |  |  |  |  |  |  |  |
| 1 | Vitvi07g01802\_t001 |  | Vitvi18g00358\_t002 |  |  |  |  |  |  |  |  |  |  |
| 1 | Vitvi07g01803\_t002 |  | \| \| |  |  |  |  |  |  |  |  |  |  |
| 1 | Vitvi07g01804\_t001 |  | \| \| |  |  |  |  |  |  |  |  |  |  |
| 1 | Vitvi07g02679\_t001 |  | \| \| |  |  |  |  |  |  |  |  |  |  |
| 1 | Vitvi07g02680\_t001 |  | \| \| |  |  |  |  |  |  |  |  |  |  |
| 1 | Vitvi07g02681\_t001 |  | \| \| |  |  |  |  |  |  |  |  |  |  |
| 1 | Vitvi07g01805\_t001 |  | \| \| |  |  |  |  |  |  |  |  |  |  |
| 1 | Vitvi07g02682\_t001 |  | \| \| |  |  |  |  |  |  |  |  |  |  |
| 1 | Vitvi07g01806\_t001 |  | \| \| |  |  |  |  |  |  |  |  |  |  |
| 1 | Vitvi07g02683\_t001 |  | \| \| |  |  |  |  |  |  |  |  |  |  |
| 1 | Vitvi07g02684\_t001 |  | \| \| |  |  |  |  |  |  |  |  |  |  |
| 1 | Vitvi07g04733\_t001 |  | \| \| |  |  |  |  |  |  |  |  |  |  |
| 1 | Vitvi07g01807\_t001 |  | Vitvi18g00346\_t001 |  |  |  |  |  |  |  |  |  |  |
| 0 | Vitvi07g01808\_t001 |  |  |  |  |  |  |  |  |  |  |  |  |
| 0 | Vitvi07g01809\_t001 |  |  |  |  |  |  |  |  |  |  |  |  |
| 0 | Vitvi07g01810\_t001 |  |  |  |  |  |  |  |  |  |  |  |  |
| 0 | Vitvi07g01811\_t001 |  |  |  |  |  |  |  |  |  |  |  |  |
| 0 | Vitvi07g01813\_t001 |  |  |  |  |  |  |  |  |  |  |  |  |
| 0 | Vitvi07g02686\_t001 |  |  |  |  |  |  |  |  |  |  |  |  |
| 0 | Vitvi07g01814\_t001 |  |  |  |  |  |  |  |  |  |  |  |  |
| 0 | Vitvi07g01816\_t001 |  |  |  |  |  |  |  |  |  |  |  |  |
| 0 | Vitvi07g01817\_t001 |  |  |  |  |  |  |  |  |  |  |  |  |
| 0 | Vitvi07g01818\_t001 |  |  |  |  |  |  |  |  |  |  |  |  |
| 0 | Vitvi07g04734\_t001 |  |  |  |  |  |  |  |  |  |  |  |  |
| 0 | Vitvi07g01821\_t001 |  |  |  |  |  |  |  |  |  |  |  |  |
| 0 | Vitvi07g01822\_t001 |  |  |  |  |  |  |  |  |  |  |  |  |
| 0 | Vitvi07g01823\_t001 |  |  |  |  |  |  |  |  |  |  |  |  |
| 0 | Vitvi07g01824\_t001 |  |  |  |  |  |  |  |  |  |  |  |  |
| 0 | Vitvi07g01825\_t001 |  |  |  |  |  |  |  |  |  |  |  |  |
| 0 | Vitvi07g01826\_t001 |  |  |  |  |  |  |  |  |  |  |  |  |
| 0 | Vitvi07g04735\_t001 |  |  |  |  |  |  |  |  |  |  |  |  |
| 0 | Vitvi07g01827\_t001 |  |  |  |  |  |  |  |  |  |  |  |  |
| 0 | Vitvi07g02687\_t001 |  |  |  |  |  |  |  |  |  |  |  |  |
| 0 | Vitvi07g01828\_t001 |  |  |  |  |  |  |  |  |  |  |  |  |
| 0 | Vitvi07g02688\_t001 |  |  |  |  |  |  |  |  |  |  |  |  |
| 0 | Vitvi07g01829\_t001 |  |  |  |  |  |  |  |  |  |  |  |  |
| 0 | Vitvi07g01830\_t001 |  |  |  |  |  |  |  |  |  |  |  |  |
| 0 | Vitvi07g01831\_t002 |  |  |  |  |  |  |  |  |  |  |  |  |
| 0 | Vitvi07g01832\_t001 |  |  |  |  |  |  |  |  |  |  |  |  |
| 0 | Vitvi07g04736\_t001 |  |  |  |  |  |  |  |  |  |  |  |  |
| 0 | Vitvi07g04737\_t001 |  |  |  |  |  |  |  |  |  |  |  |  |
| 0 | Vitvi07g02689\_t001 |  |  |  |  |  |  |  |  |  |  |  |  |
| 1 | Vitvi07g01835\_t001 |  | Vitvi18g01517\_t001 |  |  |  |  |  |  |  |  |  |  |
| 1 | Vitvi07g01836\_t001 |  | \| \| |  |  |  |  |  |  |  |  |  |  |
| 1 | Vitvi07g01837\_t001 |  | \| \| |  |  |  |  |  |  |  |  |  |  |
| 1 | Vitvi07g01838\_t001 |  | \| \| |  |  |  |  |  |  |  |  |  |  |
| 1 | Vitvi07g04738\_t001 |  | \| \| |  |  |  |  |  |  |  |  |  |  |
| 1 | Vitvi07g02691\_t001 |  | \| \| |  |  |  |  |  |  |  |  |  |  |
| 1 | Vitvi07g01840\_t001 |  | \| \| |  |  |  |  |  |  |  |  |  |  |
| 1 | Vitvi07g01841\_t001.2.6037826f |  | \| \| |  |  |  |  |  |  |  |  |  |  |
| 1 | Vitvi07g01842\_t001 |  | \| \| |  |  |  |  |  |  |  |  |  |  |
| 1 | Vitvi07g02692\_t001 |  | \| \| |  |  |  |  |  |  |  |  |  |  |
| 1 | Vitvi07g01843\_t001 |  | \| \| |  |  |  |  |  |  |  |  |  |  |
| 1 | Vitvi07g04739\_t001 |  | \| \| |  |  |  |  |  |  |  |  |  |  |
| 1 | Vitvi07g01844\_t001 |  | \| \| |  |  |  |  |  |  |  |  |  |  |
| 1 | Vitvi07g01845\_t001 |  | \| \| |  |  |  |  |  |  |  |  |  |  |
| 1 | Vitvi07g01846\_t001 |  | Vitvi18g01531\_t001 |  |  |  |  |  |  |  |  |  |  |
| 1 | Vitvi07g01847\_t001 |  | \| \| |  |  |  |  |  |  |  |  |  |  |
| 1 | Vitvi07g01848\_t001 |  | Vitvi18g01535\_t001 |  |  |  |  |  |  |  |  |  |  |
| 1 | Vitvi07g01849\_t001 |  | \| \| |  |  |  |  |  |  |  |  |  |  |
| 1 | Vitvi07g01850\_t001 |  | \| \| |  |  |  |  |  |  |  |  |  |  |
| 1 | Vitvi07g01851\_t001 |  | \| \| |  |  |  |  |  |  |  |  |  |  |
| 1 | Vitvi07g01852\_t001 |  | \| \| |  |  |  |  |  |  |  |  |  |  |
| 1 | Vitvi07g01853\_t001 |  | \| \| |  |  |  |  |  |  |  |  |  |  |
| 1 | Vitvi07g01855\_t001 |  | \| \| |  |  |  |  |  |  |  |  |  |  |
| 1 | Vitvi07g01856\_t001 |  | \| \| |  |  |  |  |  |  |  |  |  |  |
| 1 | Vitvi07g01857\_t001 |  | Vitvi18g01571\_t001 |  |  |  |  |  |  |  |  |  |  |
| 1 | Vitvi07g01858\_t002 |  | \| \| |  |  |  |  |  |  |  |  |  |  |
| 1 | Vitvi07g01859\_t001 |  | \| \| |  |  |  |  |  |  |  |  |  |  |
| 1 | Vitvi07g01860\_t001 |  | \| \| |  |  |  |  |  |  |  |  |  |  |
| 1 | Vitvi07g01861\_t001 |  | \| \| |  |  |  |  |  |  |  |  |  |  |
| 1 | Vitvi07g01862\_t001 |  | Vitvi18g01602\_t001 |  |  |  |  |  |  |  |  |  |  |
| 1 | Vitvi07g02693\_t001 |  | \| \| |  |  |  |  |  |  |  |  |  |  |
| 1 | Vitvi07g01863\_t001 |  | \| \| |  |  |  |  |  |  |  |  |  |  |
| 1 | Vitvi07g01864\_t001 |  | \| \| |  |  |  |  |  |  |  |  |  |  |
| 1 | Vitvi07g01865\_t001 |  | \| \| |  |  |  |  |  |  |  |  |  |  |
| 1 | Vitvi07g01866\_t001 |  | \| \| |  |  |  |  |  |  |  |  |  |  |
| 1 | Vitvi07g01867\_t001 |  | \| \| |  |  |  |  |  |  |  |  |  |  |
| 1 | Vitvi07g04740\_t001 |  | Vitvi18g04416\_t001 |  |  |  |  |  |  |  |  |  |  |
| 1 | Vitvi07g01869\_t001 |  | \| \| |  |  |  |  |  |  |  |  |  |  |
| 1 | Vitvi07g01870\_t001 |  | \| \| |  |  |  |  |  |  |  |  |  |  |
| 1 | Vitvi07g02694\_t001 |  | \| \| |  |  |  |  |  |  |  |  |  |  |
| 1 | Vitvi07g04741\_t001 |  | \| \| |  |  |  |  |  |  |  |  |  |  |
| 1 | Vitvi07g04742\_t001 |  | \| \| |  |  |  |  |  |  |  |  |  |  |
| 1 | Vitvi07g01871\_t001 |  | Vitvi18g01614\_t001 |  |  |  |  |  |  |  |  |  |  |
| 1 | Vitvi07g01872\_t001 |  | \| \| |  |  |  |  |  |  |  |  |  |  |
| 1 | Vitvi07g01874\_t001 |  | Vitvi18g01617\_t001 |  |  |  |  |  |  |  |  |  |  |
| 1 | Vitvi07g01875\_t001 |  | \| \| |  |  |  |  |  |  |  |  |  |  |
| 1 | Vitvi07g02696\_t001 |  | \| \| |  |  |  |  |  |  |  |  |  |  |
| 1 | Vitvi07g04743\_t001 |  | \| \| |  |  |  |  |  |  |  |  |  |  |
| 1 | Vitvi07g01876\_t001 |  | \| \| |  |  |  |  |  |  |  |  |  |  |
| 1 | Vitvi07g01877\_t001 |  | \| \| |  |  |  |  |  |  |  |  |  |  |
| 1 | Vitvi07g01878\_t001 |  | Vitvi18g01621\_t001 |  |  |  |  |  |  |  |  |  |  |
| 1 | Vitvi07g01879\_t001 |  | Vitvi18g01624\_t001 |  |  |  |  |  |  |  |  |  |  |
| 1 | Vitvi07g01880\_t001 |  | Vitvi18g01625\_t001 |  |  |  |  |  |  |  |  |  |  |
| 1 | Vitvi07g02697\_t001 |  | \| \| |  |  |  |  |  |  |  |  |  |  |
| 1 | Vitvi07g01881\_t001 |  | Vitvi18g01628\_t001 |  |  |  |  |  |  |  |  |  |  |
| 1 | Vitvi07g01882\_t001 |  | Vitvi18g02972\_t001 |  |  |  |  |  |  |  |  |  |  |
| 1 | Vitvi07g01883\_t001 |  | Vitvi18g01631\_t001 |  |  |  |  |  |  |  |  |  |  |
| 0 | Vitvi07g02698\_t001 |  |  |  |  |  |  |  |  |  |  |  |  |
| 0 | Vitvi07g01884\_t004 |  |  |  |  |  |  |  |  |  |  |  |  |
| 0 | Vitvi07g01885\_t001 |  |  |  |  |  |  |  |  |  |  |  |  |
| 0 | Vitvi07g01886\_t002 |  |  |  |  |  |  |  |  |  |  |  |  |
| 0 | Vitvi07g01887\_t001 |  |  |  |  |  |  |  |  |  |  |  |  |
| 0 | Vitvi07g01888\_t001 |  |  |  |  |  |  |  |  |  |  |  |  |
| 1 | Vitvi07g01889\_t001 |  | Vitvi18g00072\_t005 |  |  |  |  |  |  |  |  |  |  |
| 1 | Vitvi07g04744\_t001 |  | \| \| |  |  |  |  |  |  |  |  |  |  |
| 1 | Vitvi07g01890\_t001 |  | Vitvi18g00071\_t001 |  |  |  |  |  |  |  |  |  |  |
| 1 | Vitvi07g01891\_t001 |  | Vitvi18g00069\_t001 |  |  |  |  |  |  |  |  |  |  |
| 1 | Vitvi07g01892\_t001 |  | \| \| |  |  |  |  |  |  |  |  |  |  |
| 1 | Vitvi07g01893\_t001 |  | \| \| |  |  |  |  |  |  |  |  |  |  |
| 1 | Vitvi07g01894\_t001 |  | Vitvi18g00067\_t001 |  |  |  |  |  |  |  |  |  |  |
| 1 | Vitvi07g04745\_t001 |  | Vitvi18g00065\_t001 |  |  |  |  |  |  |  |  |  |  |
| 1 | Vitvi07g01896\_t001 |  | Vitvi18g00061\_t001 |  |  |  |  |  |  |  |  |  |  |
| 1 | Vitvi07g02702\_t001 |  | Vitvi18g00058\_t001 |  |  |  |  |  |  |  |  |  |  |
| 1 | Vitvi07g01897\_t001 |  | \| \| |  |  |  |  |  |  |  |  |  |  |
| 1 | Vitvi07g01898\_t001 |  | Vitvi18g00056\_t002 |  |  |  |  |  |  |  |  |  |  |
| 0 | Vitvi07g01899\_t001 |  |  |  |  |  |  |  |  |  |  |  |  |
| 0 | Vitvi07g01900\_t001 |  |  |  |  |  |  |  |  |  |  |  |  |
| 0 | Vitvi07g01901\_t001 |  |  |  |  |  |  |  |  |  |  |  |  |
| 0 | Vitvi07g01902\_t001 |  |  |  |  |  |  |  |  |  |  |  |  |
| 0 | Vitvi07g04746\_t001 |  |  |  |  |  |  |  |  |  |  |  |  |
| 0 | Vitvi07g01903\_t001 |  |  |  |  |  |  |  |  |  |  |  |  |
| 0 | Vitvi07g04747\_t001 |  |  |  |  |  |  |  |  |  |  |  |  |
| 0 | Vitvi07g01905\_t001 |  |  |  |  |  |  |  |  |  |  |  |  |
| 0 | Vitvi07g01906\_t001 |  |  |  |  |  |  |  |  |  |  |  |  |
| 1 | Vitvi07g02703\_t001 |  | Vitvi18g00234\_t001 |  |  |  |  |  |  |  |  |  |  |
| 1 | Vitvi07g04748\_t001 |  | \| \| |  |  |  |  |  |  |  |  |  |  |
| 1 | Vitvi07g04749\_t001 |  | \| \| |  |  |  |  |  |  |  |  |  |  |
| 1 | Vitvi07g01908\_t001 |  | \| \| |  |  |  |  |  |  |  |  |  |  |
| 1 | Vitvi07g01909\_t001 |  | \| \| |  |  |  |  |  |  |  |  |  |  |
| 1 | Vitvi07g01910\_t002 |  | \| \| |  |  |  |  |  |  |  |  |  |  |
| 1 | Vitvi07g01911\_t001 |  | \| \| |  |  |  |  |  |  |  |  |  |  |
| 1 | Vitvi07g04750\_t001 |  | Vitvi18g00237\_t001 |  |  |  |  |  |  |  |  |  |  |
| 1 | Vitvi07g02707\_t001 |  | \| \| |  |  |  |  |  |  |  |  |  |  |
| 1 | Vitvi07g01913\_t001 |  | \| \| |  |  |  |  |  |  |  |  |  |  |
| 1 | Vitvi07g04751\_t001 |  | \| \| |  |  |  |  |  |  |  |  |  |  |
| 1 | Vitvi07g01916\_t001 |  | \| \| |  |  |  |  |  |  |  |  |  |  |
| 1 | Vitvi07g01917\_t001 |  | \| \| |  |  |  |  |  |  |  |  |  |  |
| 1 | Vitvi07g04752\_t001 |  | \| \| |  |  |  |  |  |  |  |  |  |  |
| 1 | Vitvi07g02708\_t001 |  | \| \| |  |  |  |  |  |  |  |  |  |  |
| 1 | Vitvi07g01918\_t001 |  | \| \| |  |  |  |  |  |  |  |  |  |  |
| 1 | Vitvi07g01919\_t001.1.6037826f |  | \| \| |  |  |  |  |  |  |  |  |  |  |
| 1 | Vitvi07g01920\_t001 |  | \| \| |  |  |  |  |  |  |  |  |  |  |
| 1 | Vitvi07g04753\_t001 |  | \| \| |  |  |  |  |  |  |  |  |  |  |
| 1 | Vitvi07g04754\_t001 |  | \| \| |  |  |  |  |  |  |  |  |  |  |
| 1 | Vitvi07g04755\_t001 |  | \| \| |  |  |  |  |  |  |  |  |  |  |
| 1 | Vitvi07g01921\_t001 |  | \| \| |  |  |  |  |  |  |  |  |  |  |
| 1 | Vitvi07g01923\_t001 |  | \| \| |  |  |  |  |  |  |  |  |  |  |
| 1 | Vitvi07g01924\_t001 |  | \| \| |  |  |  |  |  |  |  |  |  |  |
| 1 | Vitvi07g01927\_t001 |  | Vitvi18g00245\_t001 |  |  |  |  |  |  |  |  |  |  |
| 1 | Vitvi07g01929\_t001 |  | Vitvi18g00250\_t001 |  |  |  |  |  |  |  |  |  |  |
| 1 | Vitvi07g01930\_t001 |  | Vitvi18g00254\_t001 |  |  |  |  |  |  |  |  |  |  |
| 1 | Vitvi07g04756\_t001 |  | \| \| |  |  |  |  |  |  |  |  |  |  |
| 1 | Vitvi07g01932\_t001 |  | \| \| |  |  |  |  |  |  |  |  |  |  |
| 1 | Vitvi07g01934\_t002 |  | Vitvi18g00255\_t001 |  |  |  |  |  |  |  |  |  |  |
| 1 | Vitvi07g01936\_t001 |  | \| \| |  |  |  |  |  |  |  |  |  |  |
| 1 | Vitvi07g01937\_t002 |  | \| \| |  |  |  |  |  |  |  |  |  |  |
| 1 | Vitvi07g01941\_t001 |  | Vitvi18g00257\_t001 |  |  |  |  |  |  |  |  |  |  |
| 1 | Vitvi07g01942\_t001 |  | Vitvi18g00258\_t001 |  |  |  |  |  |  |  |  |  |  |
| 1 | Vitvi07g01943\_t001 |  | \| \| |  |  |  |  |  |  |  |  |  |  |
| 1 | Vitvi07g01946\_t001 |  | \| \| |  |  |  |  |  |  |  |  |  |  |
| 1 | Vitvi07g04757\_t001 |  | \| \| |  |  |  |  |  |  |  |  |  |  |
| 1 | Vitvi07g01947\_t001 |  | \| \| |  |  |  |  |  |  |  |  |  |  |
| 1 | Vitvi07g01948\_t002 |  | \| \| |  |  |  |  |  |  |  |  |  |  |
| 1 | Vitvi07g01949\_t003 |  | Vitvi18g00271\_t001 |  |  |  |  |  |  |  |  |  |  |
| 1 | Vitvi07g01950\_t001 |  | \| \| |  |  |  |  |  |  |  |  |  |  |
| 1 | Vitvi07g01951\_t001 |  | \| \| |  |  |  |  |  |  |  |  |  |  |
| 1 | Vitvi07g01952\_t001 |  | \| \| |  |  |  |  |  |  |  |  |  |  |
| 1 | Vitvi07g01954\_t001 |  | \| \| |  |  |  |  |  |  |  |  |  |  |
| 1 | Vitvi07g04758\_t001 |  | \| \| |  |  |  |  |  |  |  |  |  |  |
| 1 | Vitvi07g01955\_t001 |  | Vitvi18g02532\_t001 |  |  |  |  |  |  |  |  |  |  |
| 1 | Vitvi07g01956\_t001 |  | \| \| |  |  |  |  |  |  |  |  |  |  |
| 1 | Vitvi07g02711\_t001 |  | \| \| |  |  |  |  |  |  |  |  |  |  |
| 1 | Vitvi07g04759\_t001 |  | \| \| |  |  |  |  |  |  |  |  |  |  |
| 1 | Vitvi07g01957\_t003 |  | \| \| |  |  |  |  |  |  |  |  |  |  |
| 1 | Vitvi07g01958\_t001 |  | \| \| |  |  |  |  |  |  |  |  |  |  |
| 1 | Vitvi07g04760\_t001 |  | \| \| |  |  |  |  |  |  |  |  |  |  |
| 1 | Vitvi07g01959\_t001 |  | \| \| |  |  |  |  |  |  |  |  |  |  |
| 1 | Vitvi07g01961\_t001 |  | \| \| |  |  |  |  |  |  |  |  |  |  |
| 1 | Vitvi07g04761\_t001 |  | \| \| |  |  |  |  |  |  |  |  |  |  |
| 1 | Vitvi07g01964\_t001 |  | \| \| |  |  |  |  |  |  |  |  |  |  |
| 1 | Vitvi07g01965\_t001 |  | \| \| |  |  |  |  |  |  |  |  |  |  |
| 1 | Vitvi07g04762\_t001 |  | \| \| |  |  |  |  |  |  |  |  |  |  |
| 1 | Vitvi07g01966\_t001 |  | \| \| |  |  |  |  |  |  |  |  |  |  |
| 1 | Vitvi07g04763\_t001 |  | \| \| |  |  |  |  |  |  |  |  |  |  |
| 1 | Vitvi07g02714\_t001 |  | \| \| |  |  |  |  |  |  |  |  |  |  |
| 1 | Vitvi07g01969\_t001 |  | \| \| |  |  |  |  |  |  |  |  |  |  |
| 1 | Vitvi07g01970\_t001 |  | Vitvi18g00284\_t001 |  |  |  |  |  |  |  |  |  |  |
| 0 | Vitvi07g01973\_t001 |  |  |  |  |  |  |  |  |  |  |  |  |
| 0 | Vitvi07g01977\_t001 |  |  |  |  |  |  |  |  |  |  |  |  |
| 0 | Vitvi07g01978\_t001 |  |  |  |  |  |  |  |  |  |  |  |  |
| 0 | Vitvi07g04764\_t001 |  |  |  |  |  |  |  |  |  |  |  |  |
| 0 | Vitvi07g01979\_t001 |  |  |  |  |  |  |  |  |  |  |  |  |
| 0 | Vitvi07g01980\_t001 |  |  |  |  |  |  |  |  |  |  |  |  |
| 0 | Vitvi07g01981\_t001 |  |  |  |  |  |  |  |  |  |  |  |  |
| 0 | Vitvi07g01982\_t001 |  |  |  |  |  |  |  |  |  |  |  |  |
| 0 | Vitvi07g01983\_t001 |  |  |  |  |  |  |  |  |  |  |  |  |
| 0 | Vitvi07g01984\_t005 |  |  |  |  |  |  |  |  |  |  |  |  |
| 0 | Vitvi07g01985\_t001 |  |  |  |  |  |  |  |  |  |  |  |  |
| 0 | Vitvi07g01988\_t001 |  |  |  |  |  |  |  |  |  |  |  |  |
| 0 | Vitvi07g01989\_t001 |  |  |  |  |  |  |  |  |  |  |  |  |
| 0 | Vitvi07g01990\_t001 |  |  |  |  |  |  |  |  |  |  |  |  |
| 0 | Vitvi07g02716\_t001 |  |  |  |  |  |  |  |  |  |  |  |  |
| 0 | Vitvi07g02717\_t001 |  |  |  |  |  |  |  |  |  |  |  |  |
| 0 | Vitvi07g01991\_t001 |  |  |  |  |  |  |  |  |  |  |  |  |
| 0 | Vitvi07g01993\_t001 |  |  |  |  |  |  |  |  |  |  |  |  |
| 0 | Vitvi07g04765\_t001 |  |  |  |  |  |  |  |  |  |  |  |  |
| 0 | Vitvi07g01994\_t001 |  |  |  |  |  |  |  |  |  |  |  |  |
| 0 | Vitvi07g04766\_t001 |  |  |  |  |  |  |  |  |  |  |  |  |
| 0 | Vitvi07g04767\_t001 |  |  |  |  |  |  |  |  |  |  |  |  |
| 0 | Vitvi07g01995\_t001 |  |  |  |  |  |  |  |  |  |  |  |  |
| 0 | Vitvi07g01996\_t004 |  |  |  |  |  |  |  |  |  |  |  |  |
| 0 | Vitvi07g04768\_t001 |  |  |  |  |  |  |  |  |  |  |  |  |
| 0 | Vitvi07g01998\_t001 |  |  |  |  |  |  |  |  |  |  |  |  |
| 0 | Vitvi07g01999\_t001 |  |  |  |  |  |  |  |  |  |  |  |  |
| 0 | Vitvi07g02000\_t001 |  |  |  |  |  |  |  |  |  |  |  |  |
| 0 | Vitvi07g02001\_t001 |  |  |  |  |  |  |  |  |  |  |  |  |
| 0 | Vitvi07g04769\_t001 |  |  |  |  |  |  |  |  |  |  |  |  |
| 0 | Vitvi07g02003\_t001 |  |  |  |  |  |  |  |  |  |  |  |  |
| 0 | Vitvi07g02005\_t001 |  |  |  |  |  |  |  |  |  |  |  |  |
| 0 | Vitvi07g02006\_t001 |  |  |  |  |  |  |  |  |  |  |  |  |
| 0 | Vitvi07g04770\_t001 |  |  |  |  |  |  |  |  |  |  |  |  |
| 0 | Vitvi07g02720\_t001 |  |  |  |  |  |  |  |  |  |  |  |  |
| 0 | Vitvi07g02007\_t002 |  |  |  |  |  |  |  |  |  |  |  |  |
| 0 | Vitvi07g02009\_t001 |  |  |  |  |  |  |  |  |  |  |  |  |
| 0 | Vitvi07g02010\_t001 |  |  |  |  |  |  |  |  |  |  |  |  |
| 0 | Vitvi07g02011\_t001 |  |  |  |  |  |  |  |  |  |  |  |  |
| 0 | Vitvi07g02012\_t001 |  |  |  |  |  |  |  |  |  |  |  |  |
| 0 | Vitvi07g02013\_t001 |  |  |  |  |  |  |  |  |  |  |  |  |
| 0 | Vitvi07g02014\_t001 |  |  |  |  |  |  |  |  |  |  |  |  |
| 0 | Vitvi07g02015\_t001 |  |  |  |  |  |  |  |  |  |  |  |  |
| 0 | Vitvi07g02721\_t001 |  |  |  |  |  |  |  |  |  |  |  |  |
| 0 | Vitvi07g02722\_t001 |  |  |  |  |  |  |  |  |  |  |  |  |
| 0 | Vitvi07g02016\_t001 |  |  |  |  |  |  |  |  |  |  |  |  |
| 0 | Vitvi07g02019\_t001 |  |  |  |  |  |  |  |  |  |  |  |  |
| 0 | Vitvi07g02020\_t001 |  |  |  |  |  |  |  |  |  |  |  |  |
| 0 | Vitvi07g02021\_t001 |  |  |  |  |  |  |  |  |  |  |  |  |
| 0 | Vitvi07g02022\_t001 |  |  |  |  |  |  |  |  |  |  |  |  |
| 0 | Vitvi07g02723\_t001 |  |  |  |  |  |  |  |  |  |  |  |  |
| 0 | Vitvi07g02023\_t001 |  |  |  |  |  |  |  |  |  |  |  |  |
| 0 | Vitvi07g04771\_t001 |  |  |  |  |  |  |  |  |  |  |  |  |
| 0 | Vitvi07g04772\_t001 |  |  |  |  |  |  |  |  |  |  |  |  |
| 0 | Vitvi07g02028\_t001 |  |  |  |  |  |  |  |  |  |  |  |  |
| 0 | Vitvi07g02029\_t001 |  |  |  |  |  |  |  |  |  |  |  |  |
| 0 | Vitvi07g02031\_t001 |  |  |  |  |  |  |  |  |  |  |  |  |
| 0 | Vitvi07g02032\_t001 |  |  |  |  |  |  |  |  |  |  |  |  |
| 0 | Vitvi07g02034\_t001 |  |  |  |  |  |  |  |  |  |  |  |  |
| 0 | Vitvi07g04773\_t001 |  |  |  |  |  |  |  |  |  |  |  |  |
| 0 | Vitvi07g02035\_t001 |  |  |  |  |  |  |  |  |  |  |  |  |
| 0 | Vitvi07g02036\_t001 |  |  |  |  |  |  |  |  |  |  |  |  |
| 0 | Vitvi07g02038\_t001 |  |  |  |  |  |  |  |  |  |  |  |  |
| 0 | Vitvi07g02039\_t001 |  |  |  |  |  |  |  |  |  |  |  |  |
| 0 | Vitvi07g02724\_t001 |  |  |  |  |  |  |  |  |  |  |  |  |
| 0 | Vitvi07g02040\_t001 |  |  |  |  |  |  |  |  |  |  |  |  |
| 0 | Vitvi07g02041\_t001 |  |  |  |  |  |  |  |  |  |  |  |  |
| 0 | Vitvi07g02042\_t001 |  |  |  |  |  |  |  |  |  |  |  |  |
| 0 | Vitvi07g02043\_t001 |  |  |  |  |  |  |  |  |  |  |  |  |
| 0 | Vitvi07g04774\_t001 |  |  |  |  |  |  |  |  |  |  |  |  |
| 0 | Vitvi07g02045\_t001 |  |  |  |  |  |  |  |  |  |  |  |  |
| 0 | Vitvi07g04775\_t001 |  |  |  |  |  |  |  |  |  |  |  |  |
| 0 | Vitvi07g02047\_t001 |  |  |  |  |  |  |  |  |  |  |  |  |
| 0 | Vitvi07g02048\_t001 |  |  |  |  |  |  |  |  |  |  |  |  |
| 0 | Vitvi07g02049\_t001 |  |  |  |  |  |  |  |  |  |  |  |  |
| 0 | Vitvi07g02050\_t001 |  |  |  |  |  |  |  |  |  |  |  |  |
| 0 | Vitvi07g02053\_t001 |  |  |  |  |  |  |  |  |  |  |  |  |
| 0 | Vitvi07g02055\_t001 |  |  |  |  |  |  |  |  |  |  |  |  |
